# Integrative Genomics Identifies Gene Signature Associated with Melanoma Ulceration

**DOI:** 10.1371/journal.pone.0054958

**Published:** 2013-01-30

**Authors:** Zsuzsa Rakosy, Szilvia Ecsedi, Reka Toth, Laura Vizkeleti, Hector Herandez-Vargas, Viktoria Lazar, Gabriella Emri, Istvan Szatmari, Zdenko Herceg, Roza Adany, Margit Balazs

**Affiliations:** 1 Department of Preventive Medicine, Faculty of Public Health, Medical and Health Science Center, University of Debrecen, Debrecen, Hungary; 2 Public Health Research Group of the Hungarian Academy of Sciences, University of Debrecen, Debrecen, Hungary; 3 Department of Dermatology, Faculty of Medicine, Medical and Health Science Center, University of Debrecen, Debrecen, Hungary; 4 Department of Biochemistry and Molecular Biology, Faculty of Medicine, Medical and Health Science Center, University of Debrecen, Debrecen, Hungary; 5 World Health Organization International Agency for Research on Cancer, Epigenetics Group, Lyon, France; Geisel School of Medicine at Dartmouth, United States of America

## Abstract

**Background:**

Despite the extensive research approaches applied to characterise malignant melanoma, no specific molecular markers are available that are clearly related to the progression of this disease. In this study, our aims were to define a gene expression signature associated with the clinical outcome of melanoma patients and to provide an integrative interpretation of the gene expression -, copy number alterations -, and promoter methylation patterns that contribute to clinically relevant molecular functional alterations.

**Methods:**

Gene expression profiles were determined using the Affymetrix U133 Plus2.0 array. The NimbleGen Human CGH Whole-Genome Tiling array was used to define CNAs, and the Illumina GoldenGate Methylation platform was applied to characterise the methylation patterns of overlapping genes.

**Results:**

We identified two subclasses of primary melanoma: one representing patients with better prognoses and the other being characteristic of patients with unfavourable outcomes. We assigned 1,080 genes as being significantly correlated with ulceration, 987 genes were downregulated and significantly enriched in the p53, Nf-kappaB, and WNT/beta-catenin pathways. Through integrated genome analysis, we defined 150 downregulated genes whose expression correlated with copy number losses in ulcerated samples. These genes were significantly enriched on chromosome 6q and 10q, which contained a total of 36 genes. Ten of these genes were downregulated and involved in cell-cell and cell-matrix adhesion or apoptosis. The expression and methylation patterns of additional genes exhibited an inverse correlation, suggesting that transcriptional silencing of these genes is driven by epigenetic events.

**Conclusion:**

Using an integrative genomic approach, we were able to identify functionally relevant molecular hotspots characterised by copy number losses and promoter hypermethylation in distinct molecular subtypes of melanoma that contribute to specific transcriptomic silencing and might indicate a poor clinical outcome of melanoma.

## Introduction

Melanoma is an aggressive, therapy-resistant malignancy of the melanocytes. The incidence of this disease has been increasing worldwide, resulting in a growing public health problem [1], [2]. Interactions between molecular and environmental risk factors that promote melanomagenesis are the subject of ongoing research [3]. There are currently no systemic therapies available to significantly extend the life expectancy of patients with advanced melanoma. Therefore, early diagnosis and conventional treatments remain the key to improved survival for all affected individuals [1], [4], [5]. Recently, several high-throughput genomic and gene expression studies have been designed to reveal the molecular mechanisms involved in melanoma progression [6]–[14].

DNA microarray technology, a high-throughput screening methodology, offers an opportunity to simultaneously analyse the gene expression levels of thousands of genes in the cancer genome. This technology has been extensively used in cancer research to identify tumour subclasses, predict disease outcomes and identify genes associated with drug resistance [15], [16]. Previous transcriptome analyses have provided valuable information for the assessment of patient group classifications, such as subgroups of patients who are likely to respond to a particular therapy [17].

Many research groups have used different gene expression platforms to obtain a better understanding of the stepwise tumourigenesis involved in melanoma development. These studies have identified cohorts of genes that facilitate distinction of benign nevi from malignant melanomas [18], [19], sub-classification of metastatic melanomas into distinct subgroups [6], [19]–[23] and prediction of distant metastasis-free survival [20], [24]. Most of these research groups have found at least two naturally occurring subgroups using unsupervised clustering. Some researchers have been unable to find any association between subgroups with any clinical variables [19], [21]. However, other groups found that the clusters differed in terms of how advanced tumour were, defined by thickness and survival [20], and the transition from the radial growth phase (RGP) to vertical growth phase (VGP) [6], [22], [23]. Microarray studies have been limited in utility because of the lack of concordance from one study to the next. This limitation suggests that in addition to tumour heterogeneity, inconsistencies in the experimental design, sample preparation and platforms used for analysis have contributed to the diversity of data.

It has recently become apparent that melanoma heterogeneity may be caused by both alterations in gene expression and distinct genomic changes [25]–[29]. Taking a global view of the available malignant genome and transcriptome has proven to be valuable for understanding the intricate process of neoplastic transformation and for obtaining profound downstream benefits from these discoveries. Despite rapid progress in exploring commonly amplified and deleted regions and epigenetic changes in melanoma, integrated analyses have focused only on melanoma cell lines. Simultaneous studies have been aimed at exploring commonly affected genes that exhibit copy number changes and altered gene expression [30]–[32], but there has been no study conducted on melanoma tissues [33].

In this study, our aim was to define the gene expression signature of previously untreated primary melanomas in patients with a known clinical history using the Affymetrix GeneChip Human Genome U133 Plus 2.0 expression arrays platform. We extended these analyses and correlated the transcriptomic data with copy number alterations defined using the NimbleGen HG18 CGH 4x72K WG Tiling v2.0 array and the CpG methylation of promoters analysed by the Illumina GoldenGate Methylation Assay.

Integrated analysis of different types of data that are characteristic of the same tumour might allow us to identify molecular pathways and genetic aberrations that could influence the biological and clinical behaviour of melanomas and pinpoint progression-related genes, facilitating molecular classification of the disease. These candidate genes could ultimately be utilised as therapeutic targets for treating human malignant melanoma.

## Materials and Methods

### Melanoma Samples

Thirty-six primary melanomas were involved in our gene expression studies and 17 out of the 36 primary melanomas was analysed by tiling array CGH and DNA methylation assay. Tumour tissues were obtained from the Department of Dermatology, University of Debrecen, Hungary. All human studies were conducted in accordance with principles outlined in the Declaration of Helsinki and were approved by the Regional and Institutional Ethics Committee of the University of Debrecen Medical and Health Science Center and was conducted according to regulations (Protocol #2836-2008). Written informed consent was obtained from each patient. The clinicopathological data on the 36 primary melanomas are summarised in Table 1 according to the new melanoma TNM staging system [34], [35].

**Table 1:** Clinical and histopathological parameters of primary melanomas (N = 36).

Variables	No. of primary melanomas
Tumor type^a^	
NM	15
SSM	19
In situ	2
Gender	
Male	16
Female	20
Age (years)	
20–50	2516613127
>50	29
Breslow thickness (mm)^b^	
<2.00	18
2.01–4.00	6
>4.01	12
Clark's level	
I, II, III	20
IV, V	16
Ulceration	
Absent	18
Present	18
Localisation	
Trunk	12
Extremities	22
Head	3
Metastasis formation^c^	
Non-metastatic	29
Metastatic	7

a NM: nodular melanoma, SSM: superficial spreading melanoma.

b Thickness categories based on the current melanoma staging system.

c Metastases within 5 years after the removal of the primary tumour.

High quality total RNA and genomic DNA were isolated from fresh tissues. Detailed protocols for routine procedures and quality controls are available under the Text S1.

### Microarray Based Experiments

#### Gene expression analysis

Gene expression analysis was carried out using Affymetrix GeneChip Human Genome U133 Plus 2.0 expression arrays The microarray data was published in the Array Express Archive repository (a MIAME compliant database) with accession number E-MTAB-946.

Analyses of gene expression data were performed using GeneSpring 7.3 (Agilent Technologies, Santa Clara, CA, USA) and BRB Array Tools 4.3.0 developed by Dr. Richard Simon and BRB Array Tools Development Team. The raw intensity values from each chip were normalised to the 50th percentile of the measurements to reduce chip-wide variations in intensity. Each gene was normalised to the average level of that particular gene to allow comparison of relative changes in gene expression levels between different conditions. Only genes showing detection flag present in at least 50% of the samples were used in further analyses (25,886 probe sets).

The log_2_ transformed data of all samples were classified according to their expression pattern via hierarchical clustering using average linkage and Pearson’s correlation. The genes, that expressed differently among the predefined melanoma groups (Table 1) at the 2- fold change and p≤0.001 level after Benjamini and Hochberg multiple testing correction, were assessed by univariate t-test for each gene and were visualized by Volcano plots. Principal component analysis (PCA) of differentially expressed genes was applied to verify the proportion and the significance of the first 3 components covering the total variation.

Kaplan-Meier survival curves and Cox proportional univariate test were performed to whether the gene expression of a particular gene significantly influences survival at the p<0.001 level. To control for patients’ age, gender and clinical covariates on survival and to predict a survival prediction we used the Penalized Cox Regression model.

### Copy Number Analysis by Array CGH

An array CGH (NimbleGen HG18 CGH 4x72K WG Tiling v2.0) and the calculation of ratio values were performed on genomic DNA samples by Roche NimbleGen (Reykjavik, Iceland). All of the aCGH data was published in the Array Express Archive repository (a MIAME compliant database) with accession number E-MTAB-947.

Statistical analyses were conducted with Nexus Copy Number 5.1. For segmentation, we applied the FASST2 algorithm. To adjust the sensitivity of the segmentation algorithm, we determined a significance threshold of 1.0E-6 and specified 1000 kb as the maximum spacing between adjacent probes. The minimum number of probes per segment was 5 to eliminate small CNAs. To detect gains and losses, we set the thresholds as ±0.3 for gains and losses.

Significantly different copy number events between ulcerated versus non-ulcerated melanomas were identified by applying Fisher’s exact test, and FDR adjustment for correcting multiple testing was calculated using the Nexus Copy Number 5.1 Comparison feature.

### Investigation of the Promoter Methylation Patterns

The quantitative methylation status of the 1505 CpG sites corresponding to 807 cancer-related gene promoters corresponding to 14 primary melanomas was performed with the Illumina GoldenGate DNA Methylation arrays as described previously [36]. Briefly, genomic DNA from tumour tissues was prepared by overnight proteinase K treatment, phenol-chloroform extraction, and ethanol precipitation. Sodium bisulfite modification was performed on 500 ng DNA using the EZ DNA Methylation-Gold Kit (Zymo Research). BeadStudio v3.2 software (Illumina) was used for initial filtering and clustering analysis as described previously [36]. We identified 45 overlapping genes represented by 98 different CpGs between our downregulated gene list (987 genes) and the Illumina Assay. To estimate if the 98 overlapping CpG sites are methylated differently, we performed univariate t-tests for each gene by BRB Array Tools. Statistically significant difference was considered when the P value was less than 0.05 below the 0.1 Benjamini and Hochberg FDR.

### Integration of Gene Expression, Copy Number Variation and Promoter Methylation Datasets

To study the relationship between DNA copy number gains/losses and mRNA levels, we exported the median of the replicate probe log_2_ ratios and the expression values corresponding to the same genomic region for determining Pearson’s correlation. The genelist generated through this analysis was imported to the Nexus 5.1 package. We assessed the genomic locations that exhibited significant copy number losses and concentrations of downregulated genes simultaneously. The statistics-generated P value was based on the number of deregulated transcripts located in significantly deleted genomic regions after multiple testing correction.

To verify the effects of cis- regulatory copy number elements on gene expression and to assess trans- acting copy number alterations, we used the ‘lol’ R package applying cross-validation as optimizer [37]–[39]. Detailed description of this approach is available under the Text S2.

To assess relation between gene expression and promoter methylation patterns, we applied Pearson’s correlation for each of the 98 CpG sites corresponding to the 45 overlapping genes between the datasets.

### Pathway Analysis of the Gene Expression Data

Functional characterisation of differentially expressed genes (p<0.05) that showed differences of greater than 2-fold between the melanoma groups (ulcerated melanomas versus melanomas without an ulcerated surface) was performed using Ingenuity Pathways Analysis software (IPA, Ingenuity Systems: www.ingenuity.com) and the Database for Annotation Visualisation and Integrated Discovery (DAVID, http://david.abcc.ncifcrf.gov) Gene Functional Classification Tool. Datasets containing gene identifiers (Affymetrix ID) and the corresponding expression values were evaluated. Each gene identifier was mapped to its corresponding gene object in the Ingenuity Pathways Knowledge Base (IPKB). Functional analysis of the genes was based on the collection of data summarised and stored in the IPKB database. Genes identified using the IPA program were categorised according to location, cellular components and their reported biochemical, biological, and molecular functions.

### Validation of the Gene Expression Microarray Results by QPCR

The expression status of selected genes was performed using quantitative real-time PCR in all melanoma samples with the ABI Prism® 7900HT Sequence Detection System (Applied Biosystems, Carlsbad, California, USA). Reverse transcription (RT) was carried out on total RNA (600 ng) using the High Capacity cDNA Archive Kit, according to the protocol of the supplier (Applied Biosystems, Carlsbad, California, USA). A TaqMan Low Density Array with pre-designed TaqMan® Gene Expression Assays (Applied Biosystems, Carlsbad, California, USA) was used to perform qPCR for 17 genes (Table S1).

Selection of genes covered several genes from Table 2. according to the following IPA functions: Hair and Skin Development and Function, Organ Development, Dermatological Diseases and Conditions (ITGB4, SDC1, FGFR2, FGFR3); Cancer, Cellular Movement, Cellular Growth and Proliferation (KLF4, ARHGEF, EGFR); Cellular Development, Hematological System Development and Function, Hair and Skin Development and Function (KLK11, KLK5, JUP, EHF); Cancer, Cell Cycle, Reproductive System Disease (PERP). Additionally, 5 genes (CTSL, EPSL8L1, EPSL8L2, IL18, RAB25) were randomly selected from Table S1 comprising 987 genes that had been proved to be downregulated in ulcerated melanomas by microarray technique.

**Table 2:** Networks and pathways of downregulated genes associated with ulcerated melanoma surface.

Gene symbol	Main function (IPA)^a^	Signaling pathways (KEGG)^b^
ANK3, CLCA2, DSC1, DSG1, DSG3, DSP, DST, EVPL, FGFR2, FGFR3, FLG, IRF6, ITGB4, IVL, JUP, KLK7,KRT5, KRT10, MAPK13, NTRK2, PKP1, POU2F3,PPL, S100A7, SDC1, SERPINB5, SFN, SULF1, TP73L	Hair and Skin Development and Function,Organ Development, DermatologicalDiseases and Conditions	*Olfactory transduction*: CLCA2; *Cell Communication:* DSC1, DSG, DSG3, ITGB4, KRT5, KRT10; *MAPK signaling pathway:* FGFR2, FGFR3, MAPK13; *Cell adhesion molecules:* SDC1
ALDH2, ARHGEF4, ASS1, AURKA, BCL11B, CA2,EGFR, ETS2, GATA3, GJA1, KLF4, KLF5, LAMA3,LGALS7, MAF, PTGS1, SCNN1A, SERPINB3,SLC2A1, SPP1, THBD, TPSAB1, WNT5A	Cancer, Cellular Movement, Cellular Growthand Proliferation	*Metabolism*: ALDH2, ASS1, CA2, PTGS1; *Regulation of cytoskeleton:* ARHGEF4; *Cell cycle:* AURKA; *Adherens junction:* EGFR; *Axon guidance :* ETS2; *Cell Communication :* GJA1, LAMA3, SPP1; *Hemostasis:* THBD; *Wnt Signaling pathway:* WNT5A
AHNAK, ANKRD57, CA12, CD24, CXADR, DEFB1,EPHB6, EPPK1, EVA1, FGFR2, FZD10, GABRE,GJB3, GRHL2, PRSS, SDC1, SERPINB5, SPINK5	Cancer, Tumor Morphology, CellularMovement	*Metabolism* : CA12; *Hematopoietic cell lineage*: CD207; *Axon guidance*: EPHB6; *MAPK signaling pathway:* FGFR2; *Wnt signaling pathway:* FZD10; *Neuroactive ligand-receptor interaction:* GABRE, PRSS3; *Cell Communication:* GJB3; *Cell adhesion:* SDC1
BICD2, C20ORF42, CD207, CDKN1C, COL4A6,COL7A1, CST6, CSTA, FCER1A, KLK11, KRT1,LAD1, LY6D, PDZK1IP1, SPINT2, SPRR1A, ST14	Cancer, Cellular Growth and Proliferation,Hair and Skin Developmentand Function	*Cell Communication*: COLA7A1, KRT1
ABLIM1, AKR1B10, CENTD1, CXCL14, DSC3, EFS,F2RL1, FGF2, GJA1, IMPA2, KLK8, KRT15, LTB4R,MAP7, RORA, VSNL1	Cell-To-Cell Signaling and Interaction,Cellular Growth and Proliferation,Cancer	*Axon guidance*: ABLIM1; *Metabolism*: AKR1B10, IMPA2; *Cytokine-cytokine receptor interaction* : CXCL14; *Cell Communication*: DSC3, GJA1, LFRT15; *MAPK signaling pathway*: FGFR2; *Neuroactive ligand-receptor interaction*: F2RL1, LTB4R
AKR1C1, AKR1C2, AQP3, BCL11A, CKMT1B,CTNNBIP1, EHF, GNA15, IGF1, JUP, LOR, NMU,PKP3, SCEL, SPRR1B, TACSTD2	Cellular Development, HematologicalSystem Development and Function, Hairand Skin Development and Function	*Metabolism:* AKR1C1, AKR1C2, CTKMT1B; *Wnt signaling pathway:* CTNNBIP1; *Calcium signaling pathway:* GNA15
AIM1, CLTB, CPA3, DGKA, FAT2, KRT15, LYPD3,MYO6, PAK6, PERP, PTGS1,TUBB4	Cancer, Cell Cycle, ReproductiveSystem Disease	*Huntington's disease*: CLTB; *Cell Communication*: KRT15; *Metabolism*: DGKA, PTGS1; *Focal adhesion*: PAK6; *Gap junction*: TUBB4
CALML3, CALML5, CCRL1, PRSS8, SDC1, SPP1,THBD	Cellular Movement, Cell-To-Cell Signalingand Interaction, Hematological SystemDevelopment and Function	*Calcium signaling pathway:* CALML3; *Cell adhesion molecules:* SDC1; *Cell Communication:* SPP1; *Hemostasis:* THBD

a IPA: Ingenuity Pathways Analysis;

b KEGG Kyoto Encyclopedia of Genes and Genomes.

The house keeping genes β-actin (Hs99999903_m1) and glyceraldehyde-3-phosphate dehydrogenase (GAPDH; Hs99999905_m1) were used as controls for accurate normalisation of the gene expression data [40]. Pearson’s correlation was used to evaluate the strengths of the relationships between the PCR and the microarray expression data. The Student’s *t*-test was performed to validate statistically significant differences among the predefined sample groups, and the data were considered significant at p<0.05.

## Results

### Gene Expression Alterations of Primary Melanomas

Affymetrix oligonucleotide microarrays were used to investigate the gene expression patterns in 36 primary melanomas. The follow-up period of the patients was 5 years. The clinicopathological parameters of the samples are summarised in Table 1.

**Figure 1:**
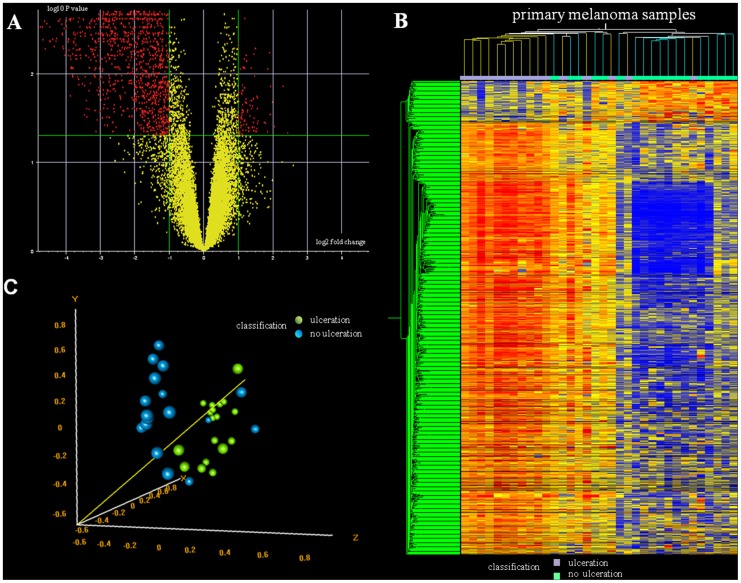
Gene expression patterns of primary melanomas. A: The Volcano plot displays the relationship between fold changes and statistical significance: the x-axis visualizes the log_2_ values of the fold change while the y-axis belongs to the log_10_ values of significance. The green line of the x- and y-axes indicate greater than 2-fold change between ulcerated and non-ulcerated samples and P value cut off 0.001 revealed by univariate t-tests for each gene. The Benjamini and Hochberg false discovery rate was applied as multiple test correction. **B:** Unsupervised hierarchical clustering of the 1080 genes which expressed differentially among ulcerated and non ulcerated melanomas. Samples are displayed vertically and genes are displayed horizontally. Annotation track marks ulcerated samples with violet and non-ulcerated with green colour. The colour in each cell of the table represents the median-adjusted expression value of each gene. Yellow indicates increased expression relative to the median, while blue represents decreased expression. **C:** Principal Component Analysis for distinction of ulcerated (green dots) and non-ulcerated (blue dots) samples based on the 1080 differentially expressed genes. The analysis revealed that according to the first 3 components which covered the 59% of the total variance the 2 groups were significantly different (p<0.001).

After the normalisation and quality control steps were performed for the microarray data, 25,886 probe sets were used in further analyses. Univariate t- tests were performed on log-transformed expression values of the probes to determine differentially expressed genes (P value cut off 0.001, greater than 2-fold change), among all the predefined clinical groups (Table 1). The analysis resulted in 1,080 genes that expressed differentially between ulcerated and non-ulcerated melanomas with FDR false discovery rate of less than 5%. Our results are visualized by Volcano plot (Figure 1 A). The majority of the differentially expressed genes were downregulated (987 genes, Table S1), whereas only 93 genes (Table S2) were significantly upregulated in ulcerated specimens compared to non-ulcerated melanomas. Alternatively, hierarchical clustering of the 1,080 differentially expressed genes distinguished also the primary melanoma subgroups, and melanomas with a tumour surface ulceration were clustered mainly together (Figure 1B). To measure how ulcerated and non-ulcerated groups can be distinguished according to the 1080 differentially expressed genes, we performed Principal Component Analysis (PCA). Based on the global test of clustering (p<0.001), the first 3 components covered the 59% of the total variance.

Using univariate t-tests and PCA for further verification we were unable to describe differentially expressed genes among other sample groups categorized by histologycal subtype, Breslow thickness, Clark level and metastasis formation.

For survival analysis we established Penalized Cox Regression model that revealed no relationship between patients’ survival and gene regulation when we considered patients’ age, gender and other clinical cofounders, including survival.

### Functional Categorisation of Differentially Expressed Genes

The functional annotation of upregulated genes was performed using the web-accessible programs of the Database for Annotation Visualisation and Integrated Discovery (DAVID). Of the 93 overexpressed genes, 79 could be identified by DAVID, and 23 of these genes belonged to the well-known KEGG pathways (Kyoto Encyclopaedia of Genes and Genomes). These data are summarised in Table 3. The overexpressed genes in the ulcerated melanoma cluster included many genes with reported functional roles in cell cycle regulation and proliferation, such as cell division cycle 25a (CDC25a) and MAPKK12. Out of the 93 genes, the expression of the osteopontin (SPP1) gene showed the greatest difference between the two melanoma subgroups (fold change value: 6). Most of the genes from the KEGG pathways were linked to cell adhesion/extracellular matrix (ECM) interactions. These genes include osteopontin, beta-catenin, syndecan 3, Shc family member 4 and v-akt murine thymoma viral oncogene homolog 3, which affects the MAPK and ErbB signalling pathways. Two additional genes (CCR1 and EPOR) were involved in cytokine-cytokine receptor interactions. Increased expression was also detected of genes in different (e.g., pentose, fructose, inositol) metabolism pathways.

**Table 3:** Functional annotation of the upregulated genes in ulcerated melanomas.

Affymetrix ID	Gene Name	KEGG Pathway^a^
209875_s_at	secreted phosphoprotein 1 (osteopontin)	Cell Communication, Focal adhesion, ECM-receptor interaction, Toll-like receptor signaling pathway
223679_at	catenin (cadherin-associated protein), beta	Wnt signaling pathway, Focal adhesion, Adherens junction, Tight junction
202898_at	syndecan 3 (n-syndecan)	ECM-receptor interaction, Cell adhesion molecules (CAMs)
242879_x_at	v-akt murine thymoma viral oncogene homolog 3	MAPK signaling pathway, ErbB signaling pathway, mTOR signaling pathway, Apoptosis, VEGF signaling pathway, Focal adhesion, Tight junction, Toll-like receptor signaling pathway, Jak-STAT signaling pathway
235238_at, 230538_at	shc (src homology 2 domain containing) family, member 4	ErbB signaling pathway, Focal adhesion
205099_s_at	chemokine (c-c motif) receptor 1	Cytokine-cytokine receptor interaction
215054_at	erythropoietin receptor	Cytokine-cytokine receptor interaction, Jak-STAT signaling pathway
204015_s_at	dual specificity phosphatase 4	MAPK signaling pathway
205447_s_at	mitogen-activated protein kinase kinase kinase 12	MAPK signaling pathway
1555772_a_at	cell division cycle 25a	Cell cycle
209435_s_at	rho/rac guanine nucleotide exchange factor (gef) 2	Cell cycle
203651_at, 240859_at, 1554638_at	zinc finger, fyve domain containing 16	TGF-beta signaling pathway
221489_s_at	sprouty homolog 4	Jak-STAT signaling pathway
224341_x_at	toll-like receptor 4	Toll-like receptor signaling pathway
206617_s_at	renin binding protein	Aminosugars metabolism
204271_s_at	endothelin receptor type b	Calcium signaling pathway, Melanogenesis
205119_s_at	formyl peptide receptor 1	Neuroactive ligand-receptor interaction
212884_x_at	apolipoprotein e	Neurodegenerative Diseases
201660_at,201661_s_at	acyl-coa synthetase long-chain family member 3	PPAR signaling pathway
204044_at	quinolinate phosphoribosyltransferase	Nicotinate and nicotinamide metabolism
201272_at	aldo-keto reductase family 1, member B1	Pentose and glucuronate interconversions, Fructose and mannose metabolism, Galactose metabolism
222240_s_at	myo-inositol 1-phosphate synthase a1	Inositol phosphate metabolism
203217_s_at	st3 beta-galactoside alpha-2,3-sialyltransferase 5	Glycosphingolipid biosynthesis

a KEGG: Kyoto Encyclopedia of Genes and Genomes.

Detailed analysis using Ingenuity Pathway Analysis, GeneGo software and the DAVID program revealed networks, pathways and interactions that were enriched for genes that were downregulated in melanomas with an ulcerated surface (987 genes). According to the functional role of the genes, 26 networks were identified via IPA analysis. These networks are related to hair and skin development and functions, dermatological diseases and conditions, cellular development, cellular growth and proliferation, cardiovascular system development and function, cell morphology, cellular assembly and organisation, and cell-to-cell signalling and interactions (Table 2). For example, the “Cancer, Cellular Movement, Cellular Growth and Proliferation” network encompasses EGFR, FGFR2 and other key genes, such as AURKA, a cell cycle checkpoint mediator, and SPP1, LAMA3 and GJA1, which play a role in cell communication.

In the analysed melanomas, the “Cell adhesion, Cadherin” network was significantly enriched for genes for which decreased expression is correlated with ulceration (p<0.0001). This network involves genes playing a significant role in the Wnt (WNT16, WNT3, WNT4, WNT5a), beta-catenin, EGFR, FZD10 and p21 signalling pathways (Figure S1). Interestingly, the members of the Wnt family that have been reported to be overexpressed in the majority of melanomas were overexpressed only in one subset of our melanoma samples. This analysis also revealed that the expression levels of distinct components of the “Junctional Mechanism Regulation Pathway” (i.e., ZO-2, OCLN, GJA1, CLDN1, DSP, JUP) were differentially expressed in the analysed tumours (Figure S2) ZO-2 (TJP2) is a tight junction plaque adapter for the recruitment of cytosolic molecules implicated as integral membrane proteins involved in cell signalling, and Occludin (OCLN) and Claudin 1 (CLNDN1) serve as linkers of the actin cytoskeleton to the plasma membrane and contribute to cytoskeleton organisation, small GTPase-mediated signal transduction, and cellular component organisation. Downregulation of laminin 5, collagen IV and ezrin is related to integrin-mediated cell-matrix adhesion, playing a key role in cell surface structure adhesion, migration and organisation. Compared to melanomas without ulceration, ulcerated melanomas were characterised by downregulation of members of the kallikrein serine protease family (i.e., KLK7, KLK5, KLK11), which are related to angiogenesis and the degradation of extracellular matrix components. The top five identified networks that were affected in tumours with ulcerated surfaces were centred on key genes involved in the p53, Nf-kappaB, WNT/beta-catenin pathways.

### Validation of Expression Microarray Results via Quantitative RT-PCR

The microarray results were validated by performing qRT-PCR measurements of the 17 downregulated genes. Each of the 36 tumour samples included in the microarray analysis was tested using a qPCR based TaqMan Low Density Array.

**Figure 2:**
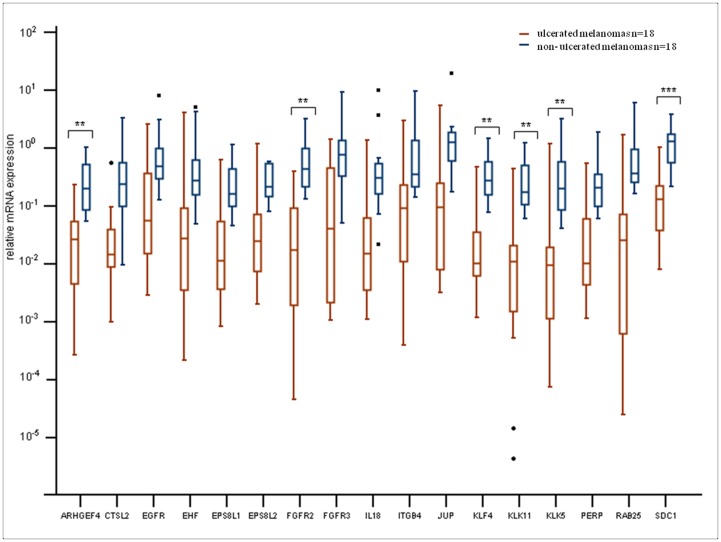
Quantitative RT-PCR validation of the 17 selected genes from the list of 987 downregulated transcripts. The boxplots represent mRNA expressions of ulcerated (in red colour) versus non-ulcerated (in blue colour) primary melanomas. Gene expression differences among the groups were analysed using the Student’s t-tests (**p<0.01, ***p<0.001.

For all of the 17 genes, the expression levels differed between melanomas with or without ulceration (more than a 2-fold change, p<0.05). These data showed a strong correlation with the microarray results. Six of the genes displayed a highly significant (p<0.01) expression difference between the melanoma groups (Figure 2). Two of the genes (KLK11 and KLK5) belong to the protein-degrading kallikrein family. The KLF4 transcription factor showed decreased expression (by PCR as well) and is capable of both activating and repressing genes involved in cell-cycle regulation and differentiation. Furthermore, fibroblast growth factor receptor 2 (FGFR2); ARHGEF4, which acts as a guanine nucleotide exchange factor; and SDC1, a member of the syndecan proteoglycan family, were also expressed at significantly lower levels in ulcerated tumours. These genes are part of the “Cancer, Cellular movement, Cellular Growth and Proliferation” network and the “Hair and Skin Development and Function” network.

### Comparison of Gene Expression Changes to Copy Number Alterations and DNA Methylation Patterns in Primary Melanomas

We performed a detailed aCGH analysis on 17 melanomas and collected integrated array CGH and gene expression data from the same tumour to characterise genetic alterations that might be associated with gene expression changes.

A genome-wide aCGH profiling analysis revealed numerous copy number alterations in the melanoma genome. Ulcerated melanomas displayed gains at 3p, 6p, 7p, 7q, 8q, 11p, 11q, 15q, 16p, 17q, 18q and 22q, with the smallest regions detected at 3p21.31-p21.2, 6p21.2-p21.1, 7p14.3, 7q36.2, 8q24.3, 11p15.5-p15.4, 11q13.3, 15q22.33-q23, 15q24.2, 16p13.3, 17q25.3, 18q21.1 and 22q12.3. Losses were found at 4q, 7q, 9q, 11p, 14q, 15p, 15q, 17q, 20q, 21p with the smallest regions observed at 4q28.3, 7q11.1, 9q12, 9q13, 11p11.2-p11.12, 14q11.1-q11.2, 15p11.1- q11.1, 17q21.31, 20q11.1, 21p11.2 (p<0.05). Table 4 summarises the most frequent and statistically significant copy number losses associated with melanoma ulceration.

**Table 4:** Distribution of the most frequent and statistically significant copy number losses in ulcerated surfaced melanomas.

Cytoband Location	Region Length (bp)	Frequency^a^ (%)	P value^b^	Distribution of known CNV overlap^c^ (%)
4q28.3	470255	29	0.020	27
7q11.1	468074	29	<0.001	94
9q12	179861	53	0.005	0
9q13	379778	53	0.005	72
11p11.2-p11.12	945190	35	<0.001	5
15p11.1-q11.1	682823	29	<0.001	0
15q11.1	463101	29	<0.001	41
17q21.31	160872	29	0.007	0
20q11.1	691516	35	<0.001	0
20p11.2	319491	76	<0.001	0

a Copy number loss frequency indicates the proportion of samples that exhibit the given genomic loss.

b P value has been determined by a multiple corrected Fisher’s exact test.

c CNV overlap is the occurrence of copy number events that exist in healthy donors according to the Copy Number Project database (Wellcome Trust Sanger Institute).

**Figure 3:**
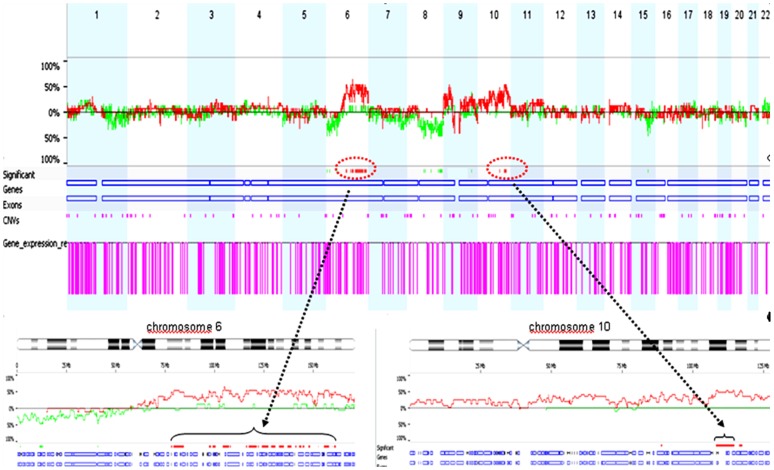
Correlation of copy number alterations (CNAs) and gene expression. Chromosomes 6 and 10 shows significantly different copy number losses that are highly correlated with gene downregulation based on comparison of CNAs in the ulcerated vs. non-ulcerated tumour subgroups. The differences in CNAs (red indicates CNA losses, and green indicates CNA gains) were obtained by subtracting the alterations in the ulcerated group (above the baseline) from the alterations in the non-ulcerated group (below the baseline) using Fisher’s exact test corrected for multiple testing. A multiple corrected *t*-test was performed to determine the number of downregulated genes located in significantly deleted genomic regions. Five narrowed deleted regions were found on chromosome 6 and one deleted region was detected on chromosome 10q that was also downregulated.

To determine the influence of copy number aberrations on the gene expression changes in primary melanomas, we sought to identify the genes whose expression was significantly correlated with copy number changes. By correlating the intensity of the aCGH ratios (median log2) with the expression levels of previously identified downregulated genes (987 genes), we identified 150 genes whose expression was significantly and positively correlated with copy number changes in ulcerated melanomas (p<0.05, Table S3). The gene list generated through this analysis was significantly enriched in genes mapping to chromosomes 6q and 10q (Table 5). Figure 3 shows the frequency distribution of copy number losses in ulcerated (red line) and non-ulcerated (green line) melanomas. We found more narrow regions that were deleted on the long arm of chromosome 6 (6q14.1-q14.2, 6q16.3-q21, 6q22.31-q22.32, 6q23.3 and 6q24.2), consisting of 9 deregulated genes (ELOV4, ME1, TPBG, AIM1, TPD52L1, IL20RA, HEPB2, PERP and UTRN). A significant association between genomic losses and the downregulation of genes on chromosome 10 was only observed for one gene (ABLIM1, localised to 10q25).

**Table 5:** Genomic regions enriched with downregulated genes affected by significant genomic losses in ulcerated melanomas.

Cytoband Location	Region Length (bp)	CNV Loss Frequencyin UlceratedSamples^a^ (%)	CNV Loss Frequencyin Non-ulcerated Samples^b^ (%)	P value^c^	Known CNVOverlap^d^ (%)	Downregulated Genes
6q14	2239920	47	0	0.023	0	ELOVL4
						ME1
						TPBG
6q21	2010714	53	0	0.009	2	AIM1
6q22.31-q22.32	2121683	53	0	0.009	2	TPD52L1
6q23.3	1160478	53	0	0.009	0	IL20RA
						HEPB2
						PERP
6q24.2	118530	47	0	0.023	0	UTRN
10q25.3	368863	47	0	0.023	8	ABLIM1

a Copy number loss frequency in ulcerated samples indicates the proportion of ulcerated melanomas exhibiting the particular genomic loss.

b Copy number loss frequency in non-ulcerated samples indicates the proportion of melanomas exhibiting the particular genomic loss.

c P value has been determined by a multiple corrected *t*-test considering the numbers of downregulated transcripts located on significantly deleted genomic regions.

d Known CNV overlap is the occurrence of copy number events existing in healthy donors according to the Copy Number Project database (Wellcome Trust Sanger Institute).

**Figure 4:**
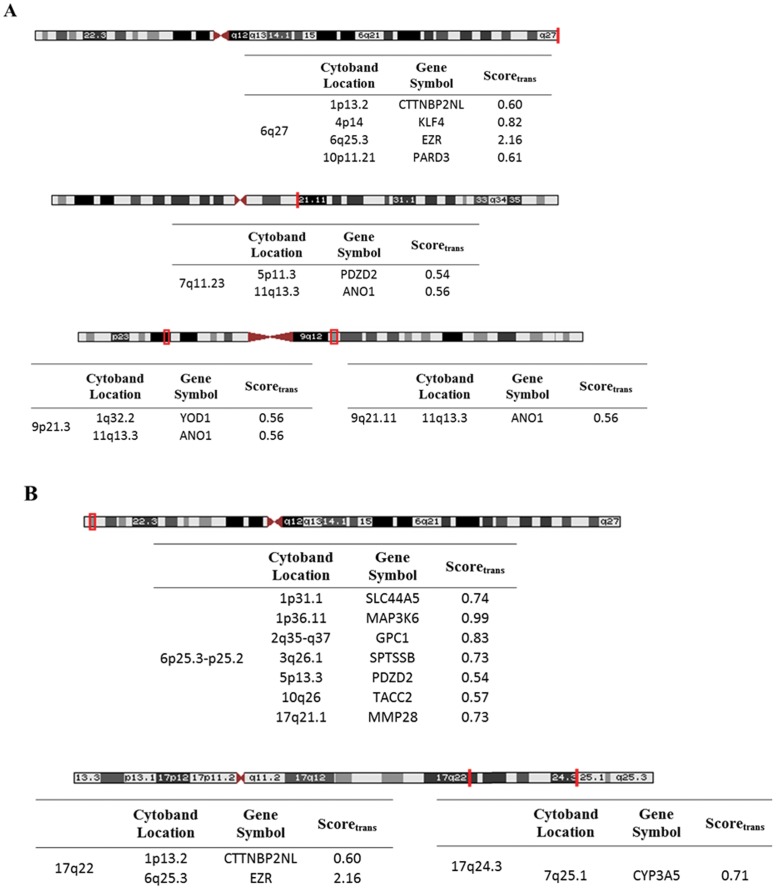
Trans-acting copy number alterations. Lasso regression was performed on the previously identified 1080 differentially expressed genes to assess trans- acting copy number alterations on mRNA expression. Cytoband locations were mapped by UCSC Genome Browser and the tables below represent the genes whose expression can possibly be affected by CN losses (**A**) and gains (**B**) at trans-acting CN elements. The score_trans_ represents the strength of the relationship between trans-acting elements and mRNA expression.

Lasso regression, performed by lol R-package, identified both copy number gains (Figure 4 B) and losses (Figure 4 A) as top scoring (Score_trans_ ≥0.5) trans- acting somatic DNA aberrations accompanied by transcriptomic alterations. Interestingly, even trans- acting copy number gains were accompanied by transcriptomic silencing, there was no association between gains and gene upregulation revealed. Three distinct copy number losses (9p21.3, 9q21.11 and 7q11.23) were related to the downregulation of Anoctamin1 (Ano1), whereas Ezrin (EZR) deregulation was probably caused by the copy number loss of 6q27 (Figure 4 A) and the copy number gain of 17q22 (Figure 4 B).Cis- affecting copy number losses given in Table 5 were verified by Lasso- regression as we revealed Score_cis_ values more than 0.5 for each region.

**Figure 5:**
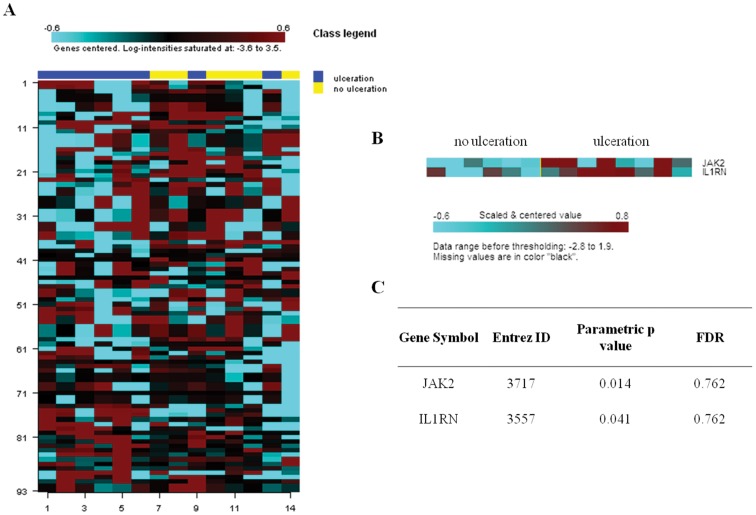
Relationship between DNA methylation and melanoma ulceration. Clustered heatmap, performed to demonstrate promoter methylation patterns, shows lack of differences between ulcerated and non- ulcerated sample groups. The heatmap is based on the univariate t-tests performed for each sites (specific for 45 independent genes) that overlapped with the gene expression results. Two genes (IL1RN and JAK2) demonstrated increased methylation for the ulcerated sample group, however, significant differences did not remain after adjustment for multiple comparison.

To define the influence of the epigenetic alterations on melanoma ulceration, we estimated if the 98 overlapping CpG sites were methylated differently by univariate t- tests for each genes. Figure 5A plots a clustered heat map showing the lack of differences between the sample groups with an exception of JAK2 and IL1RN genes (Figure 5B), however, significant differences did not remained after adjustment of multiple comparison (Figure 5C).

Furthermore, we correlated the gene expression data with the observed epigenetic changes (Illumina Golden Gate Cancer Panel 1) in the same tumour. Of the 987 downregulated genes identified using the Affymetrix microarray, we found 45 common genes represented by 98 different CpGs on the methylation array platform. A strong or medium inverse correlation (a medium inverse correlation was assessed if r≤−0.30 and a strong inverse correlation if r≤−0.50 by Pearson’s correlation) between gene expression and methylation levels was detected in the case of 11 genes corresponding to 17 different CpG sites. Detailed list of these genes are shown in Table 6.

**Table 6:** Genomic regions enriched with downregulated genes affected by promoter hypermethylation.

Gene Symbol	Cytogenetic Location	Probe ID	Pearson’s *r*
EPHB3	3q27.1	EPHB3_E0_F	−0.56
		EPHB3_P569_R	−0.39
FGFR3	4p16.3	FGFR3_E297_R	−0.44
		FGFR3_P1152_R	−0.11
ITGA2	5q11.1	ITGA2_E120_F	−0.60
		ITGA2_P26_R	−0.40
DST	6p12.1	DST_E31_F	−0.38
		DST_P262_R	−0.41
EPHB6	7q34	EPHB6_P827_R	−0.34
PTGS1	9q33.3	PTGS1_E80_F	−0.45
		PTGS1_P2_F	−0.25
FGFR2	10q26.13	FGFR2_P266_R	−0.37
		FGFR2_P460_R	−0.29
CDH13	16q23.3	CDH13_E102_F	−0.50
		CDH13_P88_F	−0.37
JAG1	20p12.2	JAG1_P66_F	−0.47
TIAM1	21q22.11	TIAM1_P117_F	−0.34
		TIAM1_P188_R	−0.20
ETS2	21q22.2	ETS2_P684_F	−0.69
		ETS2_P835_F	−0.33

## Discussion

Malignant melanoma develops from the malignant transformation of melanocytes, the pigment-producing cells in the basal epidermal layer of human skin, and is one of the most aggressive human cancers. Melanoma arises from the accumulation of different alterations in genes that are critical for cell proliferation, differentiation, and cell death [41], [42]. To develop treatments for advanced melanoma and to increase survival in metastatic melanoma patients, it is important to characterise the genetic and gene expression changes leading to each progressive step of the disease.

During the last decade, microarrays have become the technology of choice for the selection of the genes responsible for the behaviour of malignant lesions. Previously published microarray results have shown highly different and frequently contradictory “melanoma signatures” containing many genes whose functional roles in melanoma progression have not been well characterised. Furthermore, the gene sets found in different microarray studies overlap poorly with each other [20], [21], [43], [44]. In this study, we defined the gene expression profiles in 36 primary melanoma tissues and correlated these data with DNA copy number alterations and epigenetic changes in the selected samples. Using the Affymetrix microarray platform, we identified 1,080 genes with expression value that was significantly associated with the tumour surface ulceration of the primary tumours. The American Joint Committee on Cancer has recently reported that after Breslow thickness, the presence or absence of ulceration is the second most powerful independent predictor of survival for melanoma patients [35], [45]. While the molecular background of melanoma ulceration is still unclear, according to the most recent study involved 4,661 patients, not only the presence but the extent of ulceration could be independent predictors of survival [46]. Furthermore, ulceration was reported to be significant predictive factor for outcome of adjuvant interferon treatment [47]. All of these results prompt further efforts in designing additional studies to obtain better insights into the molecular level of ulceration.

Our functional analysis revealed that the majority of the differentially expressed genes were downregulated (987 genes) and were involved in the p53, Nf-kappaB, and WNT/beta-catenin pathways. In agreement with previous studies, we also observed downregulation of pro-apoptotic genes (e.g., TPL73L and P53AIP1), members of the forkhead box gene family (e.g., FOXQ1) and members of the TNFRSF25 and TNFSF10 protein families [7].

According to the literature, osteopontin expression is closely associated with shortened relapse-free survival and other histological variables, including ulceration, that predict relapse and are associated with metastatic dissemination [48]–[50]. In this study, we have demonstrated that osteopontin is significantly overexpressed in ulcerated melanomas. Our finding agrees with previous studies and supports the assumption that osteopontin expression is a potential biomarker of melanoma progression [48], [50].

For obtaining a better insights into the molecular mechanisms that might be responsible for the aggressive phenotype associated with the observed gene expression signatures of ulcerated melanomas, we extended our study to search for DNA copy number alterations and epigenetic changes that might influence transcription. Array CGH is currently the best tool for searching for non-random DNA copy number alterations in cancer genomes and finding new genes that harbour copy number gains or losses with prognostic relevance [51]–[53]. As a result of improved resolution, there is currently an abundance of CGH data available in both large genomic regions and for uniquely affected genes. However, these studies focused little on elucidating the genomic alterations that contribute to gene regulation. Valseria et al. were the first to highlight SCNA (somatic copy number alteration) genes. The downregulation and upregulation of these genes is accompanied by genomic losses or gains in melanoma [9]. The present study revealed new genes that might give better insight into copy number alteration (CNA)-induced gene regulation. Because the experiments were based on a metastatic cell line, the aforementioned authors failed to find any progression-related clinical effects associated with these SNCA genes.

The main purpose of this part of the study was to give functional relevance to the copy number events by providing a statistically powerful integrated interpretation of gene expression changes and copy number alterations. Based on our aCGH analyses, we found more regions on chromosome 6q and only one region on chromosome 10q that showed significantly different loss of copy numbers between the two clinical subgroups. It is important to note that these regions were enriched for the downregulated transcripts and significantly correlated with our previously defined gene expression results. There are a total of 36 genes in these two regions, among which we identified 10 downregulated transcripts using the Affymetrix microarray. It is important to note that these genes include TPBG, PERP and UTRN, which are involved in cell-cell and cell-matrix adhesion. PERP also functions as a p53-induced apoptosis effector molecule. Furthermore, TPD52L1 participates in apoptosis followed by nuclear fragmentation. According to the literature, IL20RA is a tissue-specific Interleukin Receptor that is highly expressed in normal skin [54]. The remaining 5 genes could not be directly associated with carcinogenesis; however, a detailed, functional analysis is needed to precisely define the role of each of the new genes we have identified. It is worth noting that previous high-resolution aCGH experiments have also provided convincing evidence of the importance of copy number alterations at 6q, as this region often experiences hemizygous deletion, with MYB1 being the only gene specified on 6q23 [53], [55]. These experiments also suggest that copy number alterations in MYB1 are an important discriminator between melanomas and nevi, as validated by FISH in 123 melanomas and 110 nevi. This study supports the assumption that 6q23 can assist in the diagnosis of melanoma. In agreement with previous findings, we further support the idea that the deletion of 6q23 is an important alteration in melanoma progression. However, it should also be pointed out that additional new genes (IL20RA, HEPB2 and PERP) that are located in this region and are downregulated might make significant contributions to melanoma progression. In addition to 6q23, we found a significant association between the tendency towards gene deregulation and DNA sequence deletions at 6q14, 6q16, 6q22, 6q24 and 6q25. Somatic copy number alterations at 10q were characterised in many cases, but particularly attributed to the PTEN tumour suppressor gene [56].

While systematic correlation analysis among the copy number events and the corresponding genes captures cis- effects it is also important to measure trans- acting copy number alterations (DNA aberrations on one gene affect the mRNA expressions of other genes); for the latter purpose we used „lol” R package developed by Yuan et al. [39], which provided also an appropriate method for validating the cis- acting elements.

Regarding the trans- regulatory copy number elements, the top scoring (Score_trans_) alterations included copy number gains and losses, however, both types of alterations were associated with transcriptomic deregulation. The week immunostaning of EZR protein in the majority of melanomas was mentioned by a single study, however, this phenomenon was not associated with ulceration [57]. In our study, we showed that EZR (6q25.3) deregulation was related to the copy number loss of 6q27 and the copy number gain of 17q22 suggesting the trans regulatory effects of these copy number alterations. Frequent loss of 9p21 region is a well characterized copy number aberration in melanomas [58], [59], however, it has not been mentioned as a trans- regulatory element for Anoctamin 1 gene (11q13.3) so far. As our results demonstrated, the transcriptomic regulation of this gene can be possibly disturbed also by copy number loss of 9q21.11 and 7q11.23 regions.

Parallel to identifying genetic alterations via aCGH, we sought to examine our external dataset of methylation patterns of 1,505 CpG sites corresponding to 807 selected, cancer-related genes. Despite the substantial number of candidate genes that have been shown to be methylated in melanoma, only a few genes were identified as contributors to disease progression [60]. The major reason for this might be that most of these studies have been based on melanoma cell lines and have provided ambiguous results. Few direct experiments have focused mainly on elucidating the differences in methylation patterns between melanomas and benign skin lesions. However, these studies have had a greater impact regarding understanding what drives melanocytic lesions to develop into malignant tumours rather than on the progression of tumours [61].

By correlating 45 overlapping genes between the gene expression dataset and the methylation assay, we identified 11 genes exhibited inverse relationships. Two (EPHB3, EPHB6) of the 11 genes are members of the erythropoietin-producing hepatocyte kinase B (EPHB) receptor family. Both of these genes play a suggested role in tumour suppression via regulating cell adhesion and migration. In addition, these genes have downstream effects on several members of the kallikrein family, which we also found to be downregulated in ulcerated melanomas in line with other groups [15]. Based on our data, we assumed that the downregulation of 2 fibroblast growth factor receptors (FGFR2, FGFR3) probably resulted from promoter hypermethylation of the coding genes. In the literature, downregulation of the remaining genes (CDH13, DST, ETS2, JAG1, ITGA2, PTGS1, TIAM1) have not been mentioned due to promoter hypermethylation so far.

Even with no detection of direct relationship of ulceration and promoter methylation, the above mentioned inverse correlations support the claim that similarly to copy number variation, promoter hypermethylation also plays an important role in transcriptomic silencing. Notably, it was suggested in the literature that Knudson's two-hit hypothesis is often achieved through a combination of DNA methylation and copy number alteration to the same gene [62], [63]. Due to limitation of our studies, parallel detection of both types of somatic alterations was not achieved in primary melanomas. Nevertheless, the inverse relation between gene expression and methylation of the corresponding genes, together with global presence of copy number alteration should be taken into consideration, which suggests that somatic aberrations do not act separately but represent different utility in an integrated apparatus that acts together and develops transcriptomic silencing in ulcerated melanomas.

In summary, this study has provided evidence that the gene expression signatures of primary melanomas are suitable for distinguishing patients with poor and favourable prognoses. We have also shown that different patterns of genetic and epigenetic aberrations associated with distinct molecular subtypes of the disease contribute to the specific transcriptomic profiles of these genes. We believe that our systematic correlation of gene regulation, methylation pattern and the DNA copy number alteration data in the same cohort of primary melanomas will be useful for finding new genes and will allow further functional utilisation of those genes. This study has important implications as we continue to develop a better understanding of the metastatic process associated with melanomas, which will allow us to identify specific genes for use as prognostic markers and, possibly, for targeted therapeutic approaches.

## Supporting Information

Figure S1 **Cell adhesion and Cadherin signalling network.** The network was significantly enriched of those genes which decreased expression is correlated with ulceration (p<0.001). Red circles indicate the downregulated genes.
(TIF)

Figure S2 **Junctional Mechanism Regulation Pathway, enriched of those genes which decreased expression is correlated with ulceration (p<0.001).** Red circles indicate the downregulated genes.
(TIF)

Table S1 **Downregulated transcripts (N  = 987) in ulcerated melanomas.**
(DOC)

Table S2 **Upregulated genes (N  = 93) in ulcerated melanomas.**
(DOC)

Table S3 **Genes for which expression significantly positively correlate with copy number changes in ulcerated melanomas.**
(DOC)

Text S1 **High quality total RNA and DNA isolation, quality controls.**
(DOCX)

Text S2 **Evaluation of cis- and trans acting copy number alterations.**
(DOCX)
