# Correction: Integrative Genomics Identifies Gene Signature Associated with Melanoma Ulceration

**DOI:** 10.1371/annotation/27b2c2c3-3b09-4c0c-9e20-8dc0226404f3

**Published:** 2013-04-18

**Authors:** Zsuzsa Rakosy, Szilvia Ecsedi, Reka Toth, Laura Vizkeleti, Hector Herandez-Vargas, Viktoria Lazar, Gabriella Emri, Istvan Szatmari, Zdenko Herceg, Roza Adany, Margit Balazs

In the Author Byline, the name of the fifth author was misspelled.

It should be: Hector Hernandez-Vargas.

The correct Citation is: Rakosy Z, Ecsedi S, Toth R, Vizkeleti L, Hernandez-Vargas H, et al. (2013) Integrative Genomics Identifies Gene Signature Associated with Melanoma Ulceration. PLoS ONE 8(1): e54958. doi:10.1371/journal.pone.0054958

In Table 1, in the second column and the '20-50' row, the correct value is 7. 

