# Supplementary figures and images for: Integrative Genomics Identifies Gene Signature Associated with Melanoma Ulceration
Source: PLoS One. 2013 Jan 30;8(1):e54958. doi: 10.1371/journal.pone.0054958 (PMC3559846; doi:10.1371/journal.pone.0054958)

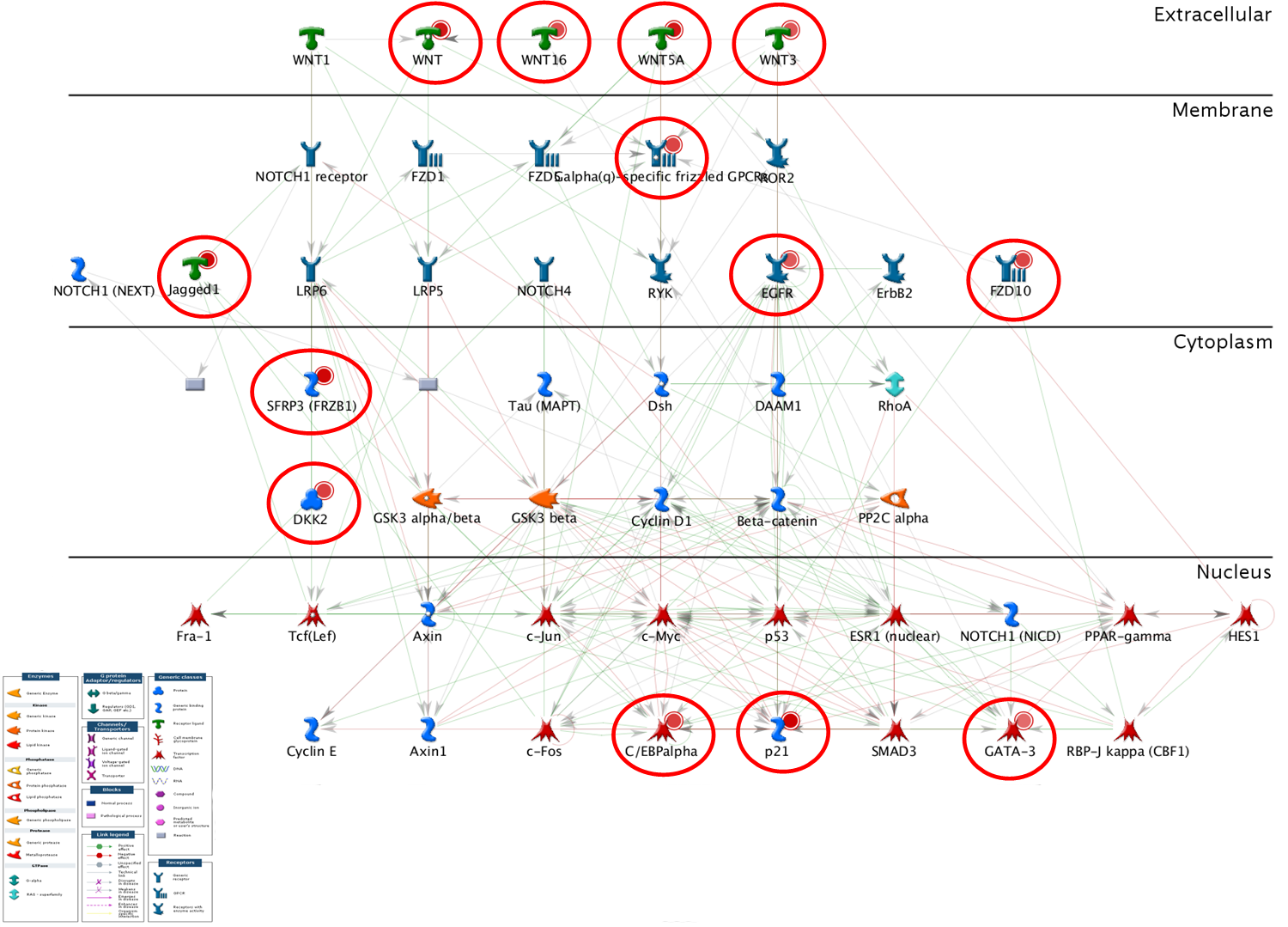

Supplement: Figure S1 — Cell adhesion and Cadherin signalling network. The network was significantly enriched of those genes which decreased expression is correlated with ulceration (p<0.001). Red circles indicate the downregulated genes. (TIF) [file pone.0054958.s001.tif]

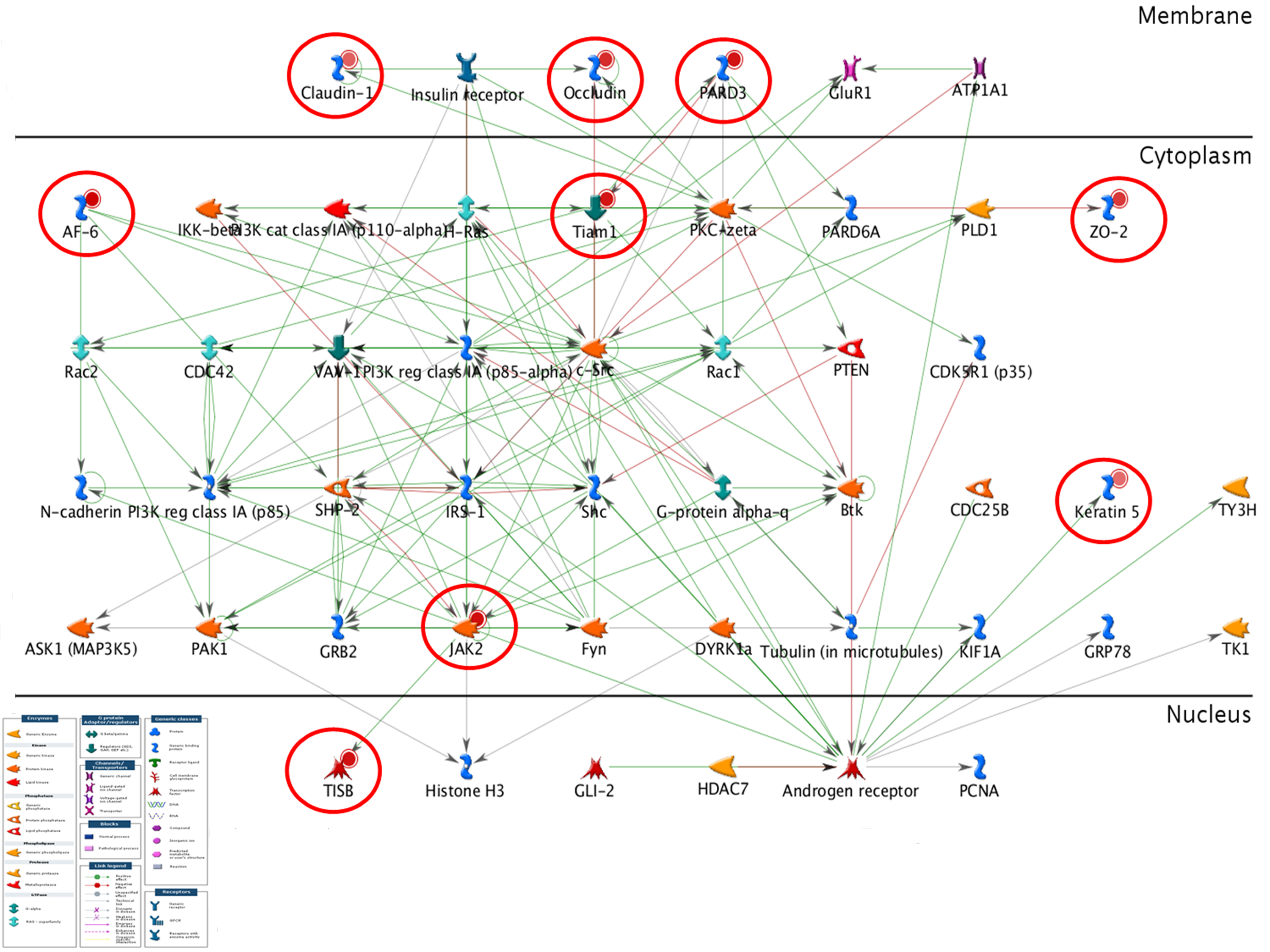

Supplement: Figure S2 — Junctional Mechanism Regulation Pathway, enriched of those genes which decreased expression is correlated with ulceration (p<0.001). Red circles indicate the downregulated genes. (TIF) [file pone.0054958.s002.tif]
