# Supplementary material for: Integrative Genomics Identifies Gene Signature Associated with Melanoma Ulceration
Source: PLoS One. 2013 Jan 30;8(1):e54958. doi: 10.1371/journal.pone.0054958 (PMC3559846; doi:10.1371/journal.pone.0054958)
Supplement: Table S1 — Downregulated transcripts (N = 987) in ulcerated melanomas. (DOC) [file pone.0054958.s003.doc]

**Table S1. Downregulated transcripts (N = 987) in ulcerated melanomas**

| **Probe Set ID** | **Fold Change** | **GenBank ID** | **Entrez Gene ID** | **Gene Symbol** | **Common Name of Transcripts** |
| --- | --- | --- | --- | --- | --- |
| 206193_s_at | 0.0376 | NM_001264 | 1041 | CDSN | S; HTSS; D6S586E |
| 203638_s_at | 0.0387 | NM_022969 | 2263 | FGFR2 | BEK; JWS; CEK3; CFD1; ECT1; KGFR; TK14; TK25; BFR-1; CD332; K-SAM; FGFR2 |
| 221854_at | 0.039 | AI378979 | 5317 | PKP1 | B6P; MGC138829; PKP1 |
| 206192_at | 0.0396 | L20815 | 1041 | CDSN | S; HTSS; D6S586E |
| 207908_at | 0.0408 | NM_000423 | 3849 | KRT2 | KRTE; KRT2A; KRT2E; MGC116967; MGC116968 |
| 207720_at | 0.0415 | NM_000427 | 4014 | LOR | MGC111513 |
| 219995_s_at | 0.0428 | NM_024702 | 79755 | ZNF750 | Zfp750; FLJ13841; MGC125667; MGC125668 |
| 206595_at | 0.0452 | NM_001323 | 1474 | CST6 | CST6 |
| 204734_at | 0.0455 | NM_002275 | 3866 | KRT15 | K15; CK15; K1CO |
| 207324_s_at | 0.0458 | NM_004948 | 1823 | DSC1 | CDHF1; DG2/DG3; DSC1 |
| 220414_at | 0.0478 | NM_017422 | 51806 | CALML5 | CLSP |
| 1569410_at | 0.0497 | BC029685 | 388698 | FLG2 | FLG2 |
| 206421_s_at | 0.0513 | NM_003784 | 8710 | SERPINB7 | MEGSIN; MGC120014; MGC120015; DKFZp686D06190 |
| 206884_s_at | 0.052 | NM_003843 | 8796 | SCEL | FLJ21667; MGC22531; SCEL |
| 211002_s_at | 0.0523 | AF230389 | 23650 | TRIM29 | ATDC; FLJ36085 |
| 216935_at | 0.0534 | AF005082 | 388699 | C1orf46 |  |
| 207710_at | 0.0543 | NM_014357 | 26239 | LCE2B | XP5; LEP10; SPRL1B |
| 206642_at | 0.0556 | NM_001942 | 1828 | DSG1 | DG1; DSG; CDHF4 |
| 204379_s_at | 0.0578 | NM_000142 | 2261 | FGFR3 | ACH; CEK2; JTK4; CD333; HSFGFR3EX |
| 219936_s_at | 0.0584 | NM_023915 | 53836 | GPR87 | GPR95; FKSG78; KPG_002; MGC131898 |
| 206276_at | 0.0585 | NM_003695 | 8581 | LY6D | E48 |
| 205778_at | 0.0588 | NM_005046 | 5650 | KLK7 | SCCE; PRSS6; KLK7 |
| 206400_at | 0.0596 | NM_002307 | 3963 | LGALS7 | GAL7; PIG1; TP53I1 |
| 229290_at | 0.0608 | AI692575 | 92196 | - | LOC92196 |
| 235272_at | 0.0615 | AI814274 | 374897 | SBSN | UNQ698; MGC75533 |
| 1560531_at | 0.0625 | BC031811 | 353132 | LCE1B | LEP2; SPRL2A |
| 206033_s_at | 0.0631 | NM_001941 | 1825 | DSC3 | DSC; DSC1; DSC2; DSC4; CDHF3; HT-CP; DSC3 |
| 202504_at | 0.0632 | NM_012101 | 23650 | TRIM29 | ATDC; FLJ36085 |
| 215704_at | 0.0634 | AL356504 | 2312 | FLG | FLG |
| 1554921_a_at | 0.0641 | BC020726 | 8796 | SCEL | FLJ21667; MGC22531; SCEL |
| 204952_at | 0.0655 | NM_014400 | 27076 | LYPD3 | C4.4A |
| 211597_s_at | 0.0656 | AB059408 | 84525 | HOPX | HOP; OB1; LAGY; Toto; Cameo; NECC1; SMAP31; MGC20820 |
| 209260_at | 0.0657 | BC000329 | 2810 | SFN | YWHAS |
| 214599_at | 0.0665 | NM_005547 | 3713 | IVL | IVL |
| 203453_at | 0.0668 | NM_001038 | 6337 | SCNN1A | ENaCa; SCNEA; SCNN1; FLJ21883; ENaCalpha |
| 1555383_a_at | 0.0676 | BC017500 | 79983 | POF1B | POF; FLJ22792 |
| 244107_at | 0.0684 | AW189097 |  | - |  |
| 206177_s_at | 0.0685 | NM_000045 | 383 | ARG1 | ARG1 |
| 224650_at | 0.0686 | AL117612 | 114569 | MAL2 | MAL2 |
| 238778_at | 0.0688 | AI244661 | 143098 | MPP7 | FLJ32798; RP11-218D6.5 |
| 1554195_a_at | 0.0689 | BC021680 | 389336 | C5orf46 | MGC23985 |
| 224329_s_at | 0.0689 | AB049591 | 84518 | CNFN | PLAC8L2 |
| 39248_at | 0.069 | N74607 | 360 | AQP3 | GIL |
| 203798_s_at | 0.0694 | NM_003385 | 7447 | VSNL1 | HLP3; VILIP; HPCAL3; HUVISL1; VILIP-1 |
| 236534_at | 0.0695 | W69365 | 149428 | BNIPL | PP753; BNIP-S; BNIPL1; BNIPL2; BNIPL-1; BNIPL-2 |
| 220620_at | 0.0699 | NM_019060 | 54544 | CRCT1 | NICE-1; C1orf42 |
| 206643_at | 0.0701 | NM_002108 | 3034 | HAL | HIS; HSTD; histidase |
| 205900_at | 0.0718 | NM_006121 | 3848 | KRT1 | K1; CK1; EHK1; KRT1A |
| 214536_at | 0.0719 | NM_020427 | 57152 | SLURP1 | ARS; MDM; ANUP; ArsB; LY6LS |
| 227238_at | 0.0721 | W93847 | 143662 | MUC15 | MUC15 |
| 230835_at | 0.0723 | W69083 | 388533 | KRTDAP | KDAP; UNQ467; MGC163373 |
| 206032_at | 0.0728 | AI797281 | 1825 | DSC3 | DSC; DSC1; DSC2; DSC4; CDHF3; HT-CP; DSC3 |
| 204855_at | 0.0729 | NM_002639 | 5268 | SERPINB5 | PI5; maspin |
| 232164_s_at | 0.0731 | AL137725 | 83481 | EPPK1 | EPIPL; EPIPL1 |
| 202286_s_at | 0.0735 | J04152 | 4070 | TACSTD2 | M1S1; EGP-1; GA733; TROP2; GA733-1 |
| 209602_s_at | 0.0737 | AI796169 | 2625 | GATA3 | HDR; MGC2346; MGC5199; MGC5445; GATA3 |
| 206165_s_at | 0.0738 | NM_006536 | 9635 | CLCA2 | CaCC |
| 218186_at | 0.0756 | NM_020387 | 57111 | RAB25 | CATX-8 |
| 226926_at | 0.0758 | AA706316 | 93099 | DMKN | UNQ729; ZD52F10 |
| 217528_at | 0.0759 | BF003134 | 9635 | CLCA2 | CaCC |
| 235075_at | 0.0767 | AI813438 | 1830 | DSG3 | PVA; CDHF6; DKFZp686P23184 |
| 220724_at | 0.0773 | NM_025087 | 80157 | CWH43 | FLJ21511 |
| 1553081_at | 0.0775 | NM_080869 | 128488 | WFDC12 | WAP2; SWAM2; C20orf122; dJ211D12.4 |
| 232056_at | 0.0783 | AW470178 | 8796 | SCEL | FLJ21667; MGC22531; SCEL |
| 219756_s_at | 0.0788 | NM_024921 | 79983 | POF1B | POF; FLJ22792 |
| 231722_at | 0.0793 | NM_012114 | 23581 | CASP14 | MGC119078; MGC119079 |
| 232165_at | 0.0793 | AL137725 | 83481 | EPPK1 | EPIPL; EPIPL1 |
| 207109_at | 0.0801 | NM_014352 | 25833 | POU2F3 | OCT11; PLA-1; Epoc-1; Skn-1a; FLJ40063; MGC126698 |
| 1556793_a_at | 0.081 | AK091138 | 128876 | FAM83C | C20orf128; MGC142043; dJ614O4.7 |
| 1555773_at | 0.0811 | AF465766 | 254240 | BPIL2 | BPIL2 |
| 1554593_s_at | 0.0813 | BC028721 | 6511 | SLC1A6 | EAAT4; MGC33092; MGC43671 |
| 207381_at | 0.0816 | NM_001139 | 242 | ALOX12B | 12R-LOX |
| 221470_s_at | 0.0818 | NM_014439 | 27178 | IL1F7 | FIL1; FIL1Z; IL1H4; IL-1F7; IL-1H4; IL1RP1; IL-1RP1; FIL1(ZETA); IL1F7 |
| 216918_s_at | 0.0823 | AL096710 | 667 | DST | BPA; DMH; BP240; BPAG1; MACF2; CATX-15; D6S1101; FLJ46791; KIAA0465; KIAA1470; DKFZp564B2416; DST |
| 235514_at | 0.0828 | AI739528 | 151516 | ASPRV1 | SASP; MUNO; Taps; SASPase |
| 205595_at | 0.0833 | NM_001944 | 1830 | DSG3 | PVA; CDHF6; DKFZp686P23184 |
| 201820_at | 0.0838 | NM_000424 | 3852 | KRT5 | K5; CK5; DDD; EBS2; KRT5A |
| 240420_at | 0.0838 | AA027115 | 344752 | AADACL2 | MGC72001 |
| 204455_at | 0.0841 | NM_001723 | 667 | DST | BPA; DMH; BP240; BPAG1; MACF2; CATX-15; D6S1101; FLJ46791; KIAA0465; KIAA1470; DKFZp564B2416; DST |
| 222242_s_at | 0.0842 | AF243527 | 25818 | KLK5 | SCTE; KLKL2; KLK-L2 |
| 231771_at | 0.0846 | AI694073 | 10804 | GJB6 | ED2; EDH; HED; CX30; DFNA3 |
| 227241_at | 0.0874 | R79759 | 143662 | MUC15 | MUC15 |
| 203074_at | 0.0877 | NM_001630 | 244 | ANXA8 | ANX8; ANXA8L2; FLJ32754; VAC beta |
| 231930_at | 0.0895 | AL359601 | 55531 | ELMOD1 | DKFZp547C176 |
| 211906_s_at | 0.0912 | AB046400 | 6318 | SERPINB4 | PI11; SCCA1; SCCA2; LEUPIN; SCCA-2 |
| 205185_at | 0.0918 | NM_006846 | 11005 | SPINK5 | NS; NETS; LEKTI; LETKI; VAKTI; FLJ21544 |
| 206164_at | 0.0919 | NM_006536 | 9635 | CLCA2 | CaCC |
| 238017_at | 0.0922 | AI440266 | 195814 | SDR16C5 | RDHE2; RDH#2; RDH-E2; FLJ33105 |
| 230323_s_at | 0.0924 | AW242836 | 120224 | TMEM45B | FLJ40787 |
| 235651_at | 0.0927 | AV741130 |  | - |  |
| 229764_at | 0.0935 | AW629527 | 285386 | FAM79B | FLJ41238; FLJ43694; MGC126599; MGC126601 |
| 1552544_at | 0.0939 | BC040857 | 145264 | SERPINA12 | OL-64 |
| 236119_s_at | 0.0947 | AA456642 | 6706 | SPRR2G | SPRR2G |
| 218963_s_at | 0.0949 | NM_015515 | 25984 | KRT23 | K23; CK23; HAIK1; MGC26158; DKFZP434G032 |
| 237120_at | 0.0952 | AI186548 | 374454 | KRT77 | KRT1B; MGC148087 |
| 209720_s_at | 0.0953 | BC005224 | 6317 | SERPINB3 | SCC; T4-A; SCCA1; SCCA-1; HsT1196; SCCA-PD |
| 209800_at | 0.0956 | AF061812 | 3868 | KRT16 | K16; CK16; K1CP; NEPPK; KRT16A |
| 208228_s_at | 0.0958 | M87771 | 2263 | FGFR2 | BEK; JWS; CEK3; CFD1; ECT1; KGFR; TK14; TK25; BFR-1; CD332; K-SAM; FGFR2 |
| 1552487_a_at | 0.0962 | NM_001717 | 646 | BNC1 | BNC; BSN1; HsT19447 |
| 1558687_a_at | 0.0986 | AI288186 |  | - |  |
| 219597_s_at | 0.0987 | NM_017434 | 53905 | DUOX1 | LNOX1; THOX1; NOXEF1; MGC138840; MGC138841; DUOX1 |
| 209719_x_at | 0.0997 | U19556 | 6317 | SERPINB3 | SCC; T4-A; SCCA1; SCCA-1; HsT1196; SCCA-PD |
| 227736_at | 0.0999 | AA553959 | 387695 | C10orf99 | UNQ1833; FLJ21763 |
| 209885_at | 0.1 | BC001338 | 29984 | RHOD | Rho; ARHD; RHOM; RHOHP1 |
| 206605_at | 0.101 | NM_006025 | 8909 | ENDOU | P11; PP11; PRSS26; MGC133268 |
| 205363_at | 0.101 | NM_003986 | 8424 | BBOX1 | BBH; BBOX; G-BBH; gamma-BBH |
| 222484_s_at | 0.103 | AF144103 | 9547 | CXCL14 | KS1; Kec; BMAC; BRAK; NJAC; MIP-2g; SCYB14; MGC10687; bolekine |
| 232202_at | 0.103 | AK024927 |  | - |  |
| 231733_at | 0.103 | NM_021571 | 59082 | CARD18 | ICEBERG |
| 219476_at | 0.103 | NM_024115 | 79098 | C1orf116 | SARG; MGC2742; MGC4309; FLJ36507; DKFZp666H2010 |
| 1553454_at | 0.104 | NM_152364 | 126638 | RPTN | FLJ39117 |
| 1566140_at | 0.104 | AK096707 | 84525 | HOPX | HOP; OB1; LAGY; Toto; Cameo; NECC1; SMAP31; MGC20820 |
| 243871_at | 0.104 | AI083557 |  | - |  |
| 222830_at | 0.106 | BE566136 | 29841 | GRHL1 | MGR; LBP32; LBP-32; TFCP2L2; GRHL1 |
| 243582_at | 0.106 | AW082633 | 153769 | SH3RF2 | RNF158; FLJ23654; MGC90410; MGC149788; MGC149789 |
| 206023_at | 0.107 | NM_006681 | 10874 | NMU | NMU |
| 200606_at | 0.108 | NM_004415 | 1832 | DSP | DPI; DPII; DSP |
| 218002_s_at | 0.108 | NM_004887 | 9547 | CXCL14 | KS1; Kec; BMAC; BRAK; NJAC; MIP-2g; SCYB14; MGC10687; bolekine |
| 215465_at | 0.108 | AL080207 | 26154 | ABCA12 | LI2; ICR2B; FLJ41584; DKFZp434G232; ABCA12 |
| 242204_at | 0.11 | AI242082 | 149708 | WFDC5 | PRG5; WAP1; dJ211D12.5 |
| 228462_at | 0.111 | AI928035 | 153572 | IRX2 | IRX2 |
| 232116_at | 0.111 | AL137763 | 57822 | GRHL3 | SOM; TFCP2L4; MGC46624; GRHL3 |
| 243386_at | 0.111 | AI085338 | 163301 | - |  |
| 220013_at | 0.111 | NM_024794 | 79852 | ABHD9 | FLJ22408; MGC131519 |
| 224328_s_at | 0.112 | AB048288 | 84648 | LCE3D | LEP16; SPRL6A; SPRL6B; MGC126833 |
| 226863_at | 0.112 | AI674565 |  | - |  |
| 39249_at | 0.112 | AB001325 | 360 | AQP3 | GIL |
| 209211_at | 0.112 | AF132818 | 688 | KLF5 | CKLF; IKLF; BTEB2 |
| 231849_at | 0.113 | AL162069 | 144501 | KRT80 | KB20 |
| 219630_at | 0.113 | NM_005764 | 10158 | PDZK1IP1 | DD96; SPAP; MAP17; RP1-18D14.5 |
| 241412_at | 0.113 | AI620677 | 685 | BTC | BTC |
| 205064_at | 0.113 | NM_003125 | 6699 | SPRR1B | SPRR1; GADD33; CORNIFIN; MGC61901 |
| 234700_s_at | 0.113 | AJ131212 | 84659 | RNASE7 | MGC133220 |
| 208650_s_at | 0.114 | BG327863 | 934 | CD24 | CD24A |
| 209863_s_at | 0.114 | AF091627 | 8626 | TP73L | KET; LMS; RHS; p51; p63; EEC3; OFC8; TP63; p73H; p73L; SHFM4; B(p51A); B(p51B) |
| 209772_s_at | 0.114 | X69397 | 934 | CD24 | CD24A |
| 210397_at | 0.115 | U73945 | 1672 | DEFB1 | BD1; HBD1; DEFB-1; DEFB101; MGC51822 |
| 206125_s_at | 0.115 | NM_007196 | 11202 | KLK8 | NP; HNP; NRPN; PRSS19; TADG14; KLK8 |
| 213796_at | 0.115 | AI923984 | 6698 | SPRR1A | SPRK |
| 201015_s_at | 0.115 | NM_021991 | 3728 | JUP | DP3; PDGB; PKGB; CTNNG; DPIII; JUP |
| 228575_at | 0.115 | AL578102 | 53833 | IL20RB | DIRS1; FNDC6; IL-20R2; MGC34923 |
| 1554179_s_at | 0.115 | BC032306 | 66004 | LYNX1 | SLURP2; MGC40364; LYNX1 |
| 220266_s_at | 0.116 | NM_004235 | 9314 | KLF4 | EZF; GKLF |
| 226803_at | 0.117 | AK000049 | 92421 | CHMP4C | Shax3; MGC22825 |
| 33323_r_at | 0.118 | X57348 | 2810 | SFN | YWHAS |
| 205157_s_at | 0.118 | NM_000422 | 3872 | KRT17 | PC; K17; PC2; PCHC1 |
| 1553589_a_at | 0.119 | NM_005764 | 10158 | PDZK1IP1 | DD96; SPAP; MAP17; RP1-18D14.5 |
| 203407_at | 0.12 | NM_002705 | 5493 | PPL | KIAA0568; MGC134872 |
| 204508_s_at | 0.12 | BC001012 | 771 | CA12 | CAXII; FLJ20151; HsT18816; CA12 |
| 225667_s_at | 0.12 | AI601101 | 151354 | FAM84A | NSE1; PP11517; FLJ35392 |
| 220723_s_at | 0.121 | NM_025087 | 80157 | CWH43 | FLJ21511 |
| 236009_at | 0.121 | AI767250 |  | - |  |
| 205627_at | 0.121 | NM_001785 | 978 | CDA | CDD |
| 1555942_a_at | 0.121 | AK091113 |  | - |  |
| 1563900_at | 0.121 | AK055204 | 222584 | FAM83B | C6orf143; FLJ30642; MGC126677; MGC138480 |
| 1552797_s_at | 0.122 | NM_144707 | 150696 | PROM2 | PROM-2; MGC138714 |
| 217521_at | 0.123 | N54942 | 3034 | HAL | HIS; HSTD; histidase |
| 210096_at | 0.123 | J02871 | 1580 | CYP4B1 | P-450HP |
| 209126_x_at | 0.124 | L42612 | 3854 | KRT6B | K6B; PC2; CK6B; KRTL1 |
| 223832_s_at | 0.125 | BC005397 | 84290 | CAPNS2 | MGC12536; MGC14804 |
| 210297_s_at | 0.125 | U22178 | 4477 | MSMB | MSP; PSP; IGBF; MSPB; PN44; PRPS; PSP57; PSP94; PSP-94; MSMB |
| 1553077_at | 0.126 | NM_148897 | 121214 | SDR9C7 | SDR-O; RDHS; SDRO; FLJ16333; MGC126600; MGC126602 |
| 1553213_a_at | 0.126 | NM_173352 | 196374 | KRT78 | K5B; Kb40 |
| 239853_at | 0.126 | AI279514 | 147700 | KLC3 | KLC2; KLCt; KLC2L; KNS2B |
| 217272_s_at | 0.127 | AJ001698 | 5275 | SERPINB13 | HUR7; PI13; headpin; MGC126870 |
| 209792_s_at | 0.127 | BC002710 | 5655 | KLK10 | NES1; PRSSL1; KLK10 |
| 203779_s_at | 0.127 | NM_005797 | 10205 | EVA1 | EVA; MPZL2; EVA1 |
| 210020_x_at | 0.128 | M58026 | 810 | CALML3 | CLP |
| 204942_s_at | 0.129 | NM_000695 | 222 | ALDH3B2 | ALDH8; ALDH3B2 |
| 239381_at | 0.129 | AU155415 | 5650 | KLK7 | SCCE; PRSS6; KLK7 |
| 218796_at | 0.13 | NM_017671 | 55612 | C20orf42 | URP1; KIND1; DTGCU2; FLJ20116; FLJ23423 |
| 216258_s_at | 0.13 | BE148534 | 5275 | SERPINB13 | HUR7; PI13; headpin; MGC126870 |
| 222549_at | 0.13 | AF101051 | 9076 | CLDN1 | CLD1; SEMP1; ILVASC |
| 225645_at | 0.131 | AI763378 | 26298 | EHF | ESE3; ESEJ |
| 205470_s_at | 0.131 | NM_006853 | 11012 | KLK11 | TLSP; PRSS20; MGC33060; KLK11 |
| 205916_at | 0.131 | NM_002963 | 6278 | S100A7 | PSOR1; S100A7c |
| 214651_s_at | 0.132 | U41813 | 3205 | HOXA9 | HOX1; ABD-B; HOX1G; HOX1.7; MGC1934 |
| 239272_at | 0.133 | AI927208 | 79148 | MMP28 | MM28; MMP25; MMP28 |
| 201983_s_at | 0.133 | AW157070 | 1956 | EGFR | ERBB; mENA; ERBB1; EGFR |
| 209604_s_at | 0.133 | BC003070 | 2625 | GATA3 | HDR; MGC2346; MGC5199; MGC5445; GATA3 |
| 204614_at | 0.133 | NM_002575 | 5055 | SERPINB2 | PAI; PAI2; PAI-2; PLANH2; HsT1201 |
| 204679_at | 0.133 | NM_002245 | 3775 | KCNK1 | DPK; HOHO; TWIK1; K2p1.1; TWIK-1 |
| 209309_at | 0.134 | D90427 | 563 | AZGP1 | ZAG; ZA2G |
| 209351_at | 0.134 | BC002690 | 3861 | KRT14 | K14; NFJ; CK14; EBS3; EBS4 |
| 211712_s_at | 0.134 | BC005830 | 8416 | ANXA9 | ANX31 |
| 205709_s_at | 0.134 | NM_001263 | 1040 | CDS1 | CDS |
| 220026_at | 0.134 | NM_012128 | 22802 | CLCA4 | CaCC; CaCC2; MGC142247; MGC142249 |
| 211194_s_at | 0.134 | AB010153 | 8626 | TP73L | KET; LMS; RHS; p51; p63; EEC3; OFC8; TP63; p73H; p73L; SHFM4; B(p51A); B(p51B) |
| 235567_at | 0.134 | AA034012 |  | - |  |
| 206008_at | 0.134 | NM_000359 | 7051 | TGM1 | LI; KTG; LI1; TGK; ICR2; TGASE |
| 211361_s_at | 0.135 | AJ001696 | 5275 | SERPINB13 | HUR7; PI13; headpin; MGC126870 |
| 209872_s_at | 0.135 | BF001503 | 11187 | PKP3 | PKP3 |
| 229103_at | 0.137 | AA463626 | 7473 | WNT3 | INT4; MGC131950; MGC138321; MGC138323 |
| 228708_at | 0.137 | BF438386 |  | - |  |
| 214580_x_at | 0.137 | AL569511 | 3853; 3854; 286887 | - | K6A; K6C; K6D; CK6A; CK6C; CK6D; KRT6C; KRT6D; K6B; PC2; CK6B; KRTL1; KRT6A; KRT6E; MGC102925; MGC163455; MGC163457 |
| 1554252_a_at | 0.138 | BC034500 | 204219 | LASS3 | MGC27091 |
| 209212_s_at | 0.138 | AB030824 | 688 | KLF5 | CKLF; IKLF; BTEB2 |
| 204971_at | 0.138 | NM_005213 | 1475 | CSTA | STF1; STFA |
| 206043_s_at | 0.14 | NM_014861 | 9914 | ATP2C2 | SPCA2; KIAA0703; DKFZp686H22230 |
| 206166_s_at | 0.14 | AF043977 | 9635 | CLCA2 | CaCC |
| 60474_at | 0.141 | AA469071 | 55612 | C20orf42 | URP1; KIND1; DTGCU2; FLJ20116; FLJ23423 |
| 202890_at | 0.141 | AW242297 | 9053 | MAP7 | EMAP115; E-MAP-115 |
| 219115_s_at | 0.141 | NM_014432 | 53832 | IL20RA | IL-20R1; ZCYTOR7; FLJ40993 |
| 236266_at | 0.141 | BG149557 |  | - |  |
| 266_s_at | 0.142 | L33930 | 934 | CD24 | CD24A |
| 203961_at | 0.142 | AL157398 | 10529 | NEBL | LNEBL; bA56H7.1; MGC119746; MGC119747 |
| 210413_x_at | 0.143 | U19557 | 6318 | SERPINB4 | PI11; SCCA1; SCCA2; LEUPIN; SCCA-2 |
| 232541_at | 0.143 | AK000106 | 1956 | EGFR | ERBB; mENA; ERBB1; EGFR |
| 211734_s_at | 0.143 | BC005912 | 2205 | FCER1A | FCE1A; FcERI |
| 226907_at | 0.144 | N32557 | 81706 | PPP1R14C | KEPI; NY-BR-81; CPI17-like |
| 214549_x_at | 0.144 | NM_005987 | 6698 | SPRR1A | SPRK |
| 205127_at | 0.145 | NM_000962 | 5742 | PTGS1 | COX1; COX3; PHS1; PCOX1; PGHS1; PTGHS; PGG/HS; PGHS-1; PTGS1 |
| 203780_at | 0.145 | AF275945 | 10205 | EVA1 | EVA; MPZL2; EVA1 |
| 217744_s_at | 0.145 | NM_022121 | 64065 | PERP | THW; KCP1; PIGPC1; KRTCAP1; dJ496H19.1; RP3-496H19.1 |
| 219532_at | 0.145 | NM_022726 | 6785 | ELOVL4 | ADMD; STGD2; STGD3 |
| 237839_at | 0.145 | BF433975 | 288 | ANK3 | FLJ45464; ANKYRIN-G; ANK3 |
| 212236_x_at | 0.145 | Z19574 | 3872 | KRT17 | PC; K17; PC2; PCHC1 |
| 209301_at | 0.146 | M36532 | 760 | CA2 | CAII; Car2; CA II; CA-II |
| 209771_x_at | 0.146 | AA761181 | 934 | CD24 | CD24A |
| 206295_at | 0.146 | NM_001562 | 3606 | IL18 | IGIF; IL-18; IL-1g; IL1F4; MGC12320 |
| 219529_at | 0.147 | NM_004669 | 9022 | CLIC3 | CLIC3 |
| 205765_at | 0.147 | NM_000777 | 1577 | CYP3A5 | CP35; PCN3; P450PCN3 |
| 222892_s_at | 0.149 | AI087937 | 55287 | TMEM40 | FLJ11036 |
| 220322_at | 0.149 | NM_019618 | 56300 | IL1F9 | IL1E; IL1H1; IL-1F9; IL-1H1; IL1RP2; IL-1RP2 |
| 1552532_a_at | 0.149 | NM_144583 | 245973 | ATP6V1C2 | VMA5; ATP6C2 |
| 222891_s_at | 0.149 | AI912275 | 53335 | BCL11A | EVI9; CTIP1; BCL11A-L; BCL11A-S; FLJ10173; FLJ34997; KIAA1809; BCL11A-XL |
| 221841_s_at | 0.149 | BF514079 | 9314 | KLF4 | EZF; GKLF |
| 1564307_a_at | 0.149 | AL832750 | 144568 | A2ML1 | CPAMD9; FLJ16045; FLJ25179; FLJ39129; FLJ41597; FLJ41598; FLJ41607; DKFZp686C1729; DKFZp686D2011; DKFZp686G1812; DKFZp686L1821; DKFZp686O1010 |
| 223631_s_at | 0.15 | AF213678 | 64073 | C19orf33 | IMUP; H2RSP; IMUP-1; IMUP-2; MGC39135; MGC75180 |
| 210426_x_at | 0.15 | U04897 | 6095 | RORA | ROR1; ROR2; ROR3; RZRA; NR1F1; MGC119326; MGC119329; RORA |
| 218180_s_at | 0.15 | NM_022772 | 64787 | EPS8L2 | EPS8R2; MGC3088; FLJ16738; FLJ21935; FLJ22171; MGC126530 |
| 216379_x_at | 0.151 | AK000168 | 934 | CD24 | CD24A |
| 208606_s_at | 0.151 | NM_030761 | 54361 | WNT4 | WNT-4 |
| 227752_at | 0.151 | AA005105 | 140911 | SPTLC2L |  |
| 206385_s_at | 0.152 | NM_020987 | 288 | ANK3 | FLJ45464; ANKYRIN-G; ANK3 |
| 235763_at | 0.152 | AA001450 | 204962 | SLC44A5 | CTL5; FLJ34081; MGC34032 |
| 226226_at | 0.152 | AI282982 | 120224 | TMEM45B | FLJ40787 |
| 218182_s_at | 0.152 | NM_021101 | 9076 | CLDN1 | CLD1; SEMP1; ILVASC |
| 205724_at | 0.152 | NM_000299 | 5317 | PKP1 | B6P; MGC138829; PKP1 |
| 237690_at | 0.154 | W67511 | 221393 | GPR115 | PGR18; FLJ38076 |
| 210715_s_at | 0.154 | AF027205 | 10653 | SPINT2 | PB; Kop; HAI2; HAI-2 |
| 242998_at | 0.154 | AI796235 | 145226 | RDH12 | LCA3; FLJ30273 |
| 224221_s_at | 0.154 | AF118886 | 10451 | VAV3 | FLJ40431 |
| 210085_s_at | 0.155 | AF230929 | 8416 | ANXA9 | ANX31 |
| 203126_at | 0.156 | NM_014214 | 3613 | IMPA2 | IMPA2 |
| 218677_at | 0.157 | NM_020672 | 57402 | S100A14 | BCMP84; S100A15 |
| 223541_at | 0.157 | AF232772 | 3038 | HAS3 | HAS3 |
| 1552477_a_at | 0.157 | BC014852 | 3664 | IRF6 | LPS; PIT; PPS; VWS; OFC6 |
| 220428_at | 0.157 | NM_015717 | 50489 | CD207 | CLEC4K; LANGERIN |
| 220289_s_at | 0.158 | NM_017977 | 55057 | AIM1L | FLJ10040 |
| 1553057_at | 0.158 | NM_080474 | 89777 | SERPINB12 | YUKOPIN; MGC119247; MGC119248 |
| 209493_at | 0.158 | AF338650 | 23037 | PDZD2 | AIPC; PIN1; PAPIN; PDZK3; KIAA0300 |
| 213929_at | 0.16 | AL050204 |  | - |  |
| 1557094_at | 0.16 | BC029890 | 244 | ANXA8 | ANX8; ANXA8L2; FLJ32754; VAC beta |
| 204268_at | 0.161 | NM_005978 | 6273 | S100A2 | CAN19; S100L; MGC111539 |
| 201667_at | 0.161 | NM_000165 | 2697 | GJA1 | CX43; GJAL; ODDD; DFNB38 |
| 205490_x_at | 0.161 | BF060667 | 2707 | GJB3 | EKV; CX31; DFNA2; FLJ22486; MGC102938; GJB3 |
| 214734_at | 0.161 | AB014524 | 23086 | EXPH5 | SLAC2-B; KIAA0624; MGC133291; MGC134967; DKFZp781H0795 |
| 203963_at | 0.161 | NM_001218 | 771 | CA12 | CAXII; FLJ20151; HsT18816; CA12 |
| 1555551_at | 0.162 | BC020713 | 5268 | SERPINB5 | PI5; maspin |
| 213707_s_at | 0.162 | NM_005221 | 1749 | DLX5 | DLX5 |
| 209125_at | 0.162 | J00269 | 3853; 286887 | KRT6A; KRT6C | K6A; K6C; K6D; CK6A; CK6C; CK6D; KRT6C; KRT6D; KRT6A; KRT6E; MGC102925; MGC163455; MGC163457 |
| 205683_x_at | 0.163 | NM_003294 | 7177 | TPSAB1 | TPS1; TPS2; TPSB1; alpha II |
| 203240_at | 0.163 | NM_003890 | 8857 | FCGBP | FC(GAMMA)BP |
| 230188_at | 0.163 | AW138350 | 348938 | NIPAL4 | ICHTHYIN; ICHYN |
| 201286_at | 0.164 | Z48199 | 6382 | SDC1 | SDC; CD138; SYND1; syndecan; SDC1 |
| 227735_s_at | 0.164 | AA553959 | 387695 | C10orf99 | UNQ1833; FLJ21763 |
| 219410_at | 0.165 | NM_018004 | 55076 | TMEM45A | DERP7; FLJ10134 |
| 234699_at | 0.165 | AJ131212 | 84659 | RNASE7 | MGC133220 |
| 223278_at | 0.165 | M86849 | 2706 | GJB2 | HID; KID; PPK; CX26; DFNA3; DFNB1; NSRD1 |
| 238451_at | 0.166 | BF693302 | 143098 | MPP7 | FLJ32798; RP11-218D6.5 |
| 33322_i_at | 0.166 | X57348 | 2810 | SFN | YWHAS |
| 208651_x_at | 0.166 | M58664 | 934 | CD24 | CD24A |
| 215784_at | 0.166 | AA309511 | 913 | CD1E | R2; CD1A |
| 227747_at | 0.167 | AA772172 |  | - |  |
| 1554897_s_at | 0.168 | BC013103 | 54933 | RHBDL2 | RRP2; MGC16997 |
| 220318_at | 0.168 | NM_017957 | 55040 | EPN3 | FLJ20778; MGC129899 |
| 219395_at | 0.168 | NM_024939 | 80004 | RBM35B | FLJ21918; FLJ22248 |
| 207114_at | 0.168 | NM_025261 | 80740 | LY6G6C | G6c; NG24; C6orf24 |
| 226560_at | 0.168 | AA576959 | 130367 | SGPP2 | SPP2; FLJ39004 |
| 206561_s_at | 0.168 | NM_020299 | 57016 | AKR1B10 | HIS; HSI; ARL1; ARL-1; ALDRLn; AKR1B11; AKR1B12; MGC14103 |
| 244692_at | 0.169 | AW025687 | 126410 | CYP4F22 | FLJ39501 |
| 210735_s_at | 0.169 | BC000278 | 771 | CA12 | CAXII; FLJ20151; HsT18816; CA12 |
| 223895_s_at | 0.169 | BC001038 | 55040 | EPN3 | FLJ20778; MGC129899 |
| 223544_at | 0.169 | BC005094 | 84283 | TMEM79 | FLJ16057; FLJ32254; MGC13102 |
| 227163_at | 0.169 | AL162742 | 119391 | GSTO2 | bA127L20.1 |
| 226755_at | 0.169 | AI375939 |  | - |  |
| 223739_at | 0.171 | AK026652 | 29943 | PADI1 | PDI; PAD1; PDI1; HPAD10 |
| 204393_s_at | 0.171 | NM_001099 | 55 | ACPP | PAP; ACP3; ACP-3 |
| 219497_s_at | 0.171 | NM_022893 | 53335 | BCL11A | EVI9; CTIP1; BCL11A-L; BCL11A-S; FLJ10173; FLJ34997; KIAA1809; BCL11A-XL |
| 222383_s_at | 0.171 | AW003512 | 59344 | ALOXE3 | eLOX3; MGC119694; MGC119695; MGC119696 |
| 216905_s_at | 0.172 | U20428 | 6768 | ST14 | HAI; MTSP1; SNC19; MT-SP1; MTSP-1; PRSS14; TADG-15 |
| 203021_at | 0.172 | NM_003064 | 6590 | SLPI | ALP; MPI; ALK1; BLPI; HUSI; WAP4; WFDC4; HUSI-I |
| 224022_x_at | 0.172 | AF169963 | 51384 | WNT16 | WNT16 |
| 214705_at | 0.173 | AJ001306 | 10207 | INADL | Cipp; PATJ; FLJ26982; INADL |
| 202597_at | 0.173 | AU144284 | 3664 | IRF6 | LPS; PIT; PPS; VWS; OFC6 |
| 228010_at | 0.175 | AI669212 | 5522 | PPP2R2C | PR52; PR55G; IMYPNO; IMYPNO1; MGC33570; PPP2R2C |
| 1554648_a_at | 0.176 | BC020841 | 90527 | DUOXA1 | NIP; mol; NUMBIP; FLJ32334 |
| 209159_s_at | 0.176 | AV724216 | 65009 | NDRG4 | SMAP-8; FLJ30586; FLJ42011; KIAA1180; MGC19632; DKFZp686I1615; NDRG4 |
| 222223_s_at | 0.177 | AF216693 | 26525 | IL1F5 | FIL1; FIL1D; IL1L1; IL1HY1; IL1RP3; MGC29840; FIL1(DELTA); IL1F5 |
| 220402_at | 0.177 | NM_022112 | 63970 | TP53AIP1 | P53AIP1 |
| 226817_at | 0.178 | AU154691 | 1824 | DSC2 | DG2; DSC3; CDHF2; ARVD11; DGII/III; DKFZp686I11137; DSC2 |
| 229377_at | 0.178 | BF001483 | 79774 | GRTP1 | TBC1D6; FLJ22474; MGC138328; MGC138330 |
| 202575_at | 0.178 | NM_001878 | 1382 | CRABP2 | RBP6; CRABP-II |
| 233488_at | 0.179 | AK023343 | 84659 | RNASE7 | MGC133220 |
| 203747_at | 0.18 | NM_004925 | 360 | AQP3 | GIL |
| 207023_x_at | 0.182 | NM_000421 | 3858 | KRT10 | K10; KPP; CK10 |
| 224999_at | 0.184 | BE878463 | 1956 | EGFR | ERBB; mENA; ERBB1; EGFR |
| 223316_at | 0.184 | AL136562 | 83643 | CCDC3 | FLJ20925; DKFZP761F241; RP11-347I22.1 |
| 226682_at | 0.184 | AW006185 | 283666 | - |  |
| 236225_at | 0.185 | AI697028 | 124975 | GGT6 | FLJ25990; FLJ90165 |
| 1552685_a_at | 0.186 | NM_014552 | 29841 | GRHL1 | MGR; LBP32; LBP-32; TFCP2L2; GRHL1 |
| 219795_at | 0.186 | NM_007231 | 11254 | SLC6A14 | ATB(0+) |
| 213506_at | 0.186 | BE965369 | 2150 | F2RL1 | PAR2; GPR11 |
| 238063_at | 0.186 | AA806283 | 201799 | TMEM154 | FLJ32028 |
| 203917_at | 0.187 | NM_001338 | 1525 | CXADR | CAR; HCAR |
| 231118_at | 0.187 | AW511401 | 148741 | ANKRD35 | FLJ25124; MGC126667; MGC126669 |
| 203962_s_at | 0.187 | NM_006393 | 10529 | NEBL | LNEBL; bA56H7.1; MGC119746; MGC119747 |
| 223681_s_at | 0.187 | AB044807 | 10207 | INADL | Cipp; PATJ; FLJ26982; INADL |
| 1562528_at | 0.188 | BC040965 | 6095 | RORA | ROR1; ROR2; ROR3; RZRA; NR1F1; MGC119326; MGC119329; RORA |
| 213110_s_at | 0.188 | AW052179 | 1287 | COL4A5 | ATS; ASLN; CA54; MGC42377; COL4A5 |
| 231867_at | 0.189 | AB032953 | 57451 | ODZ2 | TEN-M2; DKFZp686A1568 |
| 216641_s_at | 0.19 | U58994 | 3898 | LAD1 | LadA; MGC10355 |
| 226535_at | 0.191 | AK026736 | 3694 | ITGB6 | ITGB6 |
| 227180_at | 0.192 | AW138767 | 79993 | ELOVL7 | FLJ23563 |
| 204400_at | 0.192 | NM_005864 | 10278 | EFS | SIN; EFS1; EFS2; HEFS |
| 234331_s_at | 0.193 | AK025063 | 151354 | FAM84A | NSE1; PP11517; FLJ35392 |
| 209873_s_at | 0.193 | AF053719 | 11187 | PKP3 | PKP3 |
| 226374_at | 0.193 | BG260087 |  | - |  |
| 205014_at | 0.194 | NM_005130 | 9982 | FGFBP1 | FGFBP; HBP17 |
| 210479_s_at | 0.195 | L14611 | 6095 | RORA | ROR1; ROR2; ROR3; RZRA; NR1F1; MGC119326; MGC119329; RORA |
| 215243_s_at | 0.195 | AF099730 | 2707 | GJB3 | EKV; CX31; DFNA2; FLJ22486; MGC102938; GJB3 |
| 217014_s_at | 0.195 | AC004522 | 563 | AZGP1 | ZAG; ZA2G |
| 235247_at | 0.198 | AI224578 | 79977 | GRHL2 | BOM; DFNA28; TFCP2L3; FLJ11172; FLJ13782; MGC149294; MGC149295 |
| 207134_x_at | 0.198 | NM_024164 | 64499 | TPSB2 | TPS2; TPSB1; tryptaseC |
| 202712_s_at | 0.198 | NM_020990 | 548596; 1159 | CKMT1A; CKMT1B | CKMT1; UMTCK |
| 240382_at | 0.198 | AW444944 | 1832 | DSP | DPI; DPII; DSP |
| 228948_at | 0.199 | T15545 | 2043 | EPHA4 | SEK; HEK8; TYRO1 |
| 1555173_at | 0.2 | BC034696 | 415117 | STX19 | MGC21382 |
| 227475_at | 0.201 | AI676059 | 94234 | FOXQ1 | HFH1 |
| 215314_at | 0.201 | AU146646 | 288 | ANK3 | FLJ45464; ANKYRIN-G; ANK3 |
| 204718_at | 0.202 | NM_004445 | 2051 | EPHB6 | HEP; MGC129910; MGC129911 |
| 205109_s_at | 0.202 | NM_015320 | 50649 | ARHGEF4 | ASEF; GEF4; STM6; ARHGEF4 |
| 214164_x_at | 0.202 | BF752277 | 771 | CA12 | CAXII; FLJ20151; HsT18816; CA12 |
| 201287_s_at | 0.204 | NM_002997 | 6382 | SDC1 | SDC; CD138; SYND1; syndecan; SDC1 |
| 203287_at | 0.205 | NM_005558 | 3898 | LAD1 | LadA; MGC10355 |
| 210084_x_at | 0.205 | AF206665 | 7177 | TPSAB1 | TPS1; TPS2; TPSB1; alpha II |
| 219010_at | 0.206 | NM_018265 | 55765 | C1orf106 | FLJ10901; MGC125608 |
| 213421_x_at | 0.206 | AW007273 | 5646 | PRSS3 | MTG; TRY3; TRY4; PRSS4 |
| 204755_x_at | 0.207 | M95585 | 3131 | HLF | MGC33822 |
| 227134_at | 0.208 | AI341537 | 84958 | SYTL1 | JFC1; SLP1; FLJ14996 |
| 219764_at | 0.209 | NM_007197 | 11211 | FZD10 | FzE7; FZ-10; hFz10 |
| 223821_s_at | 0.211 | BC004888 | 55061 | SUSD4 | PRO222; FLJ10052; RP11-239E10.4; SUSD4 |
| 212242_at | 0.213 | AL565074 | 7277 | TUBA4A | TUBA1; FLJ30169; H2-ALPHA |
| 210074_at | 0.214 | AF070448 | 1515 | CTSL2 | CTSU; CTSV; CATL2; MGC125957 |
| 230769_at | 0.214 | AI916261 | 163259 | DENND2C | FLJ37099; dJ1156J9.1; RP5-1156J9.1; DKFZp686G0351; DKFZp686N1631; DKFZp779P1149 |
| 219461_at | 0.214 | AJ236915 | 56924 | PAK6 | PAK5 |
| 202289_s_at | 0.215 | NM_006997 | 10579 | TACC2 | AZU-1; ECTACC; TACC2 |
| 220161_s_at | 0.215 | NM_019114 | 54566 | EPB41L4B | CG1; EHM2; FLJ21596; DKFZp761N1814; EPB41L4B |
| 229546_at | 0.216 | AI378035 | 151354 | FAM84A | NSE1; PP11517; FLJ35392 |
| 204753_s_at | 0.217 | AI810712 | 3131 | HLF | MGC33822 |
| 217496_s_at | 0.218 | AA918442 | 3416 | IDE | FLJ35968; INSULYSIN |
| 226064_s_at | 0.218 | AW469523 | 84649 | DGAT2 | HMFN1045; DKFZp686A15125 |
| 1554253_a_at | 0.219 | BC028703 | 204219 | LASS3 | MGC27091 |
| 228698_at | 0.219 | AI808807 | 83595 | SOX7 | MGC10895 |
| 208153_s_at | 0.219 | NM_001447 | 2196 | FAT2 | CDHF8; HFAT2; MEGF1 |
| 203726_s_at | 0.22 | NM_000227 | 3909 | LAMA3 | E170; LOCS; LAMNA; lama3a; LAMA3 |
| 210130_s_at | 0.22 | AF096304 | 7108 | TM7SF2 | ANG1; DHCR14A |
| 213287_s_at | 0.22 | X14487 | 3858 | KRT10 | K10; KPP; CK10 |
| 218792_s_at | 0.221 | NM_017688 | 54836 | BSPRY | FLJ20150 |
| 211272_s_at | 0.221 | AF064771 | 1606 | DGKA | DAGK; DAGK1; MGC12821; MGC42356; DGK-alpha; DGKA |
| 210325_at | 0.221 | M28825 | 909 | CD1A | R4; T6; CD1; FCB6; HTA1 |
| 208937_s_at | 0.222 | D13889 | 3397 | ID1 | ID; ID1 |
| 226185_at | 0.222 | AK026697 | 1040 | CDS1 | CDS |
| 204039_at | 0.224 | NM_004364 | 1050 | CEBPA | CEBP; C/EBP-alpha |
| 207018_s_at | 0.224 | NM_004163 | 5874 | RAB27B | RAB27B |
| 219908_at | 0.225 | NM_014421 | 27123 | DKK2 | DKK-2 |
| 204990_s_at | 0.225 | NM_000213 | 3691 | ITGB4 | CD104; ITGB4 |
| 204537_s_at | 0.225 | NM_004961 | 2564 | GABRE | GABRE |
| 206581_at | 0.226 | NM_001717 | 646 | BNC1 | BNC; BSN1; HsT19447 |
| 1558846_at | 0.226 | AL833418 | 119548 | PNLIPRP3 | PNLIPRP3 |
| 230867_at | 0.226 | AI742521 | 131873 | COL6A6 | LOC131873 |
| 218736_s_at | 0.227 | NM_017734 | 54873 | PALMD | PALML; C1orf11; FLJ20271 |
| 241356_at | 0.227 | BG290650 |  | - |  |
| 230233_at | 0.23 | BF110534 | 153020 | RASGEF1B | GPIG4; FLJ31695; MGC46251 |
| 231311_at | 0.23 | AA725246 | 6712 | SPTBN2 | SCA5 |
| 1552502_s_at | 0.23 | NM_017821 | 54933 | RHBDL2 | RRP2; MGC16997 |
| 214235_at | 0.23 | X90579 | 1577 | CYP3A5 | CP35; PCN3; P450PCN3 |
| 219916_s_at | 0.231 | NM_025236 | 80352 | RNF39 | HZF; HZFW; LIRF; RNF39 |
| 238669_at | 0.232 | BE613133 | 5742 | PTGS1 | COX1; COX3; PHS1; PCOX1; PGHS1; PTGHS; PGG/HS; PGHS-1; PTGS1 |
| 236201_at | 0.232 | N30188 |  | - |  |
| 202687_s_at | 0.233 | U57059 | 8743 | TNFSF10 | TL2; APO2L; CD253; TRAIL; Apo-2L |
| 222895_s_at | 0.233 | AA918317 | 64919 | BCL11B | RIT1; CTIP2; CTIP-2; hRIT1-alpha; BCL11B |
| 215813_s_at | 0.233 | S36219 | 5742 | PTGS1 | COX1; COX3; PHS1; PCOX1; PGHS1; PTGHS; PGG/HS; PGHS-1; PTGS1 |
| 238439_at | 0.233 | AI925518 | 118932 | ANKRD22 | MGC22805 |
| 204058_at | 0.234 | AL049699 | 4199 | ME1 | MES; HUMNDME |
| 210480_s_at | 0.235 | U90236 | 4646 | MYO6 | DFNA22; DFNB37; KIAA0389 |
| 215536_at | 0.235 | X87344 | 3120 | HLA-DQB2 | HLA-DXB |
| 214945_at | 0.235 | AW514267 | 285596 | FAM153A | NY-REN-7; KIAA0752 |
| 219998_at | 0.236 | NM_014181 | 29094 | - | HSPC159; GRP; MGC33751; MGC71953 |
| 215867_x_at | 0.237 | AL050025 | 771 | CA12 | CAXII; FLJ20151; HsT18816; CA12 |
| 238692_at | 0.238 | AL040935 | 121551 | BTBD11 | FLJ33957; FLJ42845; BTBD11 |
| 215395_x_at | 0.238 | U66061 | 154754 | PRSS3P2 | TRY6; T6 |
| 1570585_at | 0.239 | BC031223 | 196264 | MPZL3 | LOC196264 |
| 243278_at | 0.239 | AW291402 |  | - |  |
| 91826_at | 0.239 | AI219073 | 54869 | EPS8L1 | DRC3; EPS8R1; MGC4642; PP10566; FLJ20258; MGC23164; EPS8L1 |
| 1555716_a_at | 0.24 | AY072911 | 1525 | CXADR | CAR; HCAR |
| 205128_x_at | 0.24 | NM_000962 | 5742 | PTGS1 | COX1; COX3; PHS1; PCOX1; PGHS1; PTGHS; PGG/HS; PGHS-1; PTGS1 |
| 1569144_a_at | 0.24 | BC018787 | 375791 | C9orf169 | MGC59937 |
| 1553835_a_at | 0.241 | NM_153264 | 256076 | COL6A5 | VWA4; COL29A1; FLJ35880 |
| 222392_x_at | 0.241 | AJ251830 | 64065 | PERP | THW; KCP1; PIGPC1; KRTCAP1; dJ496H19.1; RP3-496H19.1 |
| 226382_at | 0.242 | AA528080 | 283070 | LOC283070 | LOC283070 |
| 219528_s_at | 0.243 | NM_022898 | 64919 | BCL11B | RIT1; CTIP2; CTIP-2; hRIT1-alpha; BCL11B |
| 201984_s_at | 0.243 | NM_005228 | 1956 | EGFR | ERBB; mENA; ERBB1; EGFR |
| 224555_x_at | 0.245 | AF251120 | 27178 | IL1F7 | FIL1; FIL1Z; IL1H4; IL-1F7; IL-1H4; IL1RP1; IL-1RP1; FIL1(ZETA); IL1F7 |
| 218849_s_at | 0.246 | NM_006663 | 10848 | PPP1R13L | RAI; IASPP; NKIP1; iASPP gene |
| 225792_at | 0.247 | AA618420 | 51361 | HOOK1 | HK1; MGC10642 |
| 208078_s_at | 0.248 | NM_030751 | 150094 | SNF1LK | MSK; SIK |
| 230986_at | 0.248 | AI821447 | 11279 | KLF8 | BKLF3; DXS741; ZNF741; MGC138314; DKFZp686O08126 |
| 200635_s_at | 0.248 | AU145351 | 5792 | PTPRF | LAR; FLJ43335; FLJ45062; FLJ45567; PTPRF |
| 1553212_at | 0.249 | NM_173352 | 196374 | KRT78 | K5B; Kb40 |
| 233025_at | 0.249 | AU146117 | 23037 | PDZD2 | AIPC; PIN1; PAPIN; PDZK3; KIAA0300 |
| 219121_s_at | 0.249 | NM_017697 | 54845 | RBM35A | FLJ20171; RBM35A |
| 239370_at | 0.249 | AW081982 |  | - |  |
| 219695_at | 0.25 | NM_024703 | 55512 | SMPD3 | NSMASE2; FLJ22593; MGC138443 |
| 238567_at | 0.25 | AW779536 | 130367 | SGPP2 | SPP2; FLJ39004 |
| 205623_at | 0.25 | NM_000691 | 218 | ALDH3A1 | ALDH3; ALDHIII; MGC10406 |
| 210633_x_at | 0.25 | M19156 | 3858 | KRT10 | K10; KPP; CK10 |
| 203215_s_at | 0.251 | AA877789 | 4646 | MYO6 | DFNA22; DFNB37; KIAA0389 |
| 232360_at | 0.252 | AA565141 | 26298 | EHF | ESE3; ESEJ |
| 233280_at | 0.252 | AU159446 | 10529 | NEBL | LNEBL; bA56H7.1; MGC119746; MGC119747 |
| 233565_s_at | 0.252 | AL136531 | 27111 | SDCBP2 | ST-2; SITAC18; FLJ12256; SDCBP2 |
| 211362_s_at | 0.252 | AF169949 | 5275 | SERPINB13 | HUR7; PI13; headpin; MGC126870 |
| 238512_at | 0.254 | BF961733 | 829 | CAPZA1 | CAPZ; CAZ1; CAPPA1 |
| 229396_at | 0.254 | AA588400 | 5017 | OVOL1 | HOVO1 |
| 1569886_a_at | 0.254 | BC040605 | 283176 | FLJ90231 | FLJ90231 |
| 216474_x_at | 0.255 | AF206667 | 7177; 64499 | TPSAB1 | TPS1; TPS2; TPSB1; alpha II; tryptaseC |
| 1559606_at | 0.255 | AL703282 | 163351 | GBP6 | DKFZp686G0786 |
| 238967_at | 0.255 | AI924046 | 9076 | CLDN1 | CLD1; SEMP1; ILVASC |
| 219388_at | 0.256 | NM_024915 | 79977 | GRHL2 | BOM; DFNA28; TFCP2L3; FLJ11172; FLJ13782; MGC149294; MGC149295 |
| 207463_x_at | 0.257 | NM_002771 | 5646 | PRSS3 | MTG; TRY3; TRY4; PRSS4 |
| 238513_at | 0.257 | BF905445 | 79056 | PRRG4 | TMG4 |
| 223822_at | 0.257 | BC004888 | 55061 | SUSD4 | PRO222; FLJ10052; RP11-239E10.4 |
| 238028_at | 0.258 | BE379393 | 389389 | - | FLJ90086 |
| 202525_at | 0.258 | NM_002773 | 5652 | PRSS8 | CAP1; PROSTASIN |
| 229385_s_at | 0.258 | AI743780 |  | - |  |
| 219825_at | 0.259 | NM_019885 | 56603 | CYP26B1 | CYP26A2; MGC129613; P450RAI-2; DKFZp686G0638 |
| 1563217_at | 0.259 | BI598831 | 5569 | PKIA | PRKACN1; PKIA |
| 243722_at | 0.259 | W73523 | 260434 | PYDC1 | ASC2; POP1; PYC1 |
| 231928_at | 0.26 | AK023754 | 54626 | HES2 | HES2 |
| 226612_at | 0.26 | H17038 | 134111 | UBE2QL1 | FLJ25076; FLJ39293 |
| 219498_s_at | 0.26 | NM_018014 | 53335 | BCL11A | EVI9; CTIP1; BCL11A-L; BCL11A-S; FLJ10173; FLJ34997; KIAA1809; BCL11A-XL |
| 203797_at | 0.261 | AF039555 | 7447 | VSNL1 | HLP3; VILIP; HPCAL3; HUVISL1; VILIP-1 |
| 209373_at | 0.261 | BC003179 | 7851 | MALL | BENE; MGC4419 |
| 236220_at | 0.261 | AI935541 |  | - |  |
| 221665_s_at | 0.261 | BC004907 | 54869 | EPS8L1 | DRC3; EPS8R1; MGC4642; PP10566; FLJ20258; MGC23164; EPS8L1 |
| 201425_at | 0.262 | NM_000690 | 217 | ALDH2 | ALDM; ALDHI; ALDH-E2; MGC1806 |
| 225301_s_at | 0.262 | AI991160 | 4645 | MYO5B | KIAA1119 |
| 221795_at | 0.262 | AI346341 | 4915 | NTRK2 | TRKB; GP145-TrkB; NTRK2 |
| 226188_at | 0.262 | AK025603 | 29094 | LGALSL | HSPC159; GRP; MGC33751; MGC71953 |
| 203997_at | 0.264 | NM_002829 | 5774 | PTPN3 | PTPH1; DKFZp686N0569 |
| 209699_x_at | 0.265 | U05598 | 1646 | AKR1C2 | DD; DD2; BABP; DDH2; HBAB; HAKRD; MCDR2; AKR1C-pseudo; AKR1C2 |
| 225078_at | 0.265 | AV686514 | 2013 | EMP2 | XMP; MGC9056 |
| 218806_s_at | 0.265 | AF118887 | 10451 | VAV3 | FLJ40431 |
| 242064_at | 0.265 | N23651 | 54549 | SDK2 | FLJ10832; KIAA1514 |
| 203328_x_at | 0.266 | NM_004969 | 3416 | IDE | FLJ35968; INSULYSIN |
| 1554062_at | 0.269 | AF380356 | 7499 | XG | PBDX; MGC118758; MGC118759; MGC118760; MGC118761 |
| 211382_s_at | 0.269 | AF220152 | 10579 | TACC2 | AZU-1; ECTACC; TACC2 |
| 220056_at | 0.27 | NM_021258 | 58985 | IL22RA1 | IL22R; CRF2-9 |
| 204765_at | 0.271 | NM_005435 | 7984 | ARHGEF5 | P60; TIM; GEF5; TIM1; DKFZp686N1969 |
| 238752_at | 0.271 | AA780295 | 57380 | MRS2L | HPT; MRS2; MGC78523 |
| 215800_at | 0.272 | AL137592 | 53905 | DUOX1 | LNOX1; THOX1; NOXEF1; MGC138840; MGC138841; DUOX1 |
| 222847_s_at | 0.272 | AI378406 | 112399 | EGLN3 | PHD3; HIFPH3; FLJ21620; MGC125998; MGC125999 |
| 223694_at | 0.272 | AF220032 | 81786 | TRIM7 | GNIP; RNF90; TRIM7 |
| 201328_at | 0.273 | AL575509 | 2114 | ETS2 | ETS2 |
| 40016_g_at | 0.273 | AB002301 | 23227 | MAST4 |  |
| 226865_at | 0.274 | AW130600 |  | - |  |
| 210239_at | 0.274 | U90304 | 10265 | IRX5 | IRX-2a |
| 225615_at | 0.274 | AK024480 | 126917 | IFFO2 |  |
| 221295_at | 0.275 | NM_001279 | 1149 | CIDEA | CIDE-A; CIDEA |
| 212543_at | 0.276 | U83115 | 202 | AIM1 | ST4 |
| 232277_at | 0.276 | AA643687 | 64078 | SLC28A3 | CNT3 |
| 227642_at | 0.277 | AI928242 | 29842 | TFCP2L1 | LBP9; CRTR1; LBP-9 |
| 1556194_a_at | 0.278 | BC042959 |  | - |  |
| 216470_x_at | 0.278 | AF009664 | 5644; 5645; 5646; 154754 | TRP1; TRY1; TRY4; TRYP1 | TRP1; TRY1; TRY4; TRYP1; MGC120175; MGC149362; TRY2; TRY8; TRYP2; MGC111183; MGC120174; MTG; TRY3; PRSS4; TRY6; T6 |
| 218484_at | 0.278 | NM_020142 | 56901 | NDUFA4L2 | NUOMS; FLJ26118 |
| 219423_x_at | 0.278 | NM_003790 | 8718 | TNFRSF25 | DR3; TR3; DDR3; LARD; APO-3; TRAMP; WSL-1; WSL-LR; TNFRSF12; TNFRSF25 |
| 200907_s_at | 0.28 | AU157932 | 23022 | PALLD | PNCA1; SIH002; CGI-151; FLJ22190; FLJ38193; FLJ39139; KIAA0992 |
| 226187_at | 0.281 | AW304313 | 1040 | CDS1 | CDS |
| 1553695_a_at | 0.281 | NM_170722 | 79671 | NLRX1 | NOD5; NOD9; NOD26; DLNB26; CLR11.3; FLJ21478; MGC21025; MGC131937; NLRX1 |
| 210058_at | 0.282 | BC000433 | 5603 | MAPK13 | SAPK4; PRKM13; MGC99536; p38delta |
| 238702_at | 0.282 | BG287503 | 165679 | C3orf57 |  |
| 210347_s_at | 0.282 | AF080216 | 53335 | BCL11A | EVI9; CTIP1; BCL11A-L; BCL11A-S; FLJ10173; FLJ34997; KIAA1809; BCL11A-XL |
| 202085_at | 0.282 | NM_004817 | 9414 | TJP2 | ZO2; X104; ZO-2; MGC26306; TJP2 |
| 224327_s_at | 0.283 | AB048286 | 84649 | DGAT2 | HMFN1045; DKFZp686A15125 |
| 204726_at | 0.283 | NM_001257 | 1012 | CDH13 | CDHH |
| 209387_s_at | 0.283 | M90657 | 4071 | TM4SF1 | L6; H-L6; M3S1; TAAL6 |
| 227314_at | 0.284 | N95414 | 3673 | ITGA2 | BR; GPIa; CD49B; VLA-2; VLAA2 |
| 231439_at | 0.284 | AA922936 | 151354 | FAM84A | NSE1; PP11517; FLJ35392 |
| 205987_at | 0.285 | NM_001765 | 911 | CD1C | R7; CD1; CD1A |
| 204388_s_at | 0.285 | NM_000240 | 4128 | MAOA | MAOA |
| 242784_at | 0.286 | AV646177 | 2114 | ETS2 | ETS2 |
| 212992_at | 0.286 | AI935123 | 113146 | AHNAK2 | C14orf78; KIAA2019 |
| 206115_at | 0.286 | NM_004430 | 1960 | EGR3 | PILOT; MGC138484 |
| 209203_s_at | 0.287 | BC002327 | 23299 | BICD2 | KIAA0699; bA526D8.1; BICD2 |
| 207911_s_at | 0.288 | NM_004245 | 9333 | TGM5 | TGX; TGM6; TGMX; MGC141907; TGM5 |
| 232017_at | 0.289 | AK025185 | 9414 | TJP2 | ZO2; X104; ZO-2; MGC26306; TJP2 |
| 209679_s_at | 0.289 | BC003379 | 57228 | SMAGP | LOC57228; MGC149453; MGC149454 |
| 238654_at | 0.289 | W79425 | 147645 | VSIG10L | LOC147645 |
| 242662_at | 0.29 | AI056815 | 5046 | PCSK6 | SPC4; PACE4; PCSK6 |
| 226789_at | 0.291 | W84421 | 440609 | - |  |
| 202005_at | 0.291 | NM_021978 | 6768 | ST14 | HAI; MTSP1; SNC19; MT-SP1; MTSP-1; PRSS14; TADG-15 |
| 228570_at | 0.291 | BF510581 | 121551 | BTBD11 | FLJ33957; FLJ42845; BTBD11 |
| 220413_at | 0.291 | NM_014579 | 29986 | SLC39A2 | ZIP2; MGC119190 |
| 221107_at | 0.291 | NM_017581 | 55584 | CHRNA9 | NACHRA9; HSA243342; MGC142109; MGC142135 |
| 239726_at | 0.291 | AI743588 |  | - |  |
| 220658_s_at | 0.292 | NM_020183 | 56938 | ARNTL2 | CLIF; MOP9; BMAL2; PASD9; MGC149671; MGC149672 |
| 241397_at | 0.292 | AW276866 | 26298 | EHF | ESE3; ESEJ |
| 218804_at | 0.292 | NM_018043 | 55107 | TMEM16A | TAOS2; ORAOV2; FLJ10261 |
| 227393_at | 0.292 | AW084755 | 338440 | TMEM16J | PIG5; TP53I5 |
| 209270_at | 0.293 | L25541 | 3914 | LAMB3 | LAMNB1; LAMB3 |
| 204005_s_at | 0.293 | NM_002583 | 5074 | PAWR | PAR4; Par-4 |
| 204975_at | 0.293 | NM_001424 | 2013 | EMP2 | XMP; MGC9056 |
| 1555382_at | 0.293 | BC017500 | 79983 | POF1B | POF; FLJ22792 |
| 205807_s_at | 0.294 | NM_020127 | 7286 | TUFT1 | TUFT1 |
| 219368_at | 0.294 | NM_021963 | 4674 | NAP1L2 | BPX; MGC26243 |
| 218810_at | 0.294 | NM_025079 | 80149 | ZC3H12A | MCPIP; FLJ23231; dJ423B22.1; RP3-423B22.1 |
| 214329_x_at | 0.295 | AW474434 | 8743 | TNFSF10 | TL2; APO2L; CD253; TRAIL; Apo-2L |
| 1555310_a_at | 0.295 | BC035596 | 56924 | PAK6 | PAK5 |
| 1564333_a_at | 0.295 | AK097698 | 57537 | SORCS2 | SORCS2 |
| 227461_at | 0.296 | AA632295 | 85439 | STON2 | STN2; STNB; STNB2 |
| 207382_at | 0.296 | NM_003722 | 8626 | TP73L | KET; LMS; RHS; p51; p63; EEC3; OFC8; TP63; p73H; p73L; SHFM4; B(p51A); B(p51B) |
| 224013_s_at | 0.296 | BC004299 | 83595 | SOX7 | MGC10895 |
| 1552319_a_at | 0.296 | NM_144506 | 11202 | KLK8 | NP; HNP; NRPN; PRSS19; TADG14; KLK8 |
| 223484_at | 0.297 | AF228422 | 84419 | C15orf48 | NMES1; FOAP-11; FLJ22645; MGC32925; C15orf48 |
| 35666_at | 0.297 | U38276 | 6405 | SEMA3F | SEMA4; SEMAK; SEMA-IV; sema IV |
| 239155_at | 0.297 | N34895 | 1525 | CXADR | CAR; HCAR |
| 206453_s_at | 0.298 | NM_016250 | 57447 | NDRG2 | SYLD; FLJ25522; KIAA1248; DKFZp781G1938; NDRG2 |
| 222932_at | 0.299 | AF203977 | 26298 | EHF | ESE3; ESEJ |
| 213933_at | 0.299 | AW242315 | 5733 | PTGER3 | EP3; EP3e; EP3-I; EP3-II; EP3-IV; EP3-III; MGC27302; MGC141828; MGC141829; PTGER3 |
| 207980_s_at | 0.299 | NM_006079 | 10370 | CITED2 | MRG1; P35SRJ |
| 238755_at | 0.299 | AA876179 |  | - |  |
| 233388_at | 0.299 | AK022350 | 771 | CA12 | CAXII; FLJ20151; HsT18816; CA12 |
| 205783_at | 0.3 | NM_015596 | 26085 | KLK13 | KLKL4; KLK-L4; DKFZP586J1923 |
| 219850_s_at | 0.3 | NM_012153 | 26298 | EHF | ESE3; ESEJ |
| 219930_at | 0.3 | NM_007250 | 11279 | KLF8 | BKLF3; DXS741; ZNF741; MGC138314; DKFZp686O08126 |
| 224209_s_at | 0.301 | AF019638 | 9615 | GDA | CYPIN; GUANASE; MGC9982; NEDASIN; KIAA1258 |
| 244447_at | 0.301 | AW292830 | 7071 | KLF10 | EGRA; TIEG; TIEG1; KLF10 |
| 215239_x_at | 0.301 | AU132789 | 10793 | ZNF273 | HZF9; MGC12518 |
| 212989_at | 0.301 | AI377497 | 259230 | SGMS1 | MOB; MOB1; SMS1; TMEM23; MGC17342 |
| 230518_at | 0.301 | BF437750 | 10205 | EVA1 | EVA; MPZL2; EVA1 |
| 220066_at | 0.301 | NM_022162 | 64127 | NOD2 | CD; ACUG; BLAU; IBD1; NLRC2; NOD2B; CARD15; CLR16.3; PSORAS1 |
| 236172_at | 0.301 | AW206817 | 1241 | LTB4R | BLT1; BLTR; P2Y7; GPR16; LTBR1; P2RY7; CMKRL1; LTB4R1 |
| 200965_s_at | 0.301 | NM_006720 | 3983 | ABLIM1 | ABLIM; LIMAB1; LIMATIN; MGC1224; FLJ14564; KIAA0059; DKFZp781D0148; ABLIM1 |
| 205403_at | 0.302 | NM_004633 | 7850 | IL1R2 | IL1RB; CD121b; MGC47725; IL1R2 |
| 1562529_s_at | 0.302 | BC040965 | 6095 | RORA | ROR1; ROR2; ROR3; RZRA; NR1F1; MGC119326; MGC119329; RORA |
| 225611_at | 0.303 | AI922968 | 23227 | MAST4 |  |
| 207076_s_at | 0.303 | NM_000050 | 445 | ASS1 | ASS; CTLN1; ASS1 |
| 229674_at | 0.303 | AL035414 | 56256 | SERTAD4 | DJ667H12.2 |
| 203585_at | 0.304 | NM_007150 | 7739 | ZNF185 | ZNF185 |
| 227717_at | 0.304 | BF939317 | 389337 | ARHGEF37 | FLJ41603 |
| 207655_s_at | 0.304 | NM_013314 | 29760 | BLNK | BASH; Ly57; SLP65; BLNK-s; SLP-65; MGC111051 |
| 230076_at | 0.305 | BF063164 | 54478 | FAM64A | FLJ10156; FLJ10491 |
| 225846_at | 0.305 | BF001941 | 54845 | RBM35A | FLJ20171; RBM35A |
| 217234_s_at | 0.306 | AF199015 | 7430 | VIL2 | CVL; CVIL; MGC1584; FLJ26216; DKFZp762H157 |
| 202688_at | 0.306 | NM_003810 | 8743 | TNFSF10 | TL2; APO2L; CD253; TRAIL; Apo-2L |
| 1565863_at | 0.306 | AF085948 | 9414 | TJP2 | ZO2; X104; ZO-2; MGC26306; TJP2 |
| 225079_at | 0.308 | AI089325 | 2013 | EMP2 | XMP; MGC9056 |
| 200636_s_at | 0.308 | NM_002840 | 5792 | PTPRF | LAR; FLJ43335; FLJ45062; FLJ45567; PTPRF |
| 227944_at | 0.309 | BF437260 | 5774 | PTPN3 | PTPH1; DKFZp686N0569 |
| 213285_at | 0.31 | AV691491 | 161291 | TMEM30B | CDC50B; MGC126775 |
| 226177_at | 0.31 | AI052020 | 51228 | GLTP | GLTP |
| 205569_at | 0.311 | NM_014398 | 27074 | LAMP3 | LAMP; CD208; DCLAMP; TSC403; DC-LAMP |
| 213618_at | 0.311 | AB011152 | 116984 | CENTD1 | PARX; ARAP2; FLJ13675; FLJ44916; KIAA0580; CENTD1 |
| 1557136_at | 0.311 | BG059633 | 84239 | ATP13A4 | MGC126545; DKFZp761I1011 |
| 218454_at | 0.312 | NM_024829 | 79887 | PLBD1 | FLJ22662 |
| 223611_s_at | 0.313 | AI738919 | 84708 | LNX1 | LNX; MPDZ; PDZRN2 |
| 225536_at | 0.313 | AL545105 | 113452 | TMEM54 | BCLP; CAC1; CAC-1; MGC10137 |
| 226908_at | 0.313 | AI627704 | 121227 | LRIG3 | FLJ26573; FLJ90440; KIAA3016 |
| 1553132_a_at | 0.314 | NM_152332 | 123036 | MTAC2D1 | Tac2-N; C14orf47; FLJ36557; c14_5695 |
| 219517_at | 0.314 | NM_025165 | 80237 | ELL3 | FLJ22637 |
| 227034_at | 0.315 | BE669553 | 65124 | ANKRD57 | C2orf26 |
| 228567_at | 0.315 | BG109230 |  | - |  |
| 222303_at | 0.316 | AV700891 |  | - |  |
| 201041_s_at | 0.318 | NM_004417 | 1843 | DUSP1 | HVH1; MKP1; CL100; MKP-1; PTPN10 |
| 207206_s_at | 0.318 | NM_000697 | 239 | ALOX12 | LOG12; 12-LOX |
| 209555_s_at | 0.319 | M98399 | 948 | CD36 | FAT; GP4; GP3B; GPIV; CHDS7; PASIV; SCARB3; CD36 |
| 200897_s_at | 0.319 | NM_016081 | 23022 | PALLD | PNCA1; SIH002; CGI-151; FLJ22190; FLJ38193; FLJ39139; KIAA0992 |
| 228067_at | 0.319 | AW249666 | 343990 | C2orf55 | MGC42367 |
| 233026_s_at | 0.32 | AU146117 | 23037 | PDZD2 | AIPC; PIN1; PAPIN; PDZK3; KIAA0300 |
| 218451_at | 0.32 | NM_022842 | 64866 | CDCP1 | CD318; TRASK; SIMA135; CDCP1 |
| 1438_at | 0.32 | X75208 | 2049 | EPHB3 | ETK2; HEK2; TYRO6 |
| 229720_at | 0.32 | AA394039 | 573 | BAG1 | BAG1 |
| 203413_at | 0.321 | NM_006159 | 4753 | NELL2 | NRP2 |
| 202345_s_at | 0.322 | NM_001444 | 2171 | FABP5 | EFABP; E-FABP; PAFABP; PA-FABP |
| 229070_at | 0.322 | AA470369 | 84830 | C6orf105 | MGC12335; dJ413H6.1; RP3-413H6.1 |
| 226302_at | 0.322 | BG290908 |  | - |  |
| 222859_s_at | 0.322 | AA150186 | 27071 | DAPP1 | BAM32; DKFZp667E0716 |
| 232158_x_at | 0.323 | AU158253 | 152519 | NPAL1 | DKFZp686A06115 |
| 228964_at | 0.323 | AI692659 | 639 | PRDM1 | BLIMP1; PRDI-BF1; MGC118922; MGC118923; MGC118924; MGC118925; PRDM1 |
| 239153_at | 0.323 | BF109906 | 400041 | FLJ41747 | FLJ41747 |
| 210059_s_at | 0.323 | BC000433 | 5603 | MAPK13 | SAPK4; PRKM13; MGC99536; p38delta |
| 243636_s_at | 0.324 | AI042373 | 152503 | SH3D19 | SH3D19; EBP; EVE1; MGC105136; MGC118910; MGC118911; MGC118912; MGC118913 |
| 230252_at | 0.325 | AW183080 | 57121 | GPR92 | LPA5; GPR93; LPAR5; KPG_010 |
| 216594_x_at | 0.325 | S68290 | 1645 | AKR1C1 | C9; DD1; DDH; DDH1; H-37; MBAB; HAKRC; MGC8954; 2-ALPHA-HSD; 20-ALPHA-HSD |
| 227995_at | 0.325 | AI051950 |  | - |  |
| 202267_at | 0.326 | NM_005562 | 3918 | LAMC2 | B2T; EBR2; BM600; EBR2A; LAMB2T; LAMNB2; MGC138491; MGC141938; LAMC2 |
| 201329_s_at | 0.326 | NM_005239 | 2114 | ETS2 | ETS2 |
| 1559190_s_at | 0.326 | AL833150 |  | - |  |
| 208622_s_at | 0.327 | AA670344 | 7430 | VIL2 | CVL; CVIL; MGC1584; FLJ26216; DKFZp762H157 |
| 218779_x_at | 0.327 | NM_017729 | 54869 | EPS8L1 | DRC3; EPS8R1; MGC4642; PP10566; FLJ20258; MGC23164; EPS8L1 |
| 209925_at | 0.328 | U53823 | 4950 | OCLN | OCLN |
| 240180_at | 0.328 | BE219849 |  | - |  |
| 205515_at | 0.328 | NM_003619 | 8492 | PRSS12 | MRT1; BSSP3; BSSP-3; MGC12722; MOTOPSIN |
| 231969_at | 0.328 | AB037813 | 56977 | STOX2 | DKFZp762K222 |
| 242354_at | 0.328 | AI191905 |  | - |  |
| 1553114_a_at | 0.329 | NM_005975 | 5753 | PTK6 | BRK; FLJ42088 |
| 238029_s_at | 0.329 | R15072 | 151473 | SLC16A14 | MCT14; FLJ30794 |
| 207030_s_at | 0.33 | NM_001321 | 1466 | CSRP2 | CRP2; LMO5; SmLIM |
| 229223_at | 0.331 | AI038402 | 4775 | NFATC3 | NFAT4; NFATX; NFATC3 |
| 227449_at | 0.333 | AI799018 | 2043 | EPHA4 | SEK; HEK8; TYRO1 |
| 205259_at | 0.333 | NM_000901 | 4306 | NR3C2 | MR; MCR; MLR; MGC133092 |
| 228587_at | 0.333 | BE504892 | 125206 | SLC5A10 | SGLT5; FLJ25217 |
| 203327_at | 0.333 | N22903 | 3416 | IDE | FLJ35968; INSULYSIN |
| 1568868_at | 0.334 | BC039307 | 339761 | CYP27C1 | FLJ16008 |
| 233687_s_at | 0.334 | AC011473 | 284366 | KLK9 | KLK8; KLKL3; KLK-L3 |
| 228108_at | 0.334 | AW274846 |  | - |  |
| 223427_s_at | 0.334 | AB032179 | 54566 | EPB41L4B | CG1; EHM2; FLJ21596; DKFZp761N1814; EPB41L4B |
| 1553834_at | 0.334 | NM_153264 | 256076 | COL6A5 | FLJ35880 |
| 235626_at | 0.335 | AA835485 | 57118 | CAMK1D | CKLiK; CaM-K1; CaMKID; CAMK1D |
| 225613_at | 0.335 | AI096389 | 23227 | MAST4 |  |
| 226959_at | 0.335 | AL137430 | 283070 | - |  |
| 209318_x_at | 0.336 | BG547855 | 5325 | PLAGL1 | ZAC; LOT1; ZAC1; MGC126275; MGC126276; DKFZp781P1017; PLAGL1 |
| 203888_at | 0.336 | NM_000361 | 7056 | THBD | TM; THRM; CD141 |
| 219232_s_at | 0.336 | NM_022073 | 112399 | EGLN3 | PHD3; HIFPH3; FLJ21620; MGC125998; MGC125999 |
| 205832_at | 0.337 | NM_016352 | 51200 | CPA4 | CPA3 |
| 205200_at | 0.337 | NM_003278 | 7123 | CLEC3B | TN; TNA; DKFZp686H17246 |
| 207431_s_at | 0.337 | NM_003676 | 8560 | DEGS1 | MLD; DEGS; DES1; Des-1; FADS7; MIG15; MGC5079; DEGS1 |
| 228090_at | 0.338 | AW249913 | 349565 | NMNAT3 | PNAT3; PNAT-3 |
| 232609_at | 0.338 | BC002652 | 92359 | CRB3 | CRB3 |
| 233575_s_at | 0.338 | AA705845 | 7091 | TLE4 | ESG; ESG4; GRG4; BCE-1; E(spI) |
| 216243_s_at | 0.339 | BE563442 | 3557 | IL1RN | IRAP; IL1F3; IL1RA; IL-1ra3; ICIL-1RA; MGC10430; IL1RN |
| 226231_at | 0.339 | AI189509 | 5074 | PAWR | PAR4; Par-4 |
| 244025_at | 0.339 | BF590917 |  | - |  |
| 219970_at | 0.341 | NM_017655 | 54810 | GIPC2 | SEMCAP2; FLJ20075; SEMCAP-2 |
| 206429_at | 0.342 | NM_005242 | 2150 | F2RL1 | PAR2; GPR11 |
| 234725_s_at | 0.342 | AK026133 | 10509 | SEMA4B | SemC; SEMAC; KIAA1745; MGC131831; SEMA4B |
| 228625_at | 0.342 | AI858001 | 163732 | CITED4 | CITED4 |
| 224839_s_at | 0.343 | BG328998 | 84706 | GPT2 | ALT2 |
| 207517_at | 0.344 | NM_018891 | 3918 | LAMC2 | B2T; EBR2; BM600; EBR2A; LAMB2T; LAMNB2; MGC138491; MGC141938; LAMC2 |
| 217080_s_at | 0.345 | Y19026 | 9455 | HOMER2 | CPD; ACPD; Vesl-2; HOMER-2; HOMER2A; HOMER2B |
| 219267_at | 0.345 | NM_016433 | 51228 | GLTP | GLTP |
| 202539_s_at | 0.345 | AL518627 | 3156 | HMGCR | HMGCR |
| 228954_at | 0.345 | AW452620 | 145748 | LYSMD4 | FLJ33008; MGC99501 |
| 206284_x_at | 0.346 | NM_001834 | 1212 | CLTB | LCB; CLTB |
| 204151_x_at | 0.346 | NM_001353 | 1645 | AKR1C1 | C9; DD1; DDH; DDH1; H-37; MBAB; HAKRC; MGC8954; 2-ALPHA-HSD; 20-ALPHA-HSD |
| 225612_s_at | 0.347 | BE672260 | 84002 | B3GNT5 | B3GN-T5; beta3Gn-T5 |
| 203476_at | 0.348 | NM_006670 | 7162 | TPBG | 5T4; M6P1; 5T4-AG |
| 230660_at | 0.349 | AU146709 | 56256 | SERTAD4 | DJ667H12.2 |
| 225299_at | 0.349 | AB032945 | 4645 | MYO5B | KIAA1119 |
| 219722_s_at | 0.349 | NM_024307 | 79153 | GDPD3 | MGC4171; FLJ22603; GDPD3 |
| 233302_at | 0.35 | AU146285 | 64919 | BCL11B | RIT1; CTIP2; CTIP-2; hRIT1-alpha; BCL11B |
| 214279_s_at | 0.35 | W74452 | 57447 | NDRG2 | SYLD; FLJ25522; KIAA1248; DKFZp781G1938; NDRG2 |
| 216733_s_at | 0.35 | X86401 | 2628 | GATM | AT; AGAT |
| 219369_s_at | 0.352 | NM_023112 | 78990 | OTUB2 | OTB2; OTU2; MGC3102; FLJ21916; C14orf137 |
| 241985_at | 0.353 | AI814405 | 133746 | JMY | JMY; FLJ37870; MGC163496 |
| 209357_at | 0.355 | AF109161 | 10370 | CITED2 | MRG1; P35SRJ |
| 218898_at | 0.355 | NM_024792 | 79850 | FAM57A | CT120; FLJ22282 |
| 217023_x_at | 0.355 | AF099143 | 7177 | TPSAB1 | TPS1; TPS2; TPSB1; alpha II |
| 206953_s_at | 0.356 | NM_012302 | 23266 | LPHN2 | CL2; LEC1; CIRL2; LPHH1 |
| 203081_at | 0.356 | NM_020248 | 56998 | CTNNBIP1 | ICAT; MGC15093; CTNNBIP1 |
| 207826_s_at | 0.356 | NM_002167 | 3399 | ID3 | HEIR-1 |
| 200862_at | 0.357 | NM_014762 | 1718 | DHCR24 | KIAA0018; SELADIN1; Nbla03646; seladin-1 |
| 207002_s_at | 0.357 | NM_002656 | 5325 | PLAGL1 | ZAC; LOT1; ZAC1; MGC126275; MGC126276; DKFZp781P1017; PLAGL1 |
| 224189_x_at | 0.358 | AF124438 | 26298 | EHF | ESE3; ESEJ |
| 214798_at | 0.358 | AW291664 | 9914 | ATP2C2 | SPCA2; KIAA0703; DKFZp686H22230 |
| 202546_at | 0.358 | NM_003761 | 8673 | VAMP8 | EDB; VAMP5 |
| 241782_at | 0.358 | AI932350 | 10529 | NEBL | LNEBL; bA56H7.1; MGC119746; MGC119747 |
| 231270_at | 0.358 | BF111998 | 377677 | CA13 | CAXIII; FLJ37995; MGC59868 |
| 211813_x_at | 0.36 | AF138303 | 1634 | DCN | CSCD; PG40; PGII; PGS2; DSPG2; SLRR1B; DCN |
| 201830_s_at | 0.36 | NM_005863 | 10276 | NET1 | NET1A; ARHGEF8 |
| 203216_s_at | 0.36 | NM_004999 | 4646 | MYO6 | DFNA22; DFNB37; KIAA0389 |
| 224799_at | 0.361 | AW290956 | 54602 | NDFIP2 | N4wbp5a; FLJ25842; KIAA1165 |
| 242055_at | 0.362 | AW136397 | 63027 | C6orf85 | FLJ22174; DKFZP434F011 |
| 205289_at | 0.362 | AA583044 | 650 | BMP2 | BMP2A |
| 213425_at | 0.363 | AI968085 | 7474 | WNT5A | hWNT5A |
| 229518_at | 0.363 | AA531023 | 115572 | FAM46B | MGC16491; MGC20845; RP11-344H11.8 |
| 209442_x_at | 0.363 | AL136710 | 288 | ANK3 | FLJ45464; ANKYRIN-G; ANK3 |
| 203887_s_at | 0.363 | NM_000361 | 7056 | THBD | TM; THRM; CD141 |
| 204255_s_at | 0.364 | AA772285 | 7421 | VDR | NR1I1; VDR |
| 211043_s_at | 0.364 | BC006332 | 1212 | CLTB | LCB; CLTB |
| 204519_s_at | 0.364 | NM_015993 | 51090 | PLLP | PMLP; TM4SF11 |
| 229374_at | 0.364 | AI758962 | 2043 | EPHA4 | SEK; HEK8; TYRO1 |
| 204059_s_at | 0.364 | NM_002395 | 4199 | ME1 | MES; HUMNDME |
| 220432_s_at | 0.365 | NM_016593 | 51302 | CYP39A1 | CYP39A1 |
| 238673_at | 0.365 | AV724325 |  | - |  |
| 213820_s_at | 0.365 | T54159 | 80765 | STARD5 | MGC10327 |
| 219429_at | 0.365 | NM_024306 | 79152 | FA2H | FAAH; FAXDC1; FLJ25287 |
| 204136_at | 0.365 | NM_000094 | 1294 | COL7A1 | EBD1; EBR1; EBDCT |
| 225001_at | 0.366 | AI744658 | 9545 | RAB3D | GOV; D2-2; RAB16; RAD3D |
| 204204_at | 0.366 | NM_001860 | 1318 | SLC31A2 | CTR2; COPT2; hCTR2 |
| 210119_at | 0.367 | U73191 | 3772 | KCNJ15 | IRKK; KIR1.3; KIR4.2; MGC13584; KCNJ15 |
| 211864_s_at | 0.367 | AF207990 | 26509 | FER1L3 | MYOF; FLJ36571; FLJ90777; FER1L3 |
| 206726_at | 0.369 | NM_014485 | 27306 | - | PGDS |
| 219496_at | 0.369 | NM_023016 | 65124 | ANKRD57 | C2orf26 |
| 233599_at | 0.369 | AK025151 | 84909 | C9orf3 | APO; AP-O; C90RF3; FLJ14675 |
| 1553764_a_at | 0.37 | NM_032876 | 84962 | JUB | Ajuba; MGC15563; JUB |
| 230523_at | 0.371 | AA603472 |  | - |  |
| 205027_s_at | 0.371 | NM_005204 | 1326 | MAP3K8 | COT; EST; ESTF; TPL2; Tpl-2; c-COT; FLJ10486 |
| 212848_s_at | 0.371 | BG036668 | 84909 | C9orf3 | APO; AP-O; C90RF3; FLJ14675 |
| 228988_at | 0.371 | AU157017 | 7552 | ZNF711 | ZNF4; ZNF5; ZNF6; CMPX1; Zfp711; dJ75N13.1 |
| 230266_at | 0.372 | AI127991 | 338382 | RAB7B | RAB7; MGC9726; MGC16212 |
| 226499_at | 0.372 | W72331 | 441478 | NRARP | NRARP; MGC61598 |
| 238455_at | 0.373 | AA329676 | 340895 | C10orf112 | bA265G8.2 |
| 219093_at | 0.374 | NM_017933 | 55022 | PID1 | NYGGF4; FLJ20701; HMFN2073 |
| 220149_at | 0.374 | NM_024861 | 79919 | C2orf54 | FLJ22671; MGC150431; MGC150432 |
| 225806_at | 0.374 | AI289311 | 84962 | JUB | Ajuba; MGC15563; JUB |
| 222236_s_at | 0.375 | AK000253 | 55616 | DDEFL1 | UPLC1; FLJ20199 |
| 219901_at | 0.376 | NM_018351 | 55785 | FGD6 | ZFYVE24 |
| 235976_at | 0.376 | AI680986 | 84189 | SLITRK6 | MGC119595; MGC119596; MGC119597 |
| 229327_s_at | 0.376 | BE674528 | 4094 | MAF | MGC71685; MAF |
| 202449_s_at | 0.376 | NM_002957 | 6256 | RXRA | NR2B1; FLJ16020; FLJ16733; MGC102720 |
| 207291_at | 0.377 | NM_024081 | 79056 | PRRG4 | TMG4 |
| 207126_x_at | 0.377 | NM_000463 | 54658; 54578; 54657; 54575; 54576; 54577; 54579; 54659; 54600 | GNT1; UGT1; UDPGT; UGT1A; HUG-BR1; HLUGP; UGT1F; HLUGP1; MGC29860 | GNT1; UGT1; UDPGT; UGT1A; HUG-BR1; HLUGP; UGT1F; HLUGP1; MGC29860; UGT1D; HUG-BR2; UGT1J; UGT1H; UGT1G; UGT1E; UGT1C; LUGP4; HLUGP4; UGT1AI; UGT1A6 |
| 223540_at | 0.377 | AF160477 | 81607 | PVRL4 | LNIR; PRR4; nectin-4 |
| 201473_at | 0.378 | NM_002229 | 3726 | JUNB | JUNB |
| 212741_at | 0.378 | AA923354 | 4128 | MAOA | MAOA |
| 219092_s_at | 0.378 | NM_022755 | 64768 | IPPK | C9orf12; INSP5K2; FLJ13163; KIAA0699; bA476B13.1 |
| 211834_s_at | 0.378 | AB042841 | 8626 | TP73L | KET; LMS; RHS; p51; p63; EEC3; OFC8; TP63; p73H; p73L; SHFM4; B(p51A); B(p51B) |
| 232000_at | 0.379 | AW001030 | 158219 | C9orf52 | FLJ33868 |
| 205668_at | 0.379 | NM_002349 | 4065 | LY75 | CD205; CLEC13B; DEC-205; GP200-MR6 |
| 205349_at | 0.379 | NM_002068 | 2769 | GNA15 | GNA16 |
| 212148_at | 0.38 | AL049381 | 5087 | PBX1 | MGC126627; DKFZp686B09108 |
| 1566232_at | 0.38 | AL832283 |  | - |  |
| 219503_s_at | 0.381 | NM_018306 | 55287 | TMEM40 | FLJ11036 |
| 209369_at | 0.381 | M63310 | 306 | ANXA3 | ANX3 |
| 212702_s_at | 0.382 | N45111 | 23299 | BICD2 | KIAA0699; bA526D8.1; BICD2 |
| 1554438_at | 0.382 | BC018764 | 56243 | KIAA1217 | SKT; MGC31990; DKFZP761L0424; RP11-324E23.1 |
| 57588_at | 0.382 | R62432 | 57419 | SLC24A3 | NCKX3 |
| 213832_at | 0.383 | AA530995 |  | - |  |
| 225133_at | 0.383 | AA130132 | 51274 | KLF3 | BKLF; MGC48279 |
| 202756_s_at | 0.384 | NM_002081 | 2817 | GPC1 | FLJ38078; glypican |
| 222372_at | 0.386 | AW971248 | 9223 | MAGI1 | AIP3; BAP1; WWP3; BAIAP1; MAGI-1; TNRC19; MAGI1 |
| 218312_s_at | 0.386 | NM_023926 | 65982 | ZSCAN18 | ZNF447; MGC2427; MGC4074; MGC8682; FLJ12895; FLJ44152; DKFZp586B1122 |
| 210461_s_at | 0.386 | BC002448 | 3983 | ABLIM1 | ABLIM; LIMAB1; LIMATIN; MGC1224; FLJ14564; KIAA0059; DKFZp781D0148; ABLIM1 |
| 227001_at | 0.386 | AI096706 | 79815 | NPAL2 | FLJ13955 |
| 212225_at | 0.386 | AL516854 | 10209 | EIF1 | A121; ISO1; SUI1; EIF-1; EIF1A |
| 226584_s_at | 0.387 | AL118502 | 83541 | FAM110A | F10; MGC2450; MGC4675; C20orf55; bA371L19.3; FAM110A |
| 203499_at | 0.387 | NM_004431 | 1969 | EPHA2 | ECK |
| 236207_at | 0.388 | BE083088 | 6744 | SSFA2 | CS1; CS-1; KRAP; SPAG13; FLJ45996; KIAA1927; DKFZp313O1039; DKFZp779G0129 |
| 242915_at | 0.388 | AA603590 | 91120 | ZNF682 | FLJ90362; BC39498_3 |
| 205870_at | 0.389 | NM_000623 | 624 | BDKRB2 | B2R; BK2; BK-2; BKR2; BRB2; DKFZp686O088 |
| 1559078_at | 0.389 | BM193618 | 53335 | BCL11A | EVI9; CTIP1; BCL11A-L; BCL11A-S; FLJ10173; FLJ34997; KIAA1809; BCL11A-XL |
| 243729_at | 0.39 | AI457984 |  | - |  |
| 227309_at | 0.39 | AI982535 | 55432 | YOD1 | OTUD2; PRO0907; DKFZp451J1719; RP11-164O23.1 |
| 211657_at | 0.39 | M18728 | 4680 | CEACAM6 | NCA; CEAL; CD66c |
| 209975_at | 0.391 | AF182276 | 1571 | CYP2E1 | CPE1; CYP2E; P450-J; P450C2E |
| 241994_at | 0.391 | BG260086 | 7498 | XDH | XO; XOR |
| 219313_at | 0.391 | NM_017577 | 54762 | GRAMD1C | FLJ35862; FLJ40464; DKFZp434C0328; DKFZp686K06117 |
| 220351_at | 0.393 | NM_016557 | 51554 | CCRL1 | PPR1; CCBP2; CCR10; CCR11; VSHK1; CKR-11; CCX-CKR; CC-CKR-11; CCRL1 |
| 213273_at | 0.393 | BF112171 | 26011 | ODZ4 |  |
| 234305_s_at | 0.393 | AJ245876 | 56169 | MLZE | MLZE |
| 1557458_s_at | 0.395 | BU685917 | 6461 | SHB | bA3J10.2; RP11-3J10.8 |
| 203385_at | 0.395 | NM_001345 | 1606 | DGKA | DAGK; DAGK1; MGC12821; MGC42356; DGK-alpha; DGKA |
| 200906_s_at | 0.395 | AK025843 | 23022 | PALLD | PNCA1; SIH002; CGI-151; FLJ22190; FLJ38193; FLJ39139; KIAA0992 |
| 236297_at | 0.395 | AI420817 | 340895 | C10orf112 | bA265G8.2 |
| 211986_at | 0.397 | BG287862 | 79026 | AHNAK | AHNAKRS; MGC5395 |
| 201798_s_at | 0.397 | NM_013451 | 26509 | FER1L3 | MYOF; FLJ36571; FLJ90777; FER1L3 |
| 238096_at | 0.397 | H13705 | 284023 | LOC284023 |  |
| 235857_at | 0.399 | AI859242 | 147040 | KCTD11 | REN; C17orf36; MGC129844; REN/KCTD11 |
| 1556221_a_at | 0.399 | BM992214 |  | - |  |
| 225314_at | 0.399 | BG291649 | 132299 | OCIAD2 | MGC45416; DKFZp686C03164; OCIAD2 |
| 236451_at | 0.4 | AA825510 |  | - |  |
| 224746_at | 0.4 | AB040955 | 57648 | KIAA1522 | KIAA1522 |
| 229419_at | 0.401 | BF222826 | 55294 | FBXW7 | AGO; CDC4; FBW7; FBX30; FBXW6; SEL10; SEL-10; DKFZp686F23254; FBXW7 |
| 227598_at | 0.402 | AI762857 | 113763 | C7orf29 | C7orf29 |
| 234932_s_at | 0.403 | AK026028 | 64866 | CDCP1 | CD318; TRASK; SIMA135; CDCP1 |
| 209733_at | 0.403 | AL034399 | 286440 | LOC286440 |  |
| 210117_at | 0.403 | AF311312 | 6674 | SPAG1 | SP75; TPIS; HSD-3.8; FLJ32920; SPAG1 |
| 225140_at | 0.403 | BF438116 | 51274 | KLF3 | BKLF; MGC48279 |
| 1564308_a_at | 0.403 | AK057360 | 143098 | MPP7 | FLJ32798; RP11-218D6.5 |
| 241703_at | 0.404 | AI928037 | 154661 | RUNDC3B | RPIB9; RPIP9; FLJ30671; MGC26655 |
| 209576_at | 0.404 | AL049933 | 2770 | GNAI1 | Gi |
| 211193_at | 0.404 | AF061512 | 8626 | TP73L | KET; LMS; RHS; p51; p63; EEC3; OFC8; TP63; p73H; p73L; SHFM4; B(p51A); B(p51B) |
| 201893_x_at | 0.404 | AF138300 | 1634 | DCN | CSCD; PG40; PGII; PGS2; DSPG2; SLRR1B; DCN |
| 210372_s_at | 0.404 | AF208012 | 7164 | TPD52L1 | D53; hD53; MGC8556; TPD52L2; TPD52L1 |
| 227623_at | 0.404 | H16409 |  | - |  |
| 219858_s_at | 0.404 | NM_017694 | 54842 | LOC154842 | FLJ20160 |
| 215425_at | 0.404 | AL049332 | 10950 | BTG3 | ANA; TOB5; TOFA; TOB55; MGC8928 |
| 1554966_a_at | 0.405 | AF329092 | 11259 | FILIP1L | DOC1; DOC-1; GIP90; FILIP1L |
| 238332_at | 0.405 | AI307802 | 147463 | ANKRD29 | FLJ25053 |
| 235146_at | 0.406 | N51717 | 57458 | TMCC3 | KIAA1145 |
| 204517_at | 0.407 | BE962749 | 5480 | PPIC | CYPC; MGC3673 |
| 225162_at | 0.407 | BG285417 | 152503 | SH3D19 | SH3D19; EBP; EVE1; MGC105136; MGC118910; MGC118911; MGC118912; MGC118913 |
| 213075_at | 0.407 | AL050002 | 169611 | OLFML2A | FLJ00237; PRO34319 |
| 209633_at | 0.407 | AL389975 | 5523 | PPP2R3A | PR72; PR130; PPP2R3; PPP2R3A |
| 206363_at | 0.407 | NM_005360 | 4094 | MAF | MGC71685; MAF |
| 205990_s_at | 0.408 | NM_003392 | 7474 | WNT5A | hWNT5A |
| 204612_at | 0.408 | NM_006823 | 5569 | PKIA | PRKACN1; PKIA |
| 210128_s_at | 0.408 | U41070 | 1241 | LTB4R | BLT1; BLTR; P2Y7; GPR16; LTBR1; P2RY7; CMKRL1; LTB4R1 |
| 241300_at | 0.409 | AA167323 |  | - |  |
| 216997_x_at | 0.41 | AL358975 | 7091 | TLE4 | ESG; ESG4; GRG4; BCE-1; E(spI) |
| 217518_at | 0.41 | BF056029 | 26509 | FER1L3 | MYOF; FLJ36571; FLJ90777; FER1L3 |
| 218559_s_at | 0.413 | NM_005461 | 9935 | MAFB | KRML; MGC43127 |
| 229657_at | 0.413 | BF431989 | 7068 | THRB | GRTH; THR1; ERBA2; NR1A2; THRB1; THRB2; ERBA-BETA; MGC126109; MGC126110 |
| 225262_at | 0.413 | AI670862 | 2355 | FOSL2 | FRA2; FLJ23306 |
| 202393_s_at | 0.414 | NM_005655 | 7071 | KLF10 | EGRA; TIEG; TIEG1; KLF10 |
| 1552631_a_at | 0.414 | NM_145319 | 9064 | MAP3K6 | ASK2; MAPKKK6; MGC20114; MGC125653 |
| 205538_at | 0.414 | NM_003389 | 7464 | CORO2A | IR10; WDR2; CLIPINB; CORO2A |
| 231240_at | 0.414 | AI038059 | 1734 | DIO2 | D2; 5DII; SelY; TXDI2; DIO2 |
| 229887_at | 0.415 | AI672049 | 259173 | ALS2CL | RN49018; MGC129698; DKFZp686P238; ALS2CL |
| 219054_at | 0.415 | NM_024563 | 79614 | C5orf23 | FLJ14054 |
| 208093_s_at | 0.416 | NM_030808 | 81565 | NDEL1 | EOPA; NUDEL; MITAP1; DKFZp451M0318; NDEL1 |
| 227641_at | 0.416 | AI613010 | 146330 | FBXL16 | Fbl16; C16orf22; FLJ33735; MGC33974; c380A1.1 |
| 208690_s_at | 0.416 | BC000915 | 9124 | PDLIM1 | CLIM1; CLP36; ELFIN; CLP-36; hCLIM1 |
| 231183_s_at | 0.417 | AI457817 | 182 | JAG1 | AGS; AHD; AWS; HJ1; CD339; JAGL1; MGC104644 |
| 204019_s_at | 0.417 | NM_015677 | 26751 | SH3YL1 | Ray; FLJ39121; DKFZP586F1318 |
| 226706_at | 0.418 | BE301252 | 200058 | - |  |
| 227306_at | 0.418 | AK024898 |  | - |  |
| 205240_at | 0.418 | NM_013296 | 29899 | GPSM2 | LGN; Pins |
| 226810_at | 0.418 | BE500942 |  | - |  |
| 227798_at | 0.418 | AU146891 | 4086 | SMAD1 | BSP1; JV41; JV4-1; MADH1; MADR1; SMAD1 |
| 219681_s_at | 0.418 | NM_025151 | 80223 | RAB11FIP1 | RCP; NOEL1A; FLJ22524; FLJ22622; MGC78448; rab11-FIP1; DKFZp686E2214; RAB11FIP1 |
| 213154_s_at | 0.418 | AI934125 | 23299 | BICD2 | KIAA0699; bA526D8.1; BICD2 |
| 210544_s_at | 0.42 | BC002430 | 224 | ALDH3A2 | SLS; FALDH; ALDH10; FLJ20851; DKFZp686E23276; ALDH3A2 |
| 204135_at | 0.42 | NM_014890 | 11259 | FILIP1L | DOC1; DOC-1; GIP90; FILIP1L |
| 236069_at | 0.42 | AI002328 | 284313 | - |  |
| 223194_s_at | 0.42 | AL512737 | 63027 | C6orf85 | FLJ22174; DKFZP434F011 |
| 210094_s_at | 0.421 | AF196186 | 56288 | PARD3 | Baz; ASIP; PAR3; PARD3A; Bazooka; SE2-5T2; FLJ21015; SE2-5L16; SE2-5LT1; PAR3alpha |
| 203697_at | 0.421 | U91903 | 2487 | FRZB | FRE; FZRB; hFIZ; FRITZ; FRP-3; FRZB1; SFRP3; SRFP3; FRZB-1; FRZB-PEN |
| 213135_at | 0.421 | U90902 | 7074 | TIAM1 | FLJ36302 |
| 219518_s_at | 0.421 | NM_025165 | 80237 | ELL3 | FLJ22637 |
| 205404_at | 0.421 | NM_005525 | 3290 | HSD11B1 | HDL; 11-DH; HSD11; HSD11B; HSD11L; MGC13539; 11-beta-HSD1; HSD11B1 |
| 221610_s_at | 0.421 | BC000795 | 55620 | STAP2 | STAP2; BKS; FLJ20234 |
| 204532_x_at | 0.422 | NM_021027 | 54658; 54578; 54657; 54575; 54576; 54577; 54579; 54659; 54600 | GNT1; UGT1; UDPGT; UGT1A; HUG-BR1; HLUGP; | GNT1; UGT1; UDPGT; UGT1A; HUG-BR1; HLUGP; UGT1F; HLUGP1; MGC29860; UGT1D; HUG-BR2; UGT1J; UGT1H; UGT1G; UGT1E; UGT1C; LUGP4; HLUGP4; UGT1AI; UGT1A6 |
| 202948_at | 0.422 | NM_000877 | 3554 | IL1R1 | P80; IL1R; IL1RA; CD121A; D2S1473; IL-1R-alpha |
| 226614_s_at | 0.422 | BE856336 | 83648 | C8orf13 | D8S265; MGC120649; MGC120650; MGC120651; DKFZp761G151 |
| 222802_at | 0.423 | J05008 | 1906 | EDN1 | ET1 |
| 204454_at | 0.423 | NM_012317 | 23641 | LDOC1 | Mar7; BCUR1; Mart7 |
| 203423_at | 0.423 | NM_002899 | 5947 | RBP1 | CRBP; RBPC; CRBP1; CRABP-I |
| 208614_s_at | 0.426 | M62994 | 2317 | FLNB | AOI; FH1; SCT; TAP; LRS1; TABP; FLN1L; ABP-278; filamin B; DKFZp686O033; DKFZp686A1668 |
| 205107_s_at | 0.426 | NM_005227 | 1945 | EFNA4 | EFL4; EPLG4; LERK4; MGC125826; EFNA4 |
| 214058_at | 0.427 | M19720 | 4610 | MYCL1 | LMYC; MYCL; MYCL1 |
| 210993_s_at | 0.427 | U54826 | 4086 | SMAD1 | BSP1; JV41; JV4-1; MADH1; MADR1; SMAD1 |
| 218813_s_at | 0.427 | NM_020145 | 56904 | SH3GLB2 | PP9455; KIAA1848 |
| 201161_s_at | 0.427 | NM_003651 | 8531 | CSDA | DBPA; CSDA1; ZONAB |
| 218986_s_at | 0.428 | NM_017631 | 55601 | DDX60 | FLJ20035; FLJ10787 |
| 235095_at | 0.428 | AW139399 | 146439 | CCDC64B | LOC146439; MGC158069 |
| 218751_s_at | 0.429 | NM_018315 | 55294 | FBXW7 | AGO; CDC4; FBW7; FBX30; FBXW6; SEL10; SEL-10; DKFZp686F23254; FBXW7 |
| 209348_s_at | 0.429 | AF055376 | 4094 | MAF | MGC71685; MAF |
| 223805_at | 0.43 | AF323728 | 114880 | OSBPL6 | ORP6; FLJ36583; MGC59642; OSBPL6 |
| 218373_at | 0.43 | NM_022476 | 64400 | AKTIP | FT1; FTS; AKTIP |
| 213256_at | 0.43 | AW593996 | 115123 | MARCH3 | RNF173; MGC48332; MARCH-III |
| 211965_at | 0.432 | BE620915 | 677 | ZFP36L1 | BRF1; ERF1; cMG1; ERF-1; Berg36; TIS11B; RNF162B |
| 201236_s_at | 0.432 | NM_006763 | 7832 | BTG2 | PC3; TIS21; MGC126063; MGC126064 |
| 231807_at | 0.432 | AL157473 | 56243 | KIAA1217 | SKT; MGC31990; DKFZP761L0424; RP11-324E23.1 |
| 202193_at | 0.433 | NM_005569 | 3985 | LIMK2 | LIMK2 |
| 204389_at | 0.433 | NM_000240 | 4128 | MAOA | MAOA |
| 202179_at | 0.433 | NM_000386 | 642 | BLMH | BH; BMH |
| 203317_at | 0.433 | NM_012455 | 23550 | PSD4 | TIC; EFA6B; FLJ36237; FLJ37279 |
| 213848_at | 0.434 | AI655015 | 1849 | DUSP7 | MKPX; MKP-X; PYST2 |
| 219915_s_at | 0.434 | NM_018593 | 117247 | SLC16A10 | TAT1; PRO0813 |
| 218739_at | 0.435 | NM_016006 | 51099 | ABHD5 | CDS; CGI58; IECN2; NCIE2; MGC8731 |
| 212706_at | 0.435 | AB011110 | 401331 | RASA4 | GAPL; CAPRI; KIAA0538; MGC131890; FLJ21767; DKFZp781H1351 |
| 226617_at | 0.436 | AW291264 | 26225 | ARL5A | ARL5; ARFLP5; ARL5A |
| 227276_at | 0.436 | AI264121 | 84898 | PLXDC2 | TEM7R; FLJ14623 |
| 226638_at | 0.437 | AV701292 | 57636 | ARHGAP23 | KIAA1501 |
| 219165_at | 0.437 | NM_021630 | 64236 | PDLIM2 | SLIM; FLJ34715; PDLIM2 |
| 226982_at | 0.437 | AI745624 | 22936 | ELL2 | ELL2 |
| 210375_at | 0.437 | X83858 | 5733 | PTGER3 | EP3; EP3e; EP3-I; EP3-II; EP3-IV; EP3-III; MGC27302; MGC141828; MGC141829; PTGER3 |
| 215554_at | 0.438 | AV699786 | 2822 | GPLD1 | GPIPLD; PIGPLD; GPIPLDM; PIGPLD1; MGC22590 |
| 207943_x_at | 0.438 | NM_006718 | 5325 | PLAGL1 | ZAC; LOT1; ZAC1; MGC126275; MGC126276; DKFZp781P1017; PLAGL1 |
| 236725_at | 0.438 | H09245 | 23286 | WWC1 | KIBRA; FLJ10865; FLJ23369; KIAA0869 |
| 228268_at | 0.438 | AI758223 | 2327 | FMO2 | FMO1B1; FLJ40826 |
| 244860_at | 0.438 | AW572853 | 23387 | SIK3 | KIAA0999; L19; FLJ12240 |
| 213238_at | 0.439 | AI478147 | 57205 | ATP10D | ATPVD; KIAA1487 |
| 205543_at | 0.439 | NM_014278 | 22824 | HSPA4L | APG-1; Osp94 |
| 204797_s_at | 0.439 | NM_004434 | 2009 | EML1 | EMAP; ELP79; EMAPL; HuEMAP; FLJ45033; EML1 |
| 209691_s_at | 0.44 | BC003541 | 55715 | DOK4 | FLJ10488 |
| 227209_at | 0.44 | AI091445 | 1272 | CNTN1 | F3; GP135; CNTN1 |
| 225367_at | 0.441 | BF512139 | 55276 | PGM2 | MSTP006; FLJ10983 |
| 202284_s_at | 0.441 | NM_000389 | 1026 | CDKN1A | P21; CIP1; SDI1; WAF1; CAP20; CDKN1; MDA-6; p21CIP1; CDKN1A |
| 227701_at | 0.442 | AK024739 | 55088 | C10orf118 | FLJ10188; FLJ35301; MGC118918 |
| 223497_at | 0.442 | AL136820 | 57579 | FAM135A | FLJ13577; KIAA1411; DKFZp781H2319 |
| 212538_at | 0.442 | AL576253 | 23348 | DOCK9 | ZIZ1; FLJ16744; FLJ44528; FLJ45282; FLJ45601; KIAA1058; KIAA1085; zizimin1; RP11-155N3.2; DKFZp686D2047; DKFZp686C11110; DKFZp686N04132 |
| 223658_at | 0.443 | AF134149 | 9424 | KCNK6 | TOSS; KCNK8; TWIK2; K2p6.1; TWIK-2; FLJ12282 |
| 223467_at | 0.443 | AF069506 | 51655 | RASD1 | AGS1; DEXRAS1; MGC:26290 |
| 205841_at | 0.444 | NM_004972 | 3717 | JAK2 | JAK2 |
| 225474_at | 0.444 | AI141556 | 9223 | MAGI1 | AIP3; BAP1; WWP3; BAIAP1; MAGI-1; TNRC19; MAGI1 |
| 220625_s_at | 0.444 | AF115403 | 2001 | ELF5 | ESE2; ELF5 |
| 215150_at | 0.445 | AF090896 | 55432 | YOD1 | OTUD2; PRO0907; DKFZp451J1719; RP11-164O23.1 |
| 227570_at | 0.445 | BE857226 | 144110 | TMEM86A | FLJ90119 |
| 213023_at | 0.446 | NM_007124 | 7402 | UTRN | DRP; DMDL; DRP1; FLJ23678 |
| 206637_at | 0.447 | NM_014879 | 9934 | P2RY14 | P2Y14; GPR105; KIAA0001 |
| 1560512_at | 0.448 | BQ027635 |  | - |  |
| 209372_x_at | 0.449 | BF971587 | 7280; 347733 | - | TUBB; TUBB2; dJ40E16.7; MGC8685; bA506K6.1; DKFZp566F223; RP11-506K6.1; TUBB-PARALOG |
| 225807_at | 0.449 | AK025567 | 84962 | JUB | Ajuba; MGC15563; JUB |
| 233130_at | 0.45 | AU147515 | 7068 | THRB | GRTH; THR1; ERBA2; NR1A2; THRB1; THRB2; ERBA-BETA; MGC126109; MGC126110 |
| 224853_at | 0.45 | AI979301 | 57606 | SLAIN2 | FLJ21611; KIAA1458 |
| 228378_at | 0.451 | BF439204 | 91298 | C12orf29 | FLJ38158; MGC102978; DKFZp313K0436; DKFZp434N2030; DKFZp686L04169 |
| 206094_x_at | 0.451 | NM_001072 | 54578 | UGT1A6 | GNT1; UGT1; HLUGP; UDPGT; UGT1F; HLUGP1; MGC29860; UGT1A6 |
| 239675_at | 0.451 | BE348537 | 283143 | - | LOC283143 |
| 219919_s_at | 0.452 | NM_018276 | 54961 | SSH3 | SSH-3; FLJ10928; FLJ20515 |
| 1556332_at | 0.453 | CA313430 |  | - |  |
| 225990_at | 0.454 | W72626 | 91653 | BOC | BOC |
| 208156_x_at | 0.454 | NM_031308 | 83481 | EPPK1 | EPIPL; EPIPL1 |
| 202668_at | 0.455 | BF001670 | 1948 | EFNB2 | HTKL; EPLG5; Htk-L; LERK5; MGC126226; MGC126227; MGC126228 |
| 203641_s_at | 0.455 | BF002844 | 22837 | COBLL1 | KIAA0977 |
| 219976_at | 0.455 | NM_015888 | 51361 | HOOK1 | HK1; MGC10642 |
| 227998_at | 0.456 | AA045184 | 140576 | S100A16 | AAG13; S100F; DT1P1A7; MGC17528 |
| 220809_at | 0.456 | NM_024912 | 79972 | - |  |
| 210374_x_at | 0.457 | D38300 | 5733 | PTGER3 | EP3; EP3e; EP3-I; EP3-II; EP3-IV; EP3-III; MGC27302; MGC141828; MGC141829; PTGER3 |
| 203430_at | 0.458 | NM_014320 | 23593 | HEBP2 | PP23; SOUL; C6orf34; C6ORF34B; KIAA1244; RP3-422G23.1 |
| 201250_s_at | 0.458 | NM_006516 | 6513 | SLC2A1 | GLUT; GLUT1; MGC141895; MGC141896 |
| 224787_s_at | 0.459 | AI333232 | 22931 | RAB18 | RAB18LI1 |
| 209632_at | 0.459 | AI760130 | 5523 | PPP2R3A | PR72; PR130; PPP2R3; PPP2R3A |
| 209146_at | 0.461 | AV704962 | 6307 | SC4MOL | DESP4; ERG25; MGC104344; SC4MOL |
| 229656_s_at | 0.462 | AA236463 | 400954 | - |  |
| 216268_s_at | 0.462 | U77914 | 182 | JAG1 | AGS; AHD; AWS; HJ1; CD339; JAGL1; MGC104644 |
| 231932_at | 0.462 | AI922337 | 80342 | TRAF3IP3 | T3JAM; FLJ44151; MGC117354; MGC163289; DJ434O14.3 |
| 213624_at | 0.464 | AA873600 | 10924 | SMPDL3A | ASM3A; ASML3a; FLJ20177; yR36GH4.1 |
| 226885_at | 0.464 | AI743880 |  | - |  |
| 1554878_a_at | 0.465 | BC009712 | 5825 | ABCD3 | ABC43; PMP70; PXMP1 |
| 205471_s_at | 0.466 | AW772082 | 1602 | DACH1 | DACH; FLJ10138; DACH1 |
| 213568_at | 0.467 | AI811298 | 116039 | OSR2 | FLJ90037 |
| 225283_at | 0.467 | AV701177 | 91947 | ARRDC4 | FLJ36045 |
| 206873_at | 0.468 | NM_001215 | 765 | CA6 | GUSTIN; MGC21256 |
| 230792_at | 0.469 | BE671210 | 158584 | FAAH2 | AMDD; FAAH-2; FLJ31204; RP11-479E16; RP11-479E16.1 |
| 226099_at | 0.469 | AI924426 | 22936 | ELL2 | ELL2 |
| 220987_s_at | 0.47 | NM_030952 | 56672 | AKIP1 | BCA3; AKIP1; SNARK; FLJ90349; DKFZP434J037; DKFZp686F01113; C11orf17 |
| 226425_at | 0.47 | AU144247 | 79745 | CLIP4 | RSNL2; FLJ21069; FLJ32705 |
| 225529_at | 0.47 | AI492175 | 116983 | CENTB5 | KIAA1716 |
| 219944_at | 0.47 | NM_024692 | 79745 | CLIP4 | RSNL2; FLJ21069; FLJ32705 |
| 226489_at | 0.473 | BG177562 | 57458 | TMCC3 | KIAA1145 |
| 219095_at | 0.473 | NM_005090 | 8681 | PLA2G4B | FLJ42498; HsT16992; cPLA2-beta |
| 219411_at | 0.474 | NM_024712 | 79767 | ELMO3 | CED12; CED-12; ELMO-3; FLJ13824 |
| 205054_at | 0.474 | NM_004543 | 4703 | NEB | NEM2; NEB177D; FLJ39568; FLJ39584; DKFZp686C1456 |
| 244332_at | 0.475 | AW974077 | 92 | ACVR2A | ACVR2; ACTRII |
| 206101_at | 0.475 | NM_001393 | 1842 | ECM2 | MGC126355; MGC126356 |
| 226247_at | 0.475 | AI346026 | 59338 | PLEKHA1 | TAPP1; PLEKHA1 |
| 209098_s_at | 0.476 | U61276 | 182 | JAG1 | AGS; AHD; AWS; HJ1; CD339; JAGL1; MGC104644 |
| 226197_at | 0.476 | AW173504 |  | - |  |
| 220945_x_at | 0.476 | NM_018050 | 54682 | MANSC1 | FLJ10298; LOH12CR3; 9130403P13Rik |
| 202704_at | 0.478 | AA675892 | 10140 | TOB1 | TOB; TROB; APRO6; PIG49; TROB1; MGC34446; MGC104792 |
| 204616_at | 0.478 | NM_006002 | 7347 | UCHL3 | UCHL3 |
| 228188_at | 0.479 | AI860150 | 2355 | FOSL2 | FRA2; FLJ23306 |
| 224534_at | 0.481 | AB059618 | 83999 | KREMEN1 | KRM1; KREMEN; KREMEM1; FLJ31863 |
| 232270_at | 0.481 | AL137535 | 84909 | C9orf3 | APO; AP-O; C90RF3; FLJ14675 |
| 220944_at | 0.482 | NM_020393 | 57115 | PGLYRP4 | PGRPIB; SBBI67; PGRP-Ibeta; PGLYRPIbeta |
| 241355_at | 0.482 | BF528433 | 55806 | HR | AU; ALUNC; HSA277165; HR |
| 209684_at | 0.484 | AL136924 | 54453 | RIN2 | RASSF4 |
| 229648_at | 0.484 | AW025358 | 9743 | ARHGAP32 | RICS; GRIT; GC-GAP; MGC1892; p250GAP; KIAA0712; p200RhoGAP |
| 215549_x_at | 0.484 | AC005587 | 442780 | - |  |
| 229635_at | 0.485 | AW043859 |  | - |  |
| 206036_s_at | 0.485 | NM_002908 | 5966 | REL | C-Rel |
| 225211_at | 0.485 | AW139723 | 84853 | - |  |
| 229404_at | 0.487 | AI086614 | 117581 | TWIST2 | DERMO1; MGC117334 |
| 241931_at | 0.487 | AI168338 | 7499 | XG | PBDX; MGC118758; MGC118759; MGC118760; MGC118761 |
| 209193_at | 0.487 | M24779 | 5292 | PIM1 | PIM |
| 237439_at | 0.487 | R45656 | 124739 | USP43 |  |
| 226464_at | 0.488 | BE348597 | 205428 | C3orf58 | MGC33365 |
| 239196_at | 0.488 | AI097229 | 118932 | ANKRD22 | MGC22805 |
| 239586_at | 0.488 | AA085776 | 84985 | FAM83A | BJ-TSA-9; MGC14128; FAM83A |
| 207749_s_at | 0.488 | NM_002718 | 5523 | PPP2R3A | PR72; PR130; PPP2R3; PPP2R3A |
| 210608_s_at | 0.489 | BC001899 | 2524 | FUT2 | SE; Se2; sej |
| 207992_s_at | 0.489 | NM_000480 | 272 | AMPD3 | AMPD3 |
| 1556006_s_at | 0.49 | BQ025347 | 1452 | CSNK1A1 | CK1; HLCDGP1; PRO2975; CSNK1A1 |
| 201136_at | 0.49 | NM_002668 | 5355 | PLP2 | A4; A4-LSB; MGC126187 |
| 203367_at | 0.492 | NM_007026 | 11072 | DUSP14 | MKP6; MKP-L |
| 1554608_at | 0.493 | BC028219 | 10618 | TGOLN2 | TGN38; TGN46; TGN48; TGN51; TTGN2; MGC14722 |
| 1560112_at | 0.493 | AK054833 | 115825 | WDFY2 | WDF2; ZFYVE22; RP11-147H23.1 |
| 209250_at | 0.495 | BC000961 | 8560 | DEGS1 | MLD; DEGS; DES1; Des-1; FADS7; MIG15; MGC5079; DEGS1 |
| 227038_at | 0.495 | AI963083 | 166929 | SGMS2 | SMS2; MGC26963 |
| 243338_at | 0.495 | AI674461 | 1452 | CSNK1A1 | CK1; HLCDGP1; PRO2975; CSNK1A1 |
| 210260_s_at | 0.496 | BC005352 | 25816 | TNFAIP8 | GG2-1; SCC-S2; MDC-3.13 |
| 228568_at | 0.496 | AI926697 | 145781 | GCOM1 | Gcom1; Gup1; Gup2; Gcom2; GRINL1A; FLJ30973; MGC126694; MGC138353 |
| 203238_s_at | 0.496 | NM_000435 | 4854 | NOTCH3 | CASIL; CADASIL |
| 202053_s_at | 0.496 | L47162 | 224 | ALDH3A2 | SLS; FALDH; ALDH10; FLJ20851; DKFZp686E23276; ALDH3A2 |
| 224685_at | 0.497 | AI675354 | 4301 | MLLT4 | AF6; AF-6; AFADIN; FLJ34371; RP3-431P23.3 |
| 204254_s_at | 0.497 | NM_000376 | 7421 | VDR | NR1I1; VDR |
| 207180_s_at | 0.498 | NM_006410 | 10553 | HTATIP2 | CC3; TIP30; FLJ26963 |
| 218174_s_at | 0.498 | NM_025125 | 80195 | C10orf57 | FLJ13263; bA369J21.6; RP11-369J21.6 |
| 223322_at | 0.498 | BC004270 | 83593 | RASSF5 | RAPL; Maxp1; NORE1; NORE1A; NORE1B; RASSF3; MGC10823; MGC17344; RASSF5 |
| 239660_at | 0.498 | BF110518 | 57186 | C20orf74 | C20orf74 |
| 227909_at | 0.499 | AI742434 | 399668 | - |  |
| 225105_at | 0.499 | BF969397 | 387882 | - |  |
| 1431_at | 0.499 | J02843 | 1571 | CYP2E1 | CPE1; CYP2E; P450-J; P450C2E |
