# Supplementary material for: Integrative Genomics Identifies Gene Signature Associated with Melanoma Ulceration
Source: PLoS One. 2013 Jan 30;8(1):e54958. doi: 10.1371/journal.pone.0054958 (PMC3559846; doi:10.1371/journal.pone.0054958)
Supplement: Table S2 — Upregulated genes (N = 93) in ulcerated melanomas. (DOC) [file pone.0054958.s004.doc]

**Table S2. Upregulated genes (N = 93) in ulcerated melanomas**

| **Probe set ID** | **Fold Change** | **GenBank ID** | **Entrez Gene ID** | **Gene Symbol** | **Common Name of Transcripts** |
| --- | --- | --- | --- | --- | --- |
| 209875_s_at | 5.416 | M83248 | 6696 | SPP1 | OPN; BNSP; BSPI; ETA-1; MGC110940 |
| 229178_at | 4.945 | AV699825 | 145786 | LOC145786 | LOC145786 |
| 209498_at | 4.153 | X16354 | 634 | CEACAM1 | BGP; BGP1; BGPI; CEACAM1 |
| 206617_s_at | 3.998 | NM_002910 | 5973 | RENBP | RBP; RNBP |
| 229073_at | 3.832 | AA912476 | 145786 | LOC145786 | LOC145786 |
| 205334_at | 3.808 | NM_006271 | 6271 | S100A1 | S100; S100A; S100-alpha |
| 232269_x_at | 3.752 | BE965311 | 79006 | METRN | MGC2601; C16orf23; c380A1.2 |
| 219051_x_at | 3.634 | NM_024042 | 79006 | METRN | MGC2601; C16orf23; c380A1.2 |
| 235238_at | 3.615 | BF676462 | 399694 | SHC4 | RaLP; MGC34023 |
| 221042_s_at | 3.471 | NM_024734 | 79789 | CLMN | FLJ12383; KIAA1188 |
| 204416_x_at | 3.373 | NM_001645 | 341 | APOC1 | APOC1 |
| 213638_at | 2.987 | AW054711 | 221692 | PHACTR1 | RPEL; RPEL1; KIAA1733; MGC126575; MGC126577; dJ257A7.2 |
| 202976_s_at | 2.953 | NM_014899 | 22836 | RHOBTB3 | KIAA0878 |
| 230538_at | 2.951 | AI027957 | 399694 | SHC4 | RaLP; MGC34023 |
| 202898_at | 2.941 | NM_014654 | 9672 | SDC3 | SDCN; SYND3; N-syndecan |
| 209505_at | 2.909 | AI951185 | 7025 | NR2F1 | EAR3; EAR-3; NR2F2; SVP44; ERBAL3; TFCOUP1; COUP-TFI; TCFCOUP1 |
| 241612_at | 2.9 | W57731 | 27022 | FOXD3 | HFH2; Genesis |
| 212190_at | 2.878 | AL541302 | 5270 | SERPINE2 | GDN; PI7; PN1; PNI |
| 221489_s_at | 2.874 | W48843 | 81848 | SPRY4 | SPRY4 |
| 233436_at | 2.867 | AK022122 | 27085 | MTBP | MDM2BP |
| 227196_at | 2.837 | BG054987 | 85415 | RHPN2 | RhoBP; p76RBE |
| 1554638_at | 2.796 | BC032227 | 9765 | ZFYVE16 | ENDOFIN; KIAA0305; DKFZp686E13162 |
| 215054_at | 2.77 | H16758 | 2057 | EPOR | MGC138358 |
| 213553_x_at | 2.765 | W79394 | 341 | APOC1 | APOC1 |
| 239090_at | 2.757 | BF110321 | 84140 | FAM161A | FLJ13305 |
| 203299_s_at | 2.735 | AF251295 | 8905 | AP1S2 | DC22; MRX59; SIGMA1B; MGC:1902 |
| 204044_at | 2.717 | NM_014298 | 23475 | QPRT | QPRTase |
| 227530_at | 2.709 | BF511276 | 9590 | AKAP12 | AKAP250; DKFZp686M0430; DKFZp686O0331; AKAP12 |
| 218529_at | 2.664 | NM_016579 | 51293 | CD320 | 8D6; 8D6A |
| 205119_s_at | 2.662 | NM_002029 | 2357 | FPR1 | FPR; FMLP |
| 242260_at | 2.625 | BG283790 | 9782 | MATR3 | MGC9105; KIAA0723; DKFZp686K0542; DKFZp686K23100; MATR3 |
| 222240_s_at | 2.614 | AL137749 | 51477 | ISYNA1 | ISYNA1 |
| 243588_at | 2.602 | N74058 | 10160 | FARP1 | CDEP; PLEKHC2; MGC87400; FARP1 |
| 243745_at | 2.599 | N74507 | 8905 | AP1S2 | DC22; MRX59; SIGMA1B; MGC:1902 |
| 204271_s_at | 2.545 | M74921 | 1910 | EDNRB | ETB; ETRB; HSCR; ABCDS; HSCR2; EDNRB |
| 224252_s_at | 2.541 | AF177940 | 53827 | FXYD5 | RIC; IWU1; KCT1; OIT2; IWU-1; dysad; HSPC113; PRO6241; FXYD5 |
| 236038_at | 2.489 | N50714 |  | - |  |
| 225202_at | 2.48 | BE620739 | 22836 | RHOBTB3 | KIAA0878 |
| 224341_x_at | 2.449 | U93091 | 7099 | TLR4 | TOLL; CD284; hToll |
| 228415_at | 2.414 | AA205444 | 8905 | AP1S2 | DC22; MRX59; SIGMA1B; MGC:1902 |
| 209842_at | 2.405 | AI367319 | 6663 | SOX10 | DOM; WS4; MGC15649 |
| 223679_at | 2.375 | AF130085 | 1499 | CTNNB1 | CTNNB; FLJ25606; FLJ37923; DKFZp686D02253 |
| 235371_at | 2.366 | AI452595 | 389129 | LOC389129 | LOC389129 |
| 203349_s_at | 2.357 | NM_004454 | 2119 | ETV5 | ERM |
| 237005_at | 2.353 | AI923935 | 442075 | LOC442075 | LOC442075 |
| 216813_at | 2.347 | AL512728 |  | - |  |
| 222771_s_at | 2.337 | BF224052 | 50804 | MYEF2 | MEF-2; MST156; MSTP156; FLJ11213; HsT18564; KIAA1341; MGC87325 |
| 1555772_a_at | 2.333 | AY137580 | 993 | CDC25A | CDC25A2 |
| 238756_at | 2.308 | AI860012 | 283431 | GAS2L3 | GAS2L3 |
| 228141_at | 2.29 | AA173223 | 166979 | CDC20B | FLJ37927; G6VTS76519 |
| 244313_at | 2.285 | AI052659 |  | - |  |
| 203651_at | 2.282 | NM_014733 | 9765 | ZFYVE16 | ENDOFIN; KIAA0305; DKFZp686E13162 |
| 201661_s_at | 2.276 | NM_004457 | 2181 | ACSL3 | ACS3; FACL3; PRO2194; ACSL3 |
| 217997_at | 2.267 | AI795908 | 22822 | PHLDA1 | PHRIP; TDAG51; DT1P1B11; MGC131738 |
| 200644_at | 2.252 | NM_023009 | 65108 | MARCKSL1 | F52; MLP; MRP; MLP1; MACMARCKS |
| 226670_s_at | 2.246 | AL109839 | 80336 | C20orf119 |  |
| 203908_at | 2.215 | NM_003759 | 8671 | SLC4A4 | KNBC; NBC1; NBC2; pNBC; HNBC1; hhNMC; SLC4A5; DKFZp781H1314 |
| 202478_at | 2.212 | NM_021643 | 28951 | TRIB2 | TRB2; GS3955 |
| 212658_at | 2.205 | N66633 | 10184 | LHFPL2 | KIAA0206; DKFZp781E0375 |
| 224822_at | 2.196 | AA524250 | 10395 | DLC1 | HP; ARHGAP7; STARD12; FLJ21120; p122-RhoGAP; DLC1 |
| 206896_s_at | 2.195 | NM_005145 | 2788 | GNG7 | FLJ00058 |
| 236798_at | 2.192 | AW268719 |  | - |  |
| 212354_at | 2.179 | BE500977 | 23213 | SULF1 | SULF-1; HSULF-1; FLJ30905; FLJ38022; FLJ41750; KIAA1077 |
| 201660_at | 2.178 | AL525798 | 2181 | ACSL3 | ACS3; FACL3; PRO2194; ACSL3 |
| 226592_at | 2.166 | AA031404 | 286334 | - |  |
| 204671_s_at | 2.165 | BE677131 | 22881 | ANKRD6 | ANKRD6 |
| 217771_at | 2.159 | NM_016548 | 51280 | GOLPH2 | GP73; PSEC0257; GOLPH2 |
| 212884_x_at | 2.159 | AI358867 | 348 | APOE | AD2; MGC1571; apoprotein |
| 235709_at | 2.159 | H37811 | 283431 | GAS2L3 | GAS2L3 |
| 221974_at | 2.152 | AW770748 | 6638 | SNRPN | SMN; SM-D; RT-LI; HCERN3; SNRNP-N; SNURF-SNRPN |
| 238444_at | 2.152 | AV704303 | 114991 | ZNF618 | FP13169 |
| 223469_at | 2.148 | BC004942 | 83542 | MGC10812 | MGC10812 |
| 225726_s_at | 2.147 | AB033026 | 57475 | PLEKHH1 | PLEKHH1 |
| 205880_at | 2.121 | NM_002742 | 5587 | PRKD1 | PKD; PKCM; PRKCM; PKC-MU |
| 223241_at | 2.105 | AF121858 | 29886 | SNX8 | SNX8 |
| 239331_at | 2.102 | AW954199 |  | - |  |
| 229464_at | 2.087 | N50034 | 50804 | MYEF2 | MEF-2; MST156; MSTP156; FLJ11213; HsT18564; KIAA1341; MGC87325 |
| 1556051_a_at | 2.081 | CA777994 | 636 | BICD1 | BICD; BICD1 |
| 205447_s_at | 2.077 | BE222201 | 7786 | MAP3K12 | DLK; MUK; ZPK; ZPKP1 |
| 243618_s_at | 2.076 | BF678830 | 152485 | ZNF827 | LOC152485 |
| 204015_s_at | 2.061 | BC002671 | 1846 | DUSP4 | TYP; HVH2; MKP2; MKP-2; DUSP4 |
| 225237_s_at | 2.042 | BF435123 | 124540 | MSI2 | MSI2H; MGC3245; FLJ36569 |
| 201272_at | 2.028 | NM_001628 | 231 | AKR1B1 | AR; ADR; ALDR1; MGC1804 |
| 203217_s_at | 2.025 | NM_003896 | 8869 | ST3GAL5 | SIAT9; ST3GalV; SIATGM3S |
| 240859_at | 2.023 | N20928 | 9765 | ZFYVE16 | ENDOFIN; KIAA0305; DKFZp686E13162 |
| 205099_s_at | 2.022 | NM_001295 | 1230 | CCR1 | CD191; CKR-1; HM145; CMKBR1; MIP1aR; SCYAR1 |
| 230102_at | 2.02 | AW206458 | 2119 | ETV5 | ERM |
| 226017_at | 2.012 | AI708432 | 112616 | CMTM7 | CKLFSF7; FLJ30992; CMTM7 |
| 207283_at | 2.011 | NM_020217 | 56969 | RPL23AP32 | RPL23AP13; DKFZp547I014 |
| 243492_at | 2.009 | AW972653 | 117145 | THEM4 | CTMP; MGC29636 |
| 209435_s_at | 2.007 | BC000265 | 9181 | ARHGEF2 | GEF; P40; GEFH1; LFP40; GEF-H1; KIAA0651; DKFZp547L106; DKFZp547P1516 |
| 242879_x_at | 2.004 | AI939442 | 10000 | AKT3 | PKBG; PRKBG; STK-2; RAC-gamma; RAC-PK-gamma; DKFZP434N0250; AKT3 |
| 232676_x_at | 2.001 | AK002075 | 50804 | MYEF2 | MEF-2; MST156; MSTP156; FLJ11213; HsT18564; KIAA1341; MGC87325 |
