# Supplementary material for: Integrative Genomics Identifies Gene Signature Associated with Melanoma Ulceration
Source: PLoS One. 2013 Jan 30;8(1):e54958. doi: 10.1371/journal.pone.0054958 (PMC3559846; doi:10.1371/journal.pone.0054958)
Supplement: Table S3 — Genes for which expression significantly positively correlate with copy number changes in ulcerated melanomas. (DOC) [file pone.0054958.s005.doc]

**Table S3. Genes for which expression significantly positively correlate with copy number changes in ulcerated melanomas**

| **Cytogenetic Location** | **Gene Symbol** | **Pearson's r** |
| --- | --- | --- |
| 1p13.3 | VAV3 | 0.41 |
| 1p22.2 | GBP6 | 0.34 |
| 1p22.3 | CLCA2 | 0.34 |
| 1p31.1 | PTGER3 | 0.58 |
| 1p31.1 | LPHN2 | 0.35 |
| 1p31.1 | GIPC2 | 0.45 |
| 1p31.3 | INADL | 0.57 |
| 1p32.1 | HOOK1 | 0.43 |
| 1p35.1 | KIAA1522 | 0.46 |
| 1p36.11 | GRHL3 | 0.34 |
| 1p36.12 | CDA | 0.37 |
| 1p36.13 | PADI1 | 0.38 |
| 1q21.3 | RPTN | 0.67 |
| 1q32.1 | PKP1 | 0.35 |
| 1q32.1 | C1orf106 | 0.37 |
| 1q32.2 | C1orf116 | 0.42 |
| 1q32.3 | HSD11B1 | 0.35 |
| 1q32.3 | LAMB3 | 0.41 |
| 1q32.3 | SERTAD4 | 0.43 |
| 1q42.12 | DEGS1 | 0.36 |
| 2p13.3 | CD207 | 0.55 |
| 2p16.1 | REL | 0.53 |
| 2p16.1 | BCL11A | 0.34 |
| 2p23.1 | XDH | 0.73 |
| 2p23.2 | CLIP4 | 0.41 |
| 2p24.1 | SDC1 | 0.38 |
| 2p25.1 | GRHL1 | 0.38 |
| 2q11.2 | IL1R2 | 0.33 |
| 2q13 | IL1RN | 0.67 |
| 2q13 | IL1F7 | 0.33 |
| 2q13 | IL1F9 | 0.48 |
| 2q23.3 | NEB | 0.33 |
| 2q31.3 | SSFA2 | 1.00 |
| 2q32.2 | FLJ20160 | 0.33 |
| 2q35 | ABCA12 | 0.36 |
| 2q36.1 | EPHA4 | 0.37 |
| 2q36.3 | PID1 | 0.57 |
| 3p14.1 | MAGI1 | 0.33 |
| 3p14.3 | FLNB | 0.39 |
| 3p14.3 | WNT5A | 0.31 |
| 3p21.31 | SEMA3F | 0.35 |
| 3q13.31 | GRAMD1C | 0.40 |
| 3q21.1 | CSTA | 0.63 |
| 3q21.3 | COL29A1 | 0.55 |
| 3q22.1 | ACPP | 0.57 |
| 3q22.2 | PPP2R3A | 0.44 |
| 3q23 | RBP1 | 0.44 |
| 3q24 | C3orf58 | 0.36 |
| 3q25.1 | TM4SF1 | 0.46 |
| 3q26.31 | TNFSF10 | 0.54 |
| 3q27.1 | LAMP3 | 0.54 |
| 3q28 | TP63 | 1.00 |
| 4p14 | CENTD1 | 0.46 |
| 4p15.32 | FGFBP1 | 0.39 |
| 4p16.1 | SORCS2 | 0.50 |
| 4q12 | HOPX | 0.51 |
| 4q21.21 | RASGEF1B | 0.43 |
| 4q31.21 | SMAD1 | 0.49 |
| 4q32.3 | SC4MOL | 0.30 |
| 4q32.3 | PALLD | 0.45 |
| 5p13.3 | PDZD2 | 0.31 |
| 5q12.1 | ELOVL7 | 0.55 |
| 5q15 | ELL2 | 0.35 |
| 5q31.1 | CXCL14 | 0.64 |
| 5q32 | CSNK1A1 | 0.46 |
| 5q32 | SPINK5 | 0.46 |
| 5q32 | CSNK1A1 | 0.46 |
| 5q35.2 | CLTB | 0.61 |
| 6p25.2 | SLC22A23 | 0.37 |
| 6q14 | ELOVL4 | 0.36 |
| 6q14 | ME1 | 0.32 |
| 6q14 | TPBG | 0.59 |
| 6q21 | AIM1 | 0.35 |
| 6q22.31 | GJA1 | 0.47 |
| 6q22.31 | TPD52L1 | 0.40 |
| 6q23.3 | IL20RA | 0.77 |
| 6q23.3 | PERP | 0.50 |
| 6q25.1 | PPP1R14C | 0.56 |
| 7q22.1 | CYP3A5 | 0.64 |
| 7q35 | ARHGEF5 | 0.35 |
| 8p23.1 | DEFB1 | 0.46 |
| 8p23.1 | SOX7 | 0.60 |
| 8q22.1 | RBM35A | 1.00 |
| 8q22.2 | NPAL2 | 0.50 |
| 8q22.2 | OSR2 | 0.40 |
| 9p13.3 | PRSS3 | 0.32 |
| 9q21.12 | TJP2 | 0.45 |
| 9q22.32 | BICD2 | 0.46 |
| 9q31.3 | PTPN3 | 0.34 |
| 9q33.1 | BSPRY | 0.40 |
| 9q34.3 | RXRA | 0.31 |
| 10p11.22 | PARD3 | 0.53 |
| 10p11.23 | MAP3K8 | 0.34 |
| 10p12.1 | MPP7 | 0.56 |
| 10p12.31 | NEBL | 0.78 |
| 10p13 | CCDC3 | 0.53 |
| 10p14 | GATA3 | 0.48 |
| 10p15.1 | AKR1C1 | 1.00 |
| 10q23.1 | C10orf99 | 0.48 |
| 10q23.31 | ANKRD22 | 0.30 |
| 10q23.33 | PDLIM1 | 0.55 |
| 10q25.3 | ABLIM1 | 0.83 |
| 10q26.13 | TACC2 | 0.69 |
| 10q26.13 | PLEKHA1 | 0.46 |
| 10q26.3 | CYP2E1 | 0.37 |
| 11p13 | EHF | 0.42 |
| 11p15.4 | AMPD3 | 0.40 |
| 11p15.5 | TMEM16J | 0.35 |
| 11q13.2 | ALDH3B2 | 0.47 |
| 11q13.2 | RHOD | 0.58 |
| 11q22.3 | ICEBERG | 0.56 |
| 11q23.3 | MPZL2 | 0.58 |
| 12q21.2 | CSRP2 | 0.43 |
| 12q21.2 | PAWR | 0.39 |
| 13q12.11 | GJB6 | 0.42 |
| 13q22.1 | KLF5 | 0.42 |
| 13q31.1 | NDFIP2 | 0.34 |
| 14q11.2 | TGM1 | 0.40 |
| 14q11.2 | NDRG2 | 0.69 |
| 14q11.2 | JUB | 0.56 |
| 14q13.1 | EGLN3 | 0.34 |
| 14q24.1 | ZFP36L1 | 0.49 |
| 14q31.1 | STON2 | 0.52 |
| 14q32.2 | EML1 | 0.59 |
| 14q32.33 | AHNAK2 | 0.39 |
| 15q15.2 | TGM5 | 0.37 |
| 15q22.2 | CA12 | 1.00 |
| 15q25.2 | BNC1 | 0.32 |
| 15q26.3 | LYSMD4 | 0.66 |
| 16q22.1 | NFATC3 | 0.56 |
| 17p11.2 | ALDH3A1 | 0.36 |
| 17p13.1 | NDEL1 | 0.77 |
| 17q21.2 | JUP | 0.53 |
| 17q21.2 | KRT16 | 0.38 |
| 17q21.2 | KRT23 | 0.75 |
| 18q12.1 | DSG3 | 0.39 |
| 18q21.1 | MYO5B | 0.60 |
| 18q21.33 | SERPINB13 | 0.51 |
| 18q21.33 | SERPINB12 | 0.31 |
| 19p12 | ZNF682 | 0.37 |
| 19p13.12 | SLC1A6 | 0.33 |
| 19p13.2 | RAB3D | 0.62 |
| 19q13.12 | DMKN | 0.52 |
| 19q13.2 | SPINT2 | 0.58 |
| 19q13.41 | KLK5 | 0.39 |
| 19q13.42 | EPS8L1 | 0.47 |
| 20p11.23 | RIN2 | 0.34 |
| 20p13 | SDCBP2 | 0.46 |
| 20q11.22 | FAM83C | 0.36 |
| 22q12.2 | LIMK2 | 0.41 |
