# Supplementary material for: Integrative Genomics Identifies Gene Signature Associated with Melanoma Ulceration
Source: PLoS One. 2013 Jan 30;8(1):e54958. doi: 10.1371/journal.pone.0054958 (PMC3559846; doi:10.1371/journal.pone.0054958)
Supplement: Text S1 — High quality total RNA and DNA isolation, quality controls. (DOCX) [file pone.0054958.s006.docx]

**Text S1**

**High quality total RNA and DNA isolation, quality controls**

After surgical excision, fresh tissues were immediately placed in RNAlater solution (Applied Biosystems**,** Foster City, USA). Tumour diagnoses were made based on formalin-fixed paraffin-embedded tissue sections using hematoxylin and eosin staining.

High-quality total RNA was prepared from primary melanoma tissues using the RNeasy Mini kit according to the protocol of the supplier (Qiagen, GmbH, Germany). The obtained RNA concentrations were measured using a NanoDrop ND-1000 UV-Vis Spectrophotometer (NanoDrop Technologies, Wilmington, Delaware, USA). RNA sample integrity was determined with the Agilent 2100 Bioanalyser using the RNA 6000 Nano Kit (Agilent Technologies, Palo Alto, CA, USA). All RNA samples exhibited a 28S/18S ribosomal RNA ratio greater than 1.5. To ensure that the RNA samples were free from biological inhibitors that could suppress the reverse transcription step, p16 (CDKN2A) gene-specific QRT-PCR was performed for all samples. The G-spin™ Genomic DNA Extraction Kit (Intron, Korea) was used to isolate high molecular weight DNA from 17 primary melanomas according to the protocol provided by the manufacturer. To determine the quantity of DNA obtained, we used a NanoDrop ND-1000 UV-Vis Spectrophotometer (NanoDrop Technologies, Wilmington, Delaware USA). DNA integrity was verified via 1.2% agarose gel electrophoresis.
