# Supplementary material for: Integrative Genomics Identifies Gene Signature Associated with Melanoma Ulceration
Source: PLoS One. 2013 Jan 30;8(1):e54958. doi: 10.1371/journal.pone.0054958 (PMC3559846; doi:10.1371/journal.pone.0054958)
Supplement: Text S2 — Evaluation of cis- and trans acting copy number alterations. (DOCX) [file pone.0054958.s007.docx]

**Text S2**

**Evaluation of cis- and trans acting copy number alterations**

In order to identify both cis- and trans-acting CNVs, we performed an L1-constrained regression (Lasso-regression) on the important genes that was identified previously. This method assumes that the predictor variables are independent, so first we implemented a dimensionality reduction with the help of the „CGHregions” R package. This algorithm results in CNV regions, in which the clones are very much alike, with minimal information loss. The L1-constrained regression was performed with the „lol” R package using cross-validation optimizer. The resulted significant CNV regions were identified as cis-acting elements if they were on the same chromosome or were closer than 50 Mb to the investigated gene and as trans-acting elements otherwise. We assessed the score, which represents dependency of the response variable from the predictors, using the following equitations.

$${score}_{i}=-ln\left( \frac{\sigma_{with all}^{2}}{\sigma_{without all}^{2}} \right)$$

$${score}_{i}^{cis}=-ln\left( \frac{\sigma_{with cis}^{2}}{\sigma_{without all}^{2}} \right)$$

$${score}_{i}^{trans}=-ln\left( \frac{\sigma_{with cis}^{2}-\sigma_{with all}^{2}}{\sigma_{without all}^{2}} \right)$$

where

$\sigma_{with all}^{2}$ represents the variance of the prediction residuals of the model with all significant predictors included

$\sigma_{with cis}^{2}$ represents the variance of the prediction residuals of the model with only the significant cis predictors included

$\sigma_{without all}^{2}$ represents the variance of the prediction residuals of the model without the significant predictors
